# Effect of tobacco control policies on perinatal and child health: a systematic review and meta-analysis

**DOI:** 10.1016/S2468-2667(17)30144-5

**Published:** 2017-09-05

**Authors:** Timor Faber, Arun Kumar, Johan P Mackenbach, Christopher Millett, Sanjay Basu, Aziz Sheikh, Jasper V Been

**Affiliations:** a Division of Neonatology, Erasmus University Medical Centre—Sophia Children's Hospital, Rotterdam, Netherlands; b Department of Paediatrics, Erasmus University Medical Centre—Sophia Children's Hospital, Rotterdam, Netherlands; c Department of Obstetrics and Gynaecology, Erasmus University Medical Centre—Sophia Children's Hospital, Rotterdam, Netherlands; d Department of Public Health, Erasmus University Medical Centre, Rotterdam, Netherlands; e Centre of Medical Informatics, Usher Institute of Population Health Sciences and Informatics, University of Edinburgh, Edinburgh, UK; f Public Health Policy Evaluation Unit, School of Public Health, Imperial College London, London, UK; g Prevention Research Center, Stanford University, Stanford, CA, USA; h Division of General Internal Medicine and Primary Care, Brigham and Women's Hospital/Harvard Medical School, Boston, MA, USA; i Department of Medicine, Harvard Medical School, Boston, MA, USA

## Abstract

**Background:**

Tobacco smoking and smoke exposure during pregnancy and childhood cause considerable childhood morbidity and mortality. We did a systematic review and meta-analysis to investigate whether implementation of WHO's recommended tobacco control policies (MPOWER) was of benefit to perinatal and child health.

**Methods:**

We searched 19 electronic databases, hand-searched references and citations, and consulted experts to identify studies assessing the association between implementation of MPOWER policies and child health. We did not apply any language restrictions, and searched the full time period available for each database, up to June 22, 2017. Our primary outcomes of interest were perinatal mortality, preterm birth, hospital attendance for asthma exacerbations, and hospital attendance for respiratory tract infections. Where possible and appropriate, we combined data from different studies in random-effects meta-analyses. This study is registered with PROSPERO, number CRD42015023448.

**Findings:**

We identified 41 eligible studies (24 from North America, 16 from Europe, and one from China) that assessed combinations of the following MPOWER policies: smoke-free legislation (n=35), tobacco taxation (n=11), and smoking cessation services (n=3). Risk of bias was low in 23 studies, moderate in 16, and high in two. Implementation of smoke-free legislation was associated with reductions in rates of preterm birth (–3·77% [95% CI −6·37 to −1·16]; ten studies, 27 530 183 individuals), rates of hospital attendance for asthma exacerbations (–9·83% [–16·62 to −3·04]; five studies, 684 826 events), and rates of hospital attendance for all respiratory tract infections (–3·45% [–4·64 to −2·25]; two studies, 1 681 020 events) and for lower respiratory tract infections (–18·48% [–32·79 to −4·17]; three studies, 887 414 events). Associations appeared to be stronger when comprehensive smoke-free laws were implemented than when partial smoke-free laws were implemented. Among two studies assessing the association between smoke-free legislation and perinatal mortality, one showed significant reductions in stillbirth and neonatal mortality but did not report the overall effect on perinatal mortality, while the other showed no change in perinatal mortality. Meta-analysis of studies on other MPOWER policies was not possible; all four studies on increasing tobacco taxation and one of two on offering disadvantaged pregnant women help to quit smoking that reported on our primary outcomes had positive findings. Assessment of publication bias was only possible for studies assessing the association between smoke-free legislation and preterm birth, showing some degree of bias.

**Interpretation:**

Smoke-free legislation is associated with substantial benefits to child health. The majority of studies on other MPOWER policies also indicated a positive effect. These findings provide strong support for implementation of such policies comprehensively across the world.

**Funding:**

Chief Scientist Office Scotland, Farr Institute, Netherlands Lung Foundation, Erasmus MC.

## Introduction

Almost half of children worldwide are regularly exposed to second-hand smoke, and 28% of the 600 000 deaths each year related to second-hand smoke occur in children.^1^, ^2^ Maternal smoking and second-hand smoke exposure during pregnancy are detrimental to fetal growth and development, leading to adverse birth outcomes such as preterm birth, low birthweight, being small for gestational age, and perinatal and infant mortality.^3^, ^4^, ^5^, ^6^, ^7^, ^8^ Additionally, second-hand smoke exposure presents substantial health risks postnatally by increasing the risk of asthma and respiratory tract infections.^1^, ^9^

Protection of children from the adverse health implications of second-hand smoke during important phases of development and the subsequent disease burden carried on into adulthood is crucial. The WHO Framework Convention on Tobacco Control (FCTC) aims to reduce tobacco consumption and second-hand smoke exposure through national tobacco control programmes.^2^ In 2008, six MPOWER measures were introduced to guide FCTC implementation (panel).^2^, ^10^ With tobacco use increasingly becoming a problem of developing countries already experiencing the largest burden of early-life morbidity and mortality, the absence of tobacco regulation is set to be a big driver of between-country inequality in child health outcomes.^11^ However, evaluations of the effectiveness of tobacco control interventions have generally excluded children, focusing instead on smoking rates and adult health outcomes.^12^, ^13^, ^14^

Panel **MPOWER policies2**

**Monitor tobacco use**

Eligible policies include those that enforce accurate measurement of the extent of the tobacco epidemic and of the interventions to control it.

**Protect people from smoke**

Eligible policies include legislation to create smoke-free public environments (both indoors and outdoors).

**Offer help to quit tobacco use**

Eligible policies include tobacco cessation advice or interventions offered through health-care services, free telephone quit lines, and providing access to free or low-cost cessation medicines.

**Warn about the dangers of tobacco**

Eligible policies include health warnings on tobacco products, plain packaging of tobacco products, and mass media campaigns to educate the public about the dangers of tobacco.

**Enforce bans on tobacco advertising, promotion and sponsorship**

See WHO Framework Convention on Tobacco Control (FCTC) guidelines for implementation of Article 13, which provides a non-exhaustive list of advertising, promotion, and sponsorship within the terms of the FCTC.^11^

**Raise taxes on tobacco**

Eligible policies include increasing percentage excise tax share in final tobacco.

**Research in context**

**Evidence before this study**

Tobacco smoke exposure is the world's leading cause of preventable morbidity and premature mortality. Children cannot control their tobacco smoke exposure and therefore need protection through tobacco control measures. In a previous systematic review, we investigated the associations between smoke-free legislation and perinatal and child health outcomes. We searched 14 online medical research databases, the WHO International Clinical Trials Registry Platform, hand-searched references and citations, and consulted a panel of experts in the field to identify published and unpublished literature in any language from January, 1975, to May, 2013, on the associations between smoke-free legislation and our outcomes of interest. The primary outcomes were preterm birth, low birthweight, and hospital attendance for asthma. We identified 11 studies showing that smoke-free legislation was associated with significant reductions in preterm birth and severe asthma exacerbations. Studies have since addressed various knowledge gaps identified in our previous review, including assessments of the effect of smoke-free legislation on respiratory tract infections, the most important contributor to the global burden of paediatric morbidity and mortality associated with tobacco smoke exposure. The increased number of studies now available was also anticipated to allow investigation of another knowledge gap: exploration of a potential dose–response association between the comprehensiveness of smoke-free laws and their effect on child health. Furthermore, we sought to substantially broaden the focus of our study by evaluating the early-life health effect of the entire range of WHO-recommended tobacco control policies (ie, MPOWER). Following a prespecified and peer-reviewed protocol, we did a comprehensive literature search for experimental and quasi-experimental studies assessing associations between implementation of MPOWER policies and key perinatal and childhood outcomes associated with tobacco smoke exposure.

**Added value of this study**

To our knowledge, this is the first systematic review examining the association between the full spectrum of MPOWER policies and perinatal and child health. Our findings add value to the existing evidence base by identifying a link between smoke-free legislation and a substantial reduction in severe paediatric respiratory tract infections, providing consistent evidence that comprehensive smoke-free laws are associated with broad health effects, and collating evidence supporting the potential for other MPOWER measures to benefit child health. We also identified several key knowledge gaps, including a shortage of studies in low-income and middle-income countries, and of studies assessing MPOWER measures other than smoke-free legislation, tobacco tax increases, and smoking cessation services.

**Implications of all the available evidence**

With most of the world's population currently not covered by comprehensive tobacco control policies, there is great potential for global public health gains by protecting unborn babies and children from tobacco smoke exposure. Future efforts should focus on increasing the uptake of comprehensive MPOWER policies worldwide to protect the health of children, while developing and evaluating new and ongoing tobacco control policy initiatives around the world.

In a previous systematic review,^15^ we partly addressed this gap in the literature by synthesising available evidence on the effect of smoke-free legislation (ie, “P” in MPOWER, for ”Protect people from tobacco smoke”) on perinatal and child health. By combining data from 11 studies, we found smoke-free legislation to be associated with substantial reductions in preterm birth and hospital admissions for asthma among children. Studies have since addressed various knowledge gaps identified in our review, including assessments of the effect of smoke-free legislation on respiratory tract infections and on general practitioner (GP) consultations.^16^, ^17^, ^18^, ^19^ The increased number of studies now available was also anticipated to allow investigation of another knowledge gap: exploration of a potential dose–response association between the comprehensiveness of smoke-free laws and their effect on child health. In addition to addressing this association, we sought to substantially broaden the focus of our systematic review by systematically evaluating the early-life health effect of the entire range of MPOWER measures. This analysis has implications for the Sustainable Development Goal 3 (SDG 3) aims to strengthen FCTC implementation and reduce child mortality. As such, findings from this study can guide policy making for prioritisation of the most effective tobacco control policies to protect child health, especially in parts of the world where MPOWER implementation is lagging behind, while identifying the key remaining knowledge gaps that need to be addressed.

## Methods

### Search strategy and selection criteria

This systematic review and meta-analysis was done according to a peer-reviewed protocol that is published^20^ and registered with PROSPERO (CRD42015023448). We followed the PRISMA checklist when reporting our findings.^21^ Ethical approval was not required for this study.

Studies were eligible for inclusion if they investigated the association between one or more MPOWER tobacco control policies and health outcomes among fetuses, neonates, or children (ie, the majority of the study population aged <12 years).

We searched for published studies in the following databases: Cochrane Central Register of Controlled Trials (CENTRAL), MEDLINE, Embase, PsycINFO, Cumulative Index to Nursing and Allied Health Literature (CINAHL), WHO Global Health Library (in addition to MEDLINE, covering African Index Medicus [AIM], LILACS, Index Medicus for the Eastern Mediterranean Region [IMEMR], Index Medicus for South-East Asia Region [IMSEAR], Western Pacific Region Index Medicus [WPRIM], WHO Library Database [WHOLIS], and Scientific Electronic Library Online [SciELO]), IndMED, ISI Web of Science, KoreaMed, EconLit, Paediatric Economic Database Evaluation (PEDE), Google Scholar, and the ProQuest database of PhD dissertations. We searched the WHO International Clinical Trials Registry Platform (ICTRP) for unpublished studies.

The appendix (p 1) contains an overview of the search strategies for each database. We did not apply any language restrictions, and searched the full time period available for each database. Searches were updated on June 22, 2017. To identify any additional relevant studies, we hand-searched reference lists of, and citations to, included studies and relevant review papers, and consulted experts in the field (appendix p 2).

We focused on studies that evaluated governmental public health interventions that could be classified according to the MPOWER acronym (panel), with the exception of “M” since “Monitoring tobacco use and prevention policies” itself was not expected to affect health outcomes. We followed the methodological approach recommended by the Cochrane Effective Practice and Organization of Care (EPOC) group to select studies with the most robust designs for our primary analyses: randomised controlled trials (including cluster randomised controlled trials), controlled clinical trials (including cluster controlled clinical trials), interrupted time series studies (including difference-in-difference designs, which were categorised as controlled interrupted time series studies),^22^ and controlled before-and-after studies. To assess the robustness of our findings, we also included non-EPOC study designs in sensitivity analyses: uncontrolled before-and-after studies, prospective or retrospective cohort studies, and case-control and nested case-control studies. Primary and secondary outcomes were selected on the basis of their established associations with maternal smoking during pregnancy and prenatal or childhood second-hand smoke exposure,^23^, ^24^ and their relative contributions to the global burden of adverse child health.^1^, ^25^ Our primary outcomes of interest were perinatal mortality, preterm birth, asthma exacerbations requiring hospital attendance, and respiratory tract infections requiring hospital attendance. Secondary outcomes of interest were stillbirth, early neonatal mortality, neonatal mortality, late neonatal mortality, post-neonatal mortality, infant mortality, child mortality, extremely low birthweight, very low birthweight, low birthweight, birthweight (continuous scale), very small for gestational age, small for gestational age, extremely preterm birth, very preterm birth, gestational age (continuous scale), congenital anomalies, asthma, wheezing, respiratory tract infections, upper respiratory tract infections, lower respiratory tract infections, otitis media with effusion, and chronic cough. Studies were excluded if they only measured smoking prevalence, smoking behaviour, second-hand smoke exposure, surrogate outcomes, or economic outcomes. Studies that reported outcomes for both adults and children were included if paediatric subgroup data were available.

### Data analysis

Two reviewers (TF and AK) independently assessed all search results by title and abstract, and by full text for potential eligible studies identified. Any disagreements were resolved through joint discussion or via an adjudicator (JVB).

Relevant data were extracted with a customised data extraction form (appendix, pp 3–5). Study authors were contacted for clarification where necessary and to obtain relevant data that were missing from the reports.

A risk-of-bias assessment form was created on the basis of EPOC criteria for interrupted time series and controlled before-and-after studies.^26^ The Effective Public Health Practice Project (EPHPP) tool was adapted to assess the risk of bias of observational studies.^27^ Two reviewers (TF and AK) independently extracted data and assessed risk of bias, with disagreements resolved through discussion or arbitration (JVB).

Point estimates and corresponding 95% CIs for effect sizes or association measures were extracted. For dichotomous outcomes, risk ratios (RRs) were extracted. Where RRs were not available, we calculated RRs from odds ratios (ORs) using the following formula, where PEER is the patient-expected event rate in the control group:

RR=OR/(1-PEER)+(PEER × OR)

When PEER was not available in interrupted time series studies we used the overall event rate across the study population as an approximation. For outcomes that could occur more than once (eg, hospital attendances for asthma and respiratory tract infections), we used incidence rate ratios (IRRs).

Aggregated effect estimates were calculated to assess the association between each tobacco control policy and individual health outcomes, where feasible. Relative risk differences were extracted or calculated from absolute risk differences and were pooled in random-effects meta-analyses given anticipated heterogeneity. Step changes (ie, immediate risk changes) following introduction of an intervention were pooled in separate analyses from slope changes (ie, gradual risk changes). Heterogeneity was assessed by the *I*^2^ statistic. For the meta-analyses, we selected the effect estimate of the most comprehensive intervention within each MPOWER category from each study. In case of overlapping populations between studies, we selected one study according to the following hierarchy: the lowest risk of bias, the most comprehensive intervention, or the largest study population. We also extracted data on changes in smoking behaviour and second-hand smoke exposure if reported. The comprehensiveness of smoke-free legislation was assessed by counting the number of locations that were made completely smoke-free, out of eight prespecified options as suggested by WHO.^2^ Policies that were completely smoke-free in all eight locations were considered to be comprehensive.

We did sensitivity analyses to explore the robustness of our findings by reanalysing the data for the primary outcomes with the addition of non-EPOC studies, and by restricting analyses to studies with low risk of bias and moderate risk of bias. Where possible, we did subgroup analyses according to the comprehensiveness of each intervention. Where possible, the effect of each intervention was reported according to socioeconomic status, alongside its overall effect.

We assessed risk of bias across studies using funnel plots when ten or more studies were included in a meta-analysis.

### Role of the funding source

The funders of the study had no role in study design, data collection, data analysis, data interpretation, or writing of the report. The corresponding author had full access to all the data in the study and had final responsibility for the decision to submit for publication.

## Results

**Figure 1:**
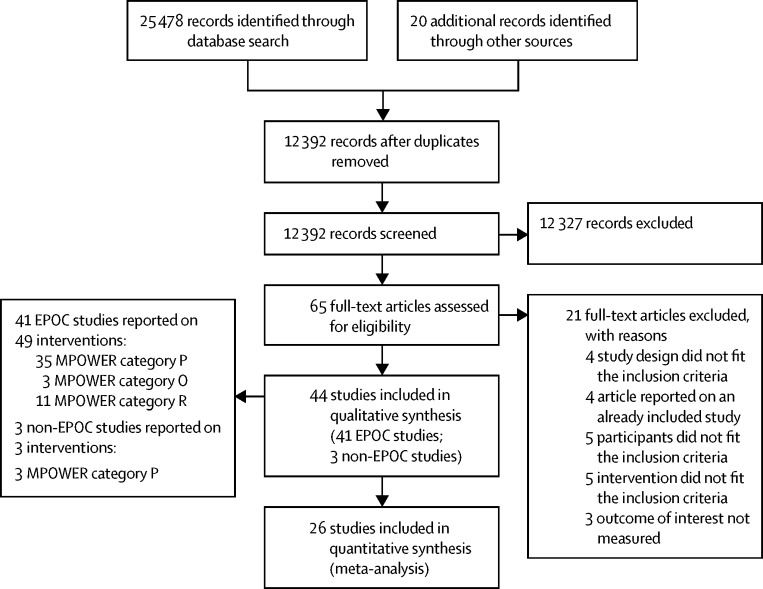
PRISMA flow diagram EPOC= Effective Practice and Organization of Care (a Cochrane Review Group). MPOWER=WHO's recommended tobacco control policies (see panel).

We identified 25 478 citations from bibliographic databases and an additional 20 from other sources. After removal of duplicates, 12 392 unique citations were screened by title and abstract, and 65 full texts were sourced. Of these, 41 EPOC studies^16^, ^17^, ^18^, ^19^, ^28^, ^29^, ^30^, ^31^, ^32^, ^33^, ^34^, ^35^, ^36^, ^37^, ^38^, ^39^, ^40^, ^41^, ^42^, ^43^, ^44^, ^45^, ^46^, ^47^, ^48^, ^49^, ^50^, ^51^, ^52^, ^53^, ^54^, ^55^, ^56^, ^57^, ^58^, ^59^, ^60^, ^61^, ^62^, ^63^, ^64^ and three non-EPOC studies^65^, ^66^, ^67^ fit the inclusion criteria (figure 1; appendix, pp 6, 7). The EPOC studies included data from more than 57 million births, and from 4·6 million GP diagnoses and 2·7 million hospital admissions for respiratory conditions.

The appendix (pp 8–20) details the main characteristics of the EPOC studies. Among these, 26 were interrupted time series studies,^16^, ^17^, ^18^, ^19^, ^31^, ^32^, ^37^, ^38^, ^40^, ^42^, ^43^, ^45^, ^46^, ^48^, ^50^, ^51^, ^52^, ^54^, ^55^, ^57^, ^58^, ^59^, ^61^, ^62^, ^63^, ^64^ 14 were controlled interrupted time series studies,^28^, ^29^, ^33^, ^34^, ^35^, ^36^, ^39^, ^41^, ^44^, ^47^, ^49^, ^53^, ^56^, ^60^ and one had a regression discontinuity design,^30^ a quasi-experimental design bearing close resemblance to interrupted time series methodology.^68^ The three non-EPOC studies were uncontrolled before-and-after studies (appendix, p 21).^65^, ^66^, ^67^ Model characteristics of individual studies can be found in the appendix (pp 22–31). The EPOC studies were done in 14 countries across North America (24 studies)^18^, ^28^, ^29^, ^31^, ^33^, ^35^, ^36^, ^38^, ^39^, ^41^, ^42^, ^43^, ^45^, ^46^, ^47^, ^49^, ^50^, ^53^, ^54^, ^56^, ^57^, ^59^, ^60^, ^61^ and Europe (16 studies),^16^, ^17^, ^30^, ^32^, ^34^, ^37^, ^40^, ^44^, ^48^, ^51^, ^52^, ^55^, ^58^, ^62^, ^63^, ^64^ with one study from Hong Kong, China.^19^ Several US studies assessed the same outcomes in partially overlapping study populations.^18^, ^29^, ^31^, ^36^, ^38^, ^43^, ^45^, ^46^, ^49^, ^53^, ^56^, ^59^, ^61^

Risk of bias of individual studies is reported in detail in the appendix (pp 32, 33). For the EPOC studies, risk of bias was low in 23 studies,^16^, ^17^, ^18^, ^28^, ^30^, ^31^, ^32^, ^33^, ^36^, ^37^, ^39^, ^40^, ^44^, ^45^, ^46^, ^47^, ^48^, ^51^, ^52^, ^53^, ^58^, ^63^, ^64^ moderate in 16,^19^, ^34^, ^35^, ^41^, ^42^, ^43^, ^49^, ^50^, ^54^, ^55^, ^56^, ^57^, ^59^, ^60^, ^61^, ^62^ and high in two.^29^, ^38^ For the non-EPOC studies, risk of bias was high for two studies^66^, ^67^ and unclear for one.^65^

28 studies assessed the association between smoke-free legislation and one or more primary outcomes (ie, perinatal mortality, preterm birth, asthma exacerbations requiring hospital attendance, and respiratory tract infections requiring hospital attendance), five assessed the association between tobacco taxation and primary outcomes, and two assessed the association between policies providing smoking cessation services and our primary outcomes (Table 1, Table 2, Table 3); four studies assessed a combination of these interventions, and ten studies only assessed secondary outcomes. A meta-analysis was only possible for studies on smoke-free legislation because studies on tax increases and smoking cessation services had variable outcome reporting and overlapping study populations.

**Table 1:** Association between implementation of smoke-free legislation and primary outcomes

	**Details of intervention**	**Population at risk (n)**	**Events (n)**	**Slope before intervention (% change in events per year)**	**Direct change in events (step change, %; 95% CI)**	**Sustained change in events per year (slope change, %; 95% CI)**	**Summary of findings**
**Perinatal mortality**							
Peelen (2016)^58^*	First smoke-free law: workplaces and public transport except for restaurants and bars† (allowing designated smoking areas) Second smoke-free law: expansion of first smoke-free law to include restaurants and bars‡(allowing designated smoking areas)	1 980 727	13 027	NA because of non-linear time trend	First smoke-free law: −1·99% (−8·95 to 5·96) Second smoke-free law: −5·96% (−12·93 to 1·99)	NA	National smoke-free workplaces and public transport, and smoke-free restaurants and bars, were not associated with significant changes in perinatal mortality
**Preterm birth**							
Bakolis (2016)^30^	Public places and workplaces (including restaurants and bars)	1 800 906	126 527	NR	Analysis of a 1, 2, 3, or 5 month time window around the intervention cutoff date (July 1, 2007): ±1 month, −4·67% (−16·00 to −0·93); ±2 months, −8·42% (−15·05 to −1·86); ±3 months, −5·60% (−10·31 to −0·93); ±5 months, −3·73% (−7·48 to −0·93)	NA	National comprehensive smoke-free legislation was associated with an immediate 4–9% decrease in preterm births
Bartholomew (2016)^31^	Comprehensive (workplaces, restaurants, and bars) Restrictive (workplaces and restaurants, no restriction in bars) Moderate (workplaces, partial restriction in restaurants, and no restriction in bars) Limited (partial restriction in workplaces, any restriction in restaurants, and no restriction in bars)	293 715	32 250	NR	Comprehensive: −0·015%§ (−0·022 to −0·008) Restrictive: 0·003%§ (−0·005 to 0·011) Moderate: 0·004%§ (−0·002 to 0·010) Limited: 0·001%§ (−0·006 to 0·007)	NA	County-wide, comprehensive smoke-free legislation was associated with a 0·015 percentage point decrease in preterm births, whereas less restrictive laws were not associated with changes in incidence of preterm births
Bharadwaj (2014)^34^	Restaurants and bars (in addition to existing smoke-free laws in public places and workplaces)	822 (intervention group), 3185 (control group)	46 (intervention group), 189 (control group)	NR	−2·55%§ (−5·52 to 0·42)	NA	National smoke-free restaurants and bars were not associated with significant changes in preterm births among women working in restaurants and bars
Cox (2013)^37^	Public places and workplaces (excluding catering industry); restaurants (in addition to existing smoke-free laws in public places and workplaces); and bars serving food (in addition to existing smoke-free laws in public places and workplaces, including restaurants)	606 877	36 663	NR	Public places and workplaces: single smoke-free law¶, −0·59% (−2·63 to 1·49); final model‖, no significant changes Restaurants (in addition to public places and workplaces): single smoke-free law¶, −2·28% (−4·73 to −0·15); final model‖, −3·18% (−5·38 to −0·94) Bars serving food (in addition to restaurants and public places and workplaces): single smoke-free law¶, −1·24% (−3·05 to 0·60); final model‖, no significant changes	Public places and workplaces: single smoke-free law†, −1·95% (−3·50 to −0·37); final model‡, no significant changes Restaurants (in addition to public places and workplaces): single smoke-free law†, −1·42% (−2·87 to 0·05); final model‡, no significant changes Bars serving food (in addition to restaurants and public places and workplaces): single smoke-free law†, −2·10% (−4·82 to 0·69); final model‡, −3·50% (−6·35 to −0·57)	National smoke-free public places and workplaces were not associated with significant changes in preterm births; expansion of national smoke-free legislation to include restaurants was associated with an immediate 3·2% reduction in preterm births; and expansion of national smoke-free legislation to include bars was associated with a gradual 4% per year decrease in preterm births
Hade (2011)^43^	Public places and workplaces (including restaurants and bars)	583 530	NR	NR	No significant changes**	No significant changes**	State-wide, smoke-free public places and workplaces were not associated with significant changes in preterm birth
Hajdu (2017)^44^	Public places and workplaces (including restaurants and bars)	18 755	NR	NR	–1·9%§ (–4·3 to 0·5)	NA	National smoke-free legislation was not associated with significant changes in preterm birth among female restaurant and bar workers compared with women working in places other than restaurants and bars
Hankins (2016)^45^	Workplaces, restaurants, and bars	NR	NR	NR	Workplaces: 0·07%§ (−0·11 to 0·25) Restaurants: 0·09%§ (−0·13 to 0·31) Bars: −0·29%§ (−0·49 to −0·09)	NA	State-wide or county smoke-free workplaces and restaurants were not associated with significant changes in preterm births; state-wide or county smoke-free bars were associated with an immediate 0·3 percentage point decrease in preterm births
Hawkins (2014)^46^	100% smoke-free workplaces and restaurants	16 198 654	1 555 071	NR	0·72%§ (−0·11 to 1·55)	NA	State-wide smoke-free workplaces and restaurants were not associated with significant changes in preterm births
Mackay (2012)^52^	Public places and workplaces (including restaurants and bars)	709 756	41 998	NR	Crude: −11·07% (−15·15 to −6·79) Adjusted: −11·72% (−15·87 to −7·35)	Crude: 2·28% (−0·03 to 4·66) Adjusted: 3·83% (1·42 to 6·30)	National smoke-free public places and workplaces were associated with an immediate 12% decrease in preterm births, and a subsequent gradual 4% increase per year
Markowitz (2013)^53^	Workplaces with complete smoke-free law Workplaces with smoking restrictions (requiring designated smoking areas) Restaurants with complete smoke-free laws Restaurants with smoking restrictions (requiring designated smoking areas)	Maternal age <20 years: 54 132Maternal age 20–24 years: 101 723Maternal age 25–34 years: 183 763Maternal age >34 years: 53 109	Maternal age <20 years: 5413Maternal age 20–24 years: 7120Maternal age 25–34 years: 11 026Maternal age >34 years: 3718	NR	Workplaces with complete smoke-free laws: NRWorkplaces with smoking restrictions: NRRestaurants with complete smoke-free laws: maternal age <20 years, 0·7%§ (−3·5 to 4·9);20–24 years, −0·2%§ (−1·5 to 1·1); 25–34 years, −0·3%§ (−0·8 to 0·2); >34 years, −0·6%§ (−1·9 to 0·7) Restaurants with smoking restrictions: maternal age <20 years, −0·6%§ (−3·8 to 2·6); 20–24 years, −0·1%§ (−1·1 to 0·9); 25–34 years, −0·8%§ (−1·2 to −0·4); >34 years, −0·3%§ (−1·7 to 1·1)	NA	State-wide complete smoke-free laws were not associated with significant changes in preterm births, but state-wide restaurant smoking restrictions were associated with a 0·8 percentage point decrease in preterm births among women aged 25–34 years
McKinnon (2015)^54^	Public places and workplaces (including restaurants and bars)	470 199	19 321	NR	Crude: −6% (−10 to −1) Adjusted: −5% (−10 to 0)	NA	State-wide smoke-free legislation was associated with a 5% decrease in preterm births 9 months after its implementation
Page (2012)^56^	Public places and workplaces (including restaurants and bars)	6717 (intervention group), 32 293 (control group)	515 (intervention group), 2767 (control group)	NR	Crude: −20·6% (−34·7 to −3·4) Adjusted: −23·1% (−40·1 to −1·3)	NA	City-wide smoke-free public places and workplaces were associated with a 23% decrease in preterm births
Peelen (2016)^58^*	First smoke-free law†: workplaces and public transport except for restaurants and bars (allowing designated smoking areas) Second smoke-free law‡: expansion of first smoke-free law to include restaurants and bars (allowing designated smoking areas)	1 972 163	116 043	NA because of non-linear time trend	First smoke-free law: 0·94% (−1·89 to 3·77) Second smoke-free law: −0·94% (−3·78 to 2·83)	NA	National smoke-free workplaces and public transport, and smoke-free restaurants and bars, were not associated with significant changes in preterm births
Simón (2017)^62^	First smoke-free law: complete smoke-free workplaces and partial smoke-free restaurants and bars Second smoke-free law: public places and workplaces (including restaurants and bars)	5 302 374	416 595	NR	First smoke-free law: 4·6% (2·9 to 6·2) Second smoke-free law: −4·5% (–6·1 to −2·9)	NA	National partial smoke-free legislation was associated with a 5% increase in preterm births; the subsequent national comprehensive smoke-free legislation was associated with a 5% decrease in preterm births
Vicedo-Cabrera (2016)^63^	Public places and workplaces (including restaurants and bars), with several exceptions in the hospitality sector††	446 492	24 482	NR	−3·56% (−9·29 to 2·53)	NA	Federal smoke-free legislation was not associated with a significant change in preterm births
**Asthma exacerbations requiring hospital attendance**							
Ciaccio (2016)^36^	Public places and workplaces (including restaurants and bars)	13 246 809	335 588	NR	–17% (–18 to −15)	NA	State or local smoke-free legislation was associated with an immediate 17% decrease in emergency department visits for asthma
Croghan (2015)^38^	Public places and workplaces (including restaurants and bars)	NR	1531	1·1% (0·2 to 2·0)	−24·9% (−40·5 to −5·3)	−1·5% (−2·9 to −0·1)	National smoke-free legislation was associated with an immediate 25% decrease in emergency department visits for children with asthma, and a subsequent gradual 1·5% decrease per year
Galán (2017)^40^	First smoke-free law: complete smoke-free workplaces and partial smoke-free restaurants and bars Second smoke-free law: public places and workplaces (including restaurants and bars)	NR	NR	NR	First smoke-free law: 25·0% (–2·6 to 60·4) Second smoke-free law: −11·0% (–28·6 to 11·1)	NA	Partial and comprehensive national smoke-free legislation were not associated with significant immediate changes in asthma-related hospital admissions via emergency departments
Gaudreau (2013)^42^	Public places and workplaces (including restaurants and bars), allowing designated smoking areas	NR	3050	NR	11% (−37 to 95)	0% (−2 to 2)	Provincial smoke-free public places and workplaces were not associated with significant changes in hospital admissions for paediatric asthma
Hawkins (2016)^18^	State or local 100% smoke-free workplaces or restaurants, or both	NR	128 807	NR	State: −3% (−8 to 2) Local: 2% (−6 to 11)	NA	State or local smoke-free workplaces or restaurants were not associated with significant changes in emergency department visits for paediatric asthma
Landers (2014)^49^	100% smoke-free workplaces, restaurants, and bars‡‡	NR	NR	Mean rate across all states and years: 9·02 per 10 000 per quarter (SD 9·66; range 0·00–144·47)	Any state law: 0·12%§ (−0·38 to 0·62) Any county law: −1·32%§ (−2·64 to 0·00) Interaction term of state law and county law: 0·51%§ (−1·04 to 2·06)	NA	County-level smoke-free laws were associated with a one percentage point decrease in discharge rates among children admitted for asthma; state smoke-free laws were not associated with significant changes in discharge rates among children admitted for asthma, besides the effect of county laws
Mackay (2010)^51^	Public places and workplaces (including restaurants and bars)	NR	21 415	4·4% (3·3 to 5·5)	NA	−19·5% (−22·4 to −16·5)	National smoke-free public places and workplaces were associated with a gradual 20% decrease per year in paediatric emergency asthma admissions
Millett (2013)^55^	Public places and workplaces (including restaurants and bars)	NR	217 381	2·2% (2 to 3)	−8·9% (−11 to −7)	−3·4% (−4 to −2)	National smoke-free public places and workplaces were associated with an immediate 9% decrease in emergency admissions to hospital for paediatric asthma, and a subsequent gradual 3% decrease per year
Rayens (2008)^59^	Most businesses open to the public (including restaurants and bars)§§	395 116	5322	12·7%	−18·0% (−29·0 to −4·0)	NA	The county-wide smoke-free law in most public places was associated with an 18% decrease in emergency department visits for paediatric asthma
Shetty (2011)^61^	All workplaces except restaurants and bars: 100% smoke-free Any smoke-free workplace, restaurant, or bar law	NR	NR	NR	100% smoke-free workplaces: 14·6% (3·7 to 25·5) Any smoke-free law: 9·0 (−1 to 19·1)	NA	State-wide or region-wide 100% smoke-free workplaces were associated with a 15% increase in hospital admissions for children with asthma; there was no evidence for an association between any state-wide or region-wide smoke-free legislation and asthma admissions
**RTI admissions (upper and lower)**							
Been, Millett (2015)^16^	Public places and workplace (including restaurants and bars)	NR	1 651 675	NR	−3·5% (−4·7 to −2·3)	−0·5% (−0·9 to −0·1)	National smoke-free legislation was associated with an immediate 4% reduction and an additional 0·5% annual reduction in childhood acute RTI hospital admissions
Vicedo-Cabrera (2017)^64^	Public places and workplaces (including restaurants and bars), with several exceptions in the hospitality sector††	NR	29 345	NR	2·7% (–9·7 to 16·7)	NA	Federal smoke-free legislation was not associated with a significant change in RTI hospital admissions
**Upper RTI admissions**							
Been, Millett (2015)^16^	Public places and workplaces (including restaurants and bars)	NR	979 370	NR	1·9% (0·5 to 3·2)	−1·9 (−2·3 to −1·5)	National smoke-free legislation was associated with an initial immediate 2% increase in childhood upper RTI hospital admissions, followed by a gradual decrease of 2% per year
Hawkins (2016)^18^	State or local 100% smoke-free workplaces or restaurants, or both	NR	410 686	NR	State: −2% (−6 to 2) Local: 6% (−2 to 14)	NA	State or local smoke-free workplaces or restaurants were not associated with significant changes in emergency department visits for upper RTIs
**Lower RTI admissions**							
Been, Millett (2015)^16^	Public places and workplaces (including restaurants and bars)	NR	672 305	NR	−13·8% (−15·6 to −12·0)	0·2% (−0·6 to 0·9)	National smoke-free legislation was associated with an immediate 14% reduction in childhood lower RTI hospital admissions
Hawkins (2016)^18^	State or local 100% smoke-free workplaces or restaurants, or both	NR	139 239	NR	State: −8% (−13 to −4) Local: 3% (−6 to 12)	NA	State-wide smoke-free workplaces or restaurants were associated with an 8% decrease in emergency department visits for lower RTIs
Lee (2016)^19^	Public places and workplaces (including restaurants)	691 480	75 870	NR	−33·5% (−36·4 to −30·5)	−13·9% (−16·0 to −11·7)	Comprehensive smoke-free legislation was associated with an immediate 34% reduction in hospital admissions for childhood lower RTIs, and a subsequent gradual decrease of 14% per year

NA=not applicable. NR=not reported. RTI=respiratory tract infection.

* Both smoke-free laws were accompanied by a tobacco tax increase and mass-media campaign.

† Exceptions to this smoke-free law were: hotels, bars and restaurants, sports, arts and culture venues, amusement arcades, tobacconist shops, international passenger transport systems, private spaces, open air, and designated areas for smoking within each facility.

‡ The smoke-free law now included hospitality venues: hotels, bars and restaurants, sports, art and culture venues, amusement arcades, tobacconist shops, and international passenger transport systems. Designated smoking areas within each facility were still allowed.

§ Percentage point change.

¶ The single smoke-free law model includes either the step or slope change of a single smoke-free law into the model.

‖ The final was obtained by including all three step changes and all three slope changes in one model and removing the least significant factors one at a time.

** No association measures were reported.

†† Authorised smoking in establishments smaller than 80 m^2^ and designated smoking areas in larger establishments.

‡‡ Different states passed different 100% smoke-free laws: workplaces, restaurants, and bars (eight states); restaurants and bars (two states); workplaces and restaurants (one state); and workplaces (one state).

§§ Including, but not limited to restaurants, bars, bowling alleys, bingo halls, convenience stores, laundromats, and other business open to the public.

**Table 2:** Association between implementation of smoking cessation services and primary outcomes

	**Details of intervention**	**Population at risk (n)**	**Events (n)**	**Slope before intervention (% change in events per year)**	**Direct change in events (step change, %; 95% CI)**	**Sustained change in events per year (slope change, %; 95% CI)**	**Summary of findings**
**Preterm birth**							
Jarlenski (2014)^47^	State adoption of one of two optional Medicaid enrolment policies, allowing more low-income pregnant women to receive prenatal care, including smoking cessation services (presumptive eligibility and the unborn child option)*	24 544	NR	NR	Overall: −1·4%§ (−4·7 to 2·0)Comprehensive: −2·2%§ (−5·9 to 1·5)Non-comprehensive: 1·3%§ (−2·4 to 5·1)	NA	Neither optional Medicaid enrolment policy was associated with significant changes in preterm birth
**Asthma exacerbations requiring hospital attendance**							
Hawkins (2016)^18^	Health reform legislation that provided counselling for smoking cessation and tobacco cessation treatment to Medicaid recipients	NR	112 808	NR	2% (−4 to 8)	NA	The state-wide health reform legislation in MA, USA, was not associated with significant changes in emergency department visits for asthma
**Upper RTI admissions**							
Hawkins (2016)^18^	Health reform legislation that provided counselling for smoking cessation and tobacco cessation treatment to Medicaid recipients	NR	337 628	NR	−6% (−10 to −1)	NA	The state-wide health reform legislation in MA, USA, was associated with a 6% decrease in emergency department visits for upper RTIs
**Lower RTI admissions**							
Hawkins (2016)^18^	Health reform legislation that provided counselling for smoking cessation and tobacco cessation treatment to Medicaid recipients	NR	113 137	NR	0% (−6 to 6)	NA	The state-wide health reform legislation in MA, USA, was not associated with significant changes in emergency department visits for lower RTIs

NR=not reported. NA=not applicable. RTI=respiratory tract infection.

* Presumptive eligibility: low-income pregnant women are presumed to be eligible for Medicaid, so they can receive care (including smoking cessation services) while their Medicaid applications are still pending. The unborn-child option: the state can consider a fetus a “targeted low-income child”, allowing coverage of prenatal care (including smoking cessation services) and delivery to low-income pregnant women, even if they cannot provide documentation of citizenship or residency.

**Table 3:** Association between implementation of tobacco taxation and primary outcomes

	**Details of intervention**	**Population at risk (n)**	**Events (n)**	**Slope before intervention (% change in events per year)**	**Direct change in events (step change, %; 95% CI)**	**Sustained change in events per year (slope change, %; 95% CI)**	**Summary of findings**
**Preterm birth**							
Hawkins (2014)^46^	Effect of cigarette excise tax increase (in USD$; December 2010 rates) on mothers, by years of maternal education	9 981 855	NR	NR	White mothers: 0–11 years, −0·07%§ (−0·11 to −0·02); 12 years, −0·02%§ (−0·05 to 0·01); 13–15 years, −0·01%§ (−0·03 to 0·00); ≥16 years, −0·00%§ (−0·01 to 0·01) per USD$ increase in tax	NA	Cigarette taxes were associated with a decrease in preterm birth among white mothers with the least amount of education
Hawkins (2014)^46^	Effect of cigarette excise tax increase (in USD$; December 2010 rates) on mothers, by years of maternal education	2 722 846	NR	NR	Black mothers: 0–11 years, −0·08%§ (−0·14 to −0·03); 12 years, −0·04%§ (−0·07 to −0·01); 13–15 years, −0·03%§ (−0·05 to −0·01); ≥16 years, −0·01%§ (−0·01 to −0·00) per USD$ increase in tax	NA	Cigarette taxes were associated with a decrease in preterm births among black mothers with any level of education; among black mothers, there was a gradient across maternal education levels, with the largest decreases among mothers with the least amount of education
Hawkins (2014)^46^	Effect of cigarette excise tax increase (in USD$; December 2010 rates) on mothers, by years of maternal education	2 444 673	NR	NR	Hispanic mothers: 0–11 years, 0·01%§ (−0·00 to 0·02); 12 years, −0·00%§ (−0·01 to 0·00); 13–15 years, −0·01%§ (−0·02 to 0·00); ≥16 years, −0·00%§ (−0·00 to 0·00) per USD$ increase in tax	NA	Cigarette taxes were not associated with significant changes in preterm births among Hispanic mothers with any level of education
Hawkins (2014)^46^	Effect of cigarette excise tax increase (in USD$; December 2010 rates) on mothers, by years of maternal education	804 447	NR	NR	Asian/Pacific Islander mothers: 0–11 years, 0·01%§ (−0·01 to 0·04); 12 years, −0·01%§ (−0·01 to 0·00); 13–15 years, −0·00%§ (−0·01 to 0·01); ≥16 years, 0·00%§ (−0·00 to 0·00) per USD$ increase in tax	NA	Cigarette taxes were not associated with significant changes in preterm births among Asian/Pacific Islander mothers with any level of education
Hawkins (2014)^46^	Effect of cigarette excise tax increase (in USD$; December, 2010, rates) on mothers, by years of maternal education	244 823	NR	NR	Native American/Alaska Native mothers: 0–11 years, −0·02%§ (−0·08 to 0·04); 12 years, 0·01%§ (−0·02 to 0·03);13–15 years, 0·00%§ (−0·03 to 0·03); ≥16 years, −0·01%§ (−0·02 to 0·01) per USD$ increase in tax	NA	Cigarette taxes were not associated with significant changes in preterm births among Native American/Alaska Native mothers with any level of education
Markowitz (2013)^53^	Cigarette excise tax increase (in 2008 USD$)Cigarette price increase (in 2008 USD$)	Maternal age <20 years: 54 132Maternal age 20–24 years: 101 723Maternal age 25–34 years: 183 763Maternal age >34 years: 53 109	Maternal age <20 years: 5413Maternal age 20–24 years: 7120Maternal age 25–34 years: 11 026Maternal age >34 years: 3718	NR	Cigarette excise tax: maternal age <20 years, −2·0%§ (−4·0 to 0·0) per USD$ increase in tax;maternal age 20–24 years, −0·7%§ (−1·4 to −0·0) per USD$ increase in tax; maternal age 25–34 years, −0·2%§ (−1·0 to 0·6) per USD$ increase in tax; maternal age >34 years, −1·0%§ (−1·9 to −0·1) per USD$ increase in taxCigarette price: NR	NA	State-wide increases in cigarette excise tax were associated with a 0·7 percentage point decrease in preterm births among women aged 20–24 years, and a 1·0 percentage point decrease among women aged >34 years
**Asthma exacerbations requiring hospital attendance**							
Hawkins (2016)^18^	Cigarette excise tax increase in USD$	NR	128 807	NR	−5% (−11 to 1) per USD$ increase in tax	NA	State-wide increase in cigarette excise tax was not associated with significant changes in emergency department visits for paediatric asthma
Landers (2014)^49^	Cigarette excise tax increase in USD$	NR	NR	Mean rate across all states and years: 9·02 per 10 000 (SD 9·66; range 0·00– 144·47)	−0·53%§ (−0·99 to −0·06) per USD$ increase in tax	NA	State-wide increase in cigarette excise tax was associated with a 0·5 percentage point decrease in asthma discharge rates
Ma (2013)^50^	USD$0·69 cigarette excise tax increase;USD$0·35 cigarette excise tax increase	28 498 070	702 771	0·04	USD$0·69 cigarette excise tax increase: −11·01% (−24·71 to 2·77);USD$0·35 cigarette excise tax increase: −22·02% (−33·46 to −9·95)	USD$0·69 cigarette excise tax increase: 4·88% (1·29 to 8·59)USD$0·35 cigarette excise tax increase: −4·72% (−8·01 to −1·44)	The first cigarette excise tax increase (USD$0·69) was not associated with significant immediate changes, but was associated with a significant, gradual increase in asthma-related hospital admissions of 0·5% per year; the second cigarette excise tax increase (USD$0·35) was associated with both a 22% immediate decrease as well as a gradual 5% decrease in asthma-related hospital admissions per year
**Upper RTI admissions**							
Hawkins (2016)^18^	Cigarette excise tax increase in USD$	NR	410 686	NR	−2% (−6% to 2%) per USD$ increase in tax	NA	State-wide increase in cigarette excise tax was not associated with significant changes in emergency department visits for upper RTIs
**Lower RTI admissions**							
Hawkins (2016)^18^	Cigarette excise tax increase in USD$	NR	139 239	NR	−9% (−16 to −2) per USD$ increase in tax	NA	State-wide increase in cigarette excise tax was associated with a 9% decrease in emergency department visits for lower RTIs

NA=not applicable. NR=not reported. RTI=respiratory tract infection.

A national study from the Netherlands, comprising 1 980 727 births, found no change in perinatal mortality following a law to prohibit smoking in workplaces and on public transport, or following expansion of the law to include restaurants and bars.^58^ In a study from England, comprising 10 291 113 births, comprehensive smoke-free legislation in public places and workplaces (including restaurants and bars) was associated with a reduction in stillbirths (–7·8%; 95% CI −18·0 to −3·5) and neonatal deaths (–7·6%; −11·7 to −3·4).^32^ The overall effect on perinatal mortality (ie, stillbirths and early neonatal deaths combined) was not reported in this study. Therefore, no meta-analysis was possible with these two studies.

**Figure 2:**
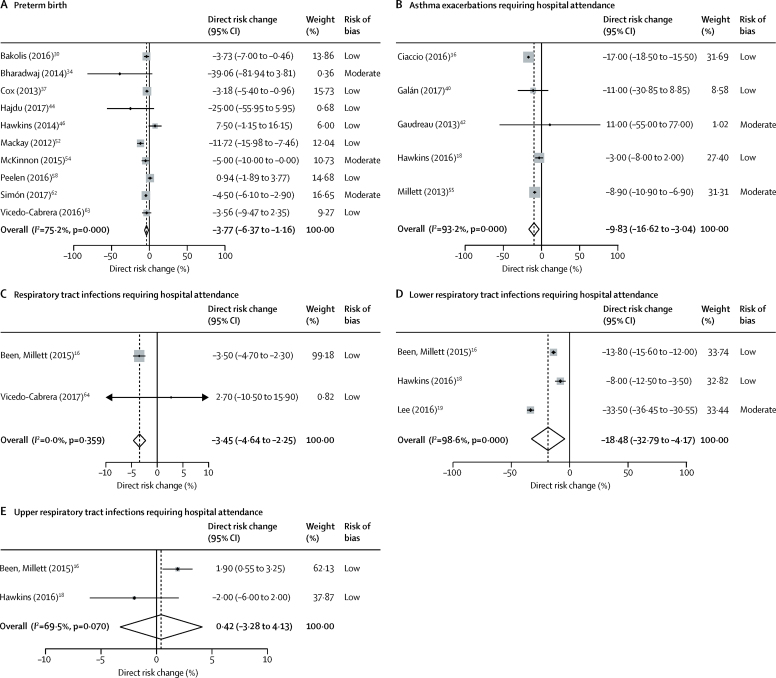
Meta-analysis of immediate changes in primary outcomes after implementation of smoke-free legislation (A) Preterm birth. (B) Asthma exacerbations requiring hospital attendance. (C) Respiratory tract infections requiring hospital attendance. (D) Lower respiratory tract infections requiring hospital attendance. (E) Upper respiratory tract infections requiring hospital attendance.

**Figure 3:**
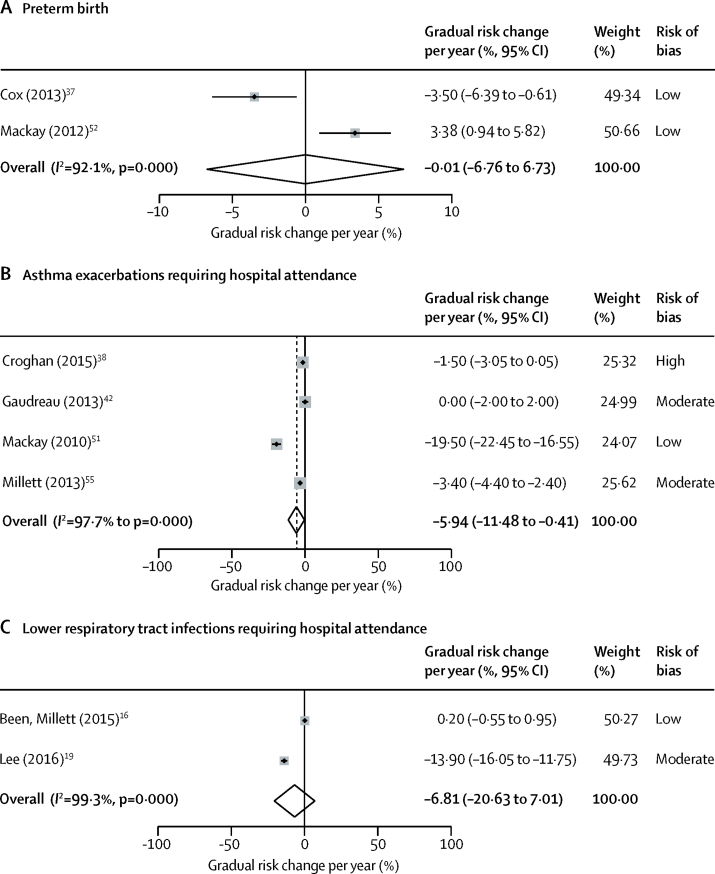
Meta-analysis of gradual changes in primary outcomes after implementation of smoke-free legislation (A) Preterm birth. (B) Asthma exacerbations requiring hospital attendance. (C) Lower respiratory tract infections requiring hospital attendance.

15 studies investigated the association between smoke-free legislation and preterm births.^30^, ^31^, ^34^, ^37^, ^43^, ^44^, ^45^, ^46^, ^52^, ^53^, ^54^, ^56^, ^58^, ^62^, ^63^ In the meta-analysis, smoke-free legislation was associated with a significant immediate reduction in preterm births (ten studies, 27 530 183 individuals; −3·77% [95% CI −6·37 to −1·16]; figure 2A). Two studies caused some funnel plot asymmetry suggestive of publication bias, but this asymmetry was unlikely to have affected our findings (appendix p 34). No additional gradual change in preterm births was evident (two studies, 1 316 633 individuals; −0·01% per year [95% CI −6·76 to 6·73]; figure 3A). One study^47^ examined the association between provision of smoking cessation services and preterm births. Medicaid enrolment policies permitting low-income pregnant women to receive smoking cessation services were not associated with a change in preterm births (table 2).^47^ Reductions in preterm birth were observed after tobacco tax increases among women in specific population subgroups in two studies.^46^, ^53^ One study reported tobacco taxation to be associated with reduced rates of preterm birth among white mothers with low levels of education and among black mothers irrespective of level of education (table 3).^46^ The other study reported a 0·7 percentage point decrease in preterm births per USD$ increase in tax among women aged 20–24 years, and a 1·0 percentage point decrease per USD$ increase in tax among women older than 34 years (table 3).^53^

Associations between smoke-free legislation and the incidence of hospital attendances for childhood asthma were reported in ten studies (table 1).^18^, ^36^, ^38^, ^40^, ^42^, ^49^, ^51^, ^55^, ^59^, ^61^ In the meta-analysis, both an immediate reduction in asthma exacerbations requiring hospital attendance (five studies, 684 826 events; −9·83% [95% CI −16·62 to −3·04]; figure 2B) and an additional gradual reduction were seen (four studies, 243 377 events; −5·94% per year [95% CI −11·48 to −0·41]; figure 3B). No change in asthma admissions was seen following a health reform legislation that provided smoking cessation services for Medicaid recipients in one study.^18^ Among three US studies^18^, ^49^, ^50^ with overlapping populations evaluating tobacco taxation and asthma exacerbations requiring hospital attendance, the study with the lowest risk of bias found no significant reductions following state-wide increases in cigarette excise tax (table 3).^18^

The association between smoke-free legislation and the incidence of hospital admissions for acute respiratory tract infections was reported in four studies (table 1).^16^, ^18^, ^19^, ^64^ In the meta-analysis, an immediate reduction was seen in respiratory tract infections (upper and lower respiratory tract infections combined) requiring hospital attendance (two studies, 1 681 020 events; −3·45% [95% CI −4·64 to −2·25]; figure 2C). For the studies that reported specifically on lower respiratory tract infections, the meta-analysis showed an immediate reduction in admissions for lower respiratory tract infections following smoke-free legislation (three studies, 887 414 events; −18·48% [95% CI −32·79 to −4·17]; figure 2D). No additional gradual reduction in lower respiratory tract infections was observed (two studies; 748 175 events: −6·81% per year [95% CI −20·63 to 7·01]; figure 3C). No significant association between smoke-free legislation and admissions for upper respiratory tract infections was seen in the meta-analysis (two studies; 1 390 056 events; 0·42% [95% CI −3·28 to 4·13]; figure 2E). One study^18^ reported that a health reform legislation that provided smoking cessation services for Medicaid recipients was associated with an immediate −6% (95% CI −10 to −1) decrease in hospital admissions for childhood upper respiratory tract infection, but not in admissions for lower respiratory tract infection (table 2). The same study^18^ evaluated the effect of tobacco taxation, showing a −9% decrease (95% CI −16 to −2) in lower respiratory tract infections requiring admission to hospital per USD$ increase in cigarette excise tax at the state level (table 3).

We did not identify any studies assessing the effect of other MPOWER policies on child health.

In sensitivity analyses, inclusion of non-EPOC studies in the meta-analyses or restriction of the primary analyses to studies with low to moderate risk of bias did not materially change the effect estimates for smoke-free legislation and our primary outcomes (appendix pp 35–39).

Point estimates for the association between smoke-free legislation and our primary outcomes were generally much larger when subgroup analyses were restricted to studies assessing comprehensive smoke-free laws than when studies assessing partial smoke-free laws were analysed (preterm birth: seven studies, 9 355 359 individuals, −5·12% [95% CI −7·24 to −2·99]; hospital attendances for asthma: four studies, 556 019 events, −12·49% [–19·78 to −5·20]; appendix, pp 40–44).

11 studies assessed whether the association between implementation of tobacco control policies and child health varied according to indicators of socioeconomic status (appendix pp 45, 46).^16^, ^29^, ^30^, ^33^, ^44^, ^46^, ^51^, ^54^, ^55^, ^62^, ^63^ One study^16^ showed that the most deprived children experienced the largest gradual reduction in hospital admissions for respiratory tract infection following smoke-free legislation (–1·5% per year [95% CI −2·1 to −1·0]). In two studies,^44^, ^46^ improvements in perinatal outcomes were greater among babies born to parents with low levels of education following smoke-free legislation than among those born to parents with high levels of education,^44^ and among babies born to black mothers with any level of education and to white mothers with low levels of education following tobacco tax increases.^46^ Other studies did not identify a clear socioeconomic gradient in the association between tobacco control policies and child health.

27 studies assessed the association between tobacco control policies and secondary outcomes (appendix, pp 47–76). In the meta-analyses (appendix pp 77–85), smoke-free legislation was associated with immediate reductions in very preterm birth (five studies; 3 354 636 individuals; −9·99% [95% CI −15·74 to −4·24]), low birthweight (nine studies; 35 206 918 individuals, −2·77% [–4·36 to −1·19]), and small for gestational age births (eight studies; 27 649 380 individuals; −1·84% [–3·21 to −0·47]), a gradual reduction in very small for gestational age births (two studies; 1 298 276 individuals; −0·60% per year [–0·60 to −0·60]), and a small increase in birthweight (seven studies; 3 238 575 individuals; 12·45 g [95% CI 2·09–22·81]). No significant changes in other secondary outcomes were seen following smoke-free legislation. Legislation to promote prenatal care, including smoking cessation services for low-income pregnant women, was not associated with a change in small for gestational age births in one US study.^47^ In another US study,^28^ although such legislation was associated with increased duration of gestation, depending on time of enrolment (308 521 participants; 0·063 weeks [95% CI 0·008–0·118] among women who enrolled in the Medicaid insurance programme before or during pregnancy and 0·086 weeks [0·004–0·168] among women who enrolled during pregnancy), it was not associated with a change in birthweight (appendix pp 66, 67). One study showed reductions in extremely and very preterm births following tobacco tax increases,^53^ with two others also showing an increase in gestation.^28^, ^35^ Among five studies assessing the link between tobacco tax and birthweight, two showed a positive effect,^39^, ^46^ albeit of very small magnitude. Accordingly, only one of these five studies showed a reduction in low birthweight following tobacco tax increases.^46^ This study also found reductions in small for gestational age births; both associations were confined to low socioeconomic groups.^46^ In two studies assessing very low birthweight, no changes were seen following tobacco tax increases.^39^, ^53^ Tobacco taxes were associated with a decreased risk of infant mortality in two studies assessing this association.^57^, ^60^ In one of these studies, however, an increase in fetal deaths was also observed.^60^ One study showed significant reductions in paediatric asthma prevalence following tobacco tax increases.^33^

## Discussion

This systematic review and meta-analysis provides considerable evidence indicating child health benefits associated with implementation of MPOWER policies. By pooling data of 27·5 million births, 685 000 hospital admissions for asthma, and 2·3 million hospital admissions for respiratory tract infections, we found a 3·7% reduction in preterm births, a 9·8% reduction in childhood hospital admissions for asthma, and an 18·5% reduction in hospital admissions for lower respiratory tract infections following implementation of smoke-free legislation. Subgroup analyses suggested that health benefits were increased when the most comprehensive laws were applied. We also identified several studies indicating that tobacco tax increases and governmental support for smoking cessation services could benefit child health. Taken together with substantial existing evidence on the effectiveness of tobacco control policies in improving adult health, these findings provide strong support for implementation of such policies comprehensively across the world.

This study is, to our knowledge, the most comprehensive assessment done to date of the effect of tobacco control policies on perinatal and child health outcomes. On the basis of our previous work,^15^ and the challenges of evaluating governmental policies through randomised trials,^69^, ^70^ we anticipated that most eligible studies would be of quasi-experimental design. We therefore followed EPOC guidelines to restrict our primary analyses to study types that were considered to be at lowest risk of bias. We confirmed the robustness of our findings via a number of prespecified sensitivity analyses, which indicated that our findings were not sensitive to exclusion of studies with a high risk of bias or inclusion of purely observational studies. Our work builds on existing evidence since it focuses on all available evidence on the effect of tobacco control policies on perinatal and child health. The consistency of this evidence, in our view, supports the validity of our findings.

However, our study has some limitations. The risks of residual confounding and bias in quasi-experimental studies—due to non-random allocation of the intervention and the absence of a control group—need to be considered when interpreting the results.^71^ Additional limitations include between-study heterogeneity in methodology, differences in follow-up duration and diagnosis ascertainment, the absence of assessment of the likely causal pathways between the policies and their health effects in several studies, and the low number of studies in each meta-analysis, which precluded assessment of publication bias for most outcomes and the use of meta-regression.

This study adds to our previous work.^15^ We identified an additional 24 studies on the effect of smoke-free legislation on child health, comprising additional data from more than 10 million births, 4·6 million GP diagnoses, and 2·2 million hospital admissions. These additional studies allowed us, for the first time, to identify the association between smoke-free legislation and reductions in severe respiratory tract infections, which is particularly relevant since respiratory tract infections account for the vast majority of the global burden of disease resulting from second-hand smoke exposure in children.^1^ We also broadened the scope of this study to include all MPOWER policies, identifying several studies on the effect of tobacco tax increases and smoking cessation services on child health. We also identified one study evaluating a tobacco control policy that could not be classified according to WHO's MPOWER Framework. Following an increase in the minimum legal age to purchase cigarettes from 18 years to 21 years in the US state of Pennsylvania, a 1·4 (95% CI −2·6 to −0·2) percentage point reduction in low birthweight was observed, which was largest among smoking mothers and associated with a significant reduction in prenatal cigarette consumption.^72^

Socioeconomic disparities in smoking and related morbidity are widely documented and affect both adults and children. For example, such disparities were estimated to account for 38% of the inequality in stillbirths and 31% of the inequality in infant deaths in Scotland.^73^ Previous systematic reviews^74^, ^75^, ^76^ showed that, among MPOWER measures, tobacco taxation has the greatest potential to reduce socioeconomic disparities associated with smoking in both young people and adults. We identified some evidence suggesting a pro-equity effect of both tobacco taxation and smoke-free legislation on early-life health. Since smokers are over-represented among deprived communities, such relative benefits of tobacco control policies translate into larger absolute effects in children from low socioeconomic groups than in children from high socioeconomic groups.

Given the inherent restrictions in attributing causality from quasi-experimental studies, it is important to interpret the findings in light of circumstantial evidence supporting the link between tobacco control policies and child health benefits. We have previously described the main likely causal pathways.^23^ Tobacco smoke exposure during fetal stages and childhood is associated with various adverse perinatal and child health outcomes.^4^, ^5^, ^6^, ^8^, ^77^, ^78^, ^79^, ^80^, ^81^, ^82^, ^83^ Several studies have shown substantial reductions in maternal smoking^31^, ^34^, ^52^, ^56^, ^84^, ^85^ and in second-hand smoke exposure among adults (including pregnant women) and children after implementation of tobacco control policies (appendix pp 86–89).^12^, ^13^, ^86^, ^87^, ^88^, ^89^, ^90^ Whereas smoke-free laws specifically target public spaces, various studies have shown subsequent increases in smoking cessation and reduced initiation,^12^, ^91^, ^92^ as well as changes in social norms leading to decreased smoking in the home environment,^93^, ^94^, ^95^, ^96^, ^97^ which is probably the primary source of second-hand smoke exposure among children. Our study provides further support for a causal association, since we found the largest decreases in our outcomes of interest when comprehensive smoke-free legislation was considered. This observation is suggestive of a dose–response association, which has previously also been identified for adult studies.^98^ Because of the low number of studies in individual meta-analyses we did not formally test for this interaction, and future efforts to do so might strengthen our findings as more evidence becomes available.

The global health burden of tobacco use is tremendous and its total global economic cost is estimated to be around USD$1·4 trillion.^99^ Despite global progress in tobacco control, over a third of the world's population remains unprotected by any MPOWER policy at the recommended level.^2^, ^11^ This issue is important because 40–50% of children worldwide are regularly exposed to tobacco smoke, and tobacco control policies have substantial potential to reduce the associated burden of death and disease.^1^ This global burden is acknowledged by the prioritisation within SDG 3 of more effective FCTC implementation and its aim to reduce early-life mortality; our data now show that these initiatives can act synergistically. Because our effect estimates are expressed as relative changes, background prevalence of smoking and second-hand smoke exposure, and of the health outcomes evaluated, should be considered when extrapolating our findings to local contexts. We did not formally assess the comparative effectiveness or cost-effectiveness of different MPOWER policies. Tax increases are considered to be the most effective measure to reduce smoking prevalence,^2^ and although our review indicates that tobacco taxation is likely to be associated with child health benefits, the evidence was particularly strong for smoke-free legislation. Smoke-free laws are the tobacco control policy most strongly supported by the public and appear to be the most straightforward measure to protect child health, particularly when implemented comprehensively.^12^ The synergistic effect of various policies implemented at the highest recommended levels in reducing smoking prevalence should be considered when planning policy changes,^14^ which, when implemented as part of a strong tobacco control programme, can be highly cost-effective.^100^, ^101^ Ongoing monitoring is needed to continue to evaluate the effectiveness of policies aimed at reducing the impact of tobacco, in particular the effectiveness of novel endgame strategies targeted at ending rather than controlling the global tobacco epidemic.^102^

Reports indicate that at least two of five people living in low-income and middle-income countries remain unprotected by any MPOWER policy measure,^2^ and that wide variations in implementation and compliance are present across these countries.^103^ This finding is of concern, since these countries have the largest burden of tobacco-related illness and death, and harbour nearly 80% of the world's smokers.^2^ We highlight an important gap in the literature as more research is required in low-income and middle-income countries to understand the effect of tobacco control policies in these regions. Modelling approaches are increasingly being used to estimate the effect of tobacco control policies in low-income and middle-income countries, and original studies are now becoming available.^104^, ^105^ Efforts are underway to address the current absence of a child health focus in this area, which will be essential to inform the global policy agenda. Furthermore, we found no studies specifically evaluating early-life health outcomes in relation to legislation to prohibit tobacco advertising and sponsorship, or warnings against the dangers of tobacco. Priority should be given to establishing a core set of outcomes related to perinatal and child health, alongside adult health, for all future studies examining the effect of tobacco control policies.

In conclusion, given the positive findings of this systematic review it is crucial that the uptake of comprehensive tobacco control policies is accelerated worldwide to further protect children from the health hazards of tobacco smoke exposure,^106^ in parallel with efforts to evaluate the effectiveness of novel policy initiatives.
