# Supplementary material for: Effect of tobacco control policies on perinatal and child health: a systematic review and meta-analysis
Source: Lancet Public Health. 2017 Sep 5;2(9):e420–37. doi: 10.1016/S2468-2667(17)30144-5 (PMC5592249; doi:10.1016/S2468-2667(17)30144-5)
Supplement: Supplementary appendix [file mmc1.pdf]

THE LANCET
Public Health

Supplementary appendix

This appendix formed part of the original submission and has been peer reviewed. We post it as supplied by the authors.

Supplement to: Faber T, Kumar A, Mackenbach JP, et al. Effect of tobacco control policies on perinatal and child health: a systematic review and meta-analysis. *Lancet Public Health* 2017; **2:** e420–37.

SUPPLEMENTARY MATERIAL: TABLE OF CONTENTS

Appendix 1:	MEDLINE search strategy	p 1
Appendix 2:	Panel of experts in the field	p 2
Appendix 3:	Data extraction form	pp 3–5
Table S1:	Studies excluded from systematic review	pp 6–7
Table S2:	Characteristics of included EPOC studies	pp 8–20
Table S3:	Characteristics of included non-EPOC studies	p 21
Table S4:	Model specifications of included studies	pp 22–31
Table S5:	Risk of bias assessment	pp 32–33
Figure S1:	Funnel plot of studies assessing association between smoke-free legislation and preterm birth	p 34
Figure S2:	Sensitivity analysis including non-EPOC studies	p 35
Figure S3:	Sensitivity analysis including only studies with low and moderate risk of bias	pp 36–39
Figure S4:	Subgroup analysis on comprehensiveness of smoke-free legislation	pp 40–44
Table S6:	Variations in outcomes according to socioeconomic status	pp 45–46
Table S7:	Association between implementation of tobacco control policies and secondary outcomes	pp 47–76
Figure S5	Meta-analysis of immediate changes in secondary outcomes after implementation of smoke-free legislation	pp 77–83
Figure S6	Meta-analysis of gradual changes in secondary outcomes after implementation of smoke-free legislation	pp 84–85
Table S8:	Association between tobacco control policies and smoking behaviour in included studies	pp 86–89
References		pp 90–91

Appendix 1. MEDLINE search Strategy

We searched: Cochrane Central Register of Controlled Trials (CENTRAL), MEDLINE, EMBASE, PsycINFO, Cumulative Index to Nursing and Allied Health Literature (CINAHL), WHO Global Health Library (in addition to MEDLINE covering African Index Medicus (AIM), LILACS, Index Medicus for the Eastern Mediterranean Region (IMEMR), Index Medicus for South-East Asia Region (IMSEAR), Western Pacific Region Index Medicus (WPRIM), WHO Library Database (WHOLIS), and Scientific Electronic Library Online (SciELO)), IndMED, ISI Web of Science, KoreaMed, EconLit, Paediatric Economic Database Evaluation (PEDE), Google Scholar, the ProQuest database of PhD dissertations, and WHO International Clinical Trials Registry Platform (ICTRP) to identify any ongoing/unpublished studies. No language restrictions were placed on the searches.

MEDLINE (Ovid): 1946 to October Week 4

1 exp "Tobacco Use"/ or Tobacco Smoke Pollution/ or Smoke-Free Policy/ or exp "Tobacco Use Cessation Products"/ or "Tobacco Use Cessation"/ or "Tobacco Use Disorder"/ or nicotine/ or Tobacco Products/ or tobacco/ or cotinine/ or tobacco industry/ or (smoking or antismoking or nonsmoking or smoke or tobacco* or cigarette* or cigar* or nicotine or cotinin* or antitobacco).ab,ti. (320311)

2 (Social Control, Formal/ or exp Jurisprudence/ or Law Enforcement/ or exp Mandatory Programs/ or Government Regulation/ or "legislation and jurisprudence".xs. or exp policy/ or exp politics/ or exp government/ or exp mass media/ or (regulation* or government* or law or laws or policy or policies or smokefree or ((smoking or smoke) adj3 (ban or bans or banned or free or restrict* or act or acts or price or pricing or tax or taxes or taxation)) or legal* or illegal* or hotline* or quitline* or (mass adj (media or medium or communicat*)) or ordinanc* or prohibit* or decree* or enactment* or mandator* or campaign* or ((pictorial or graphic) adj3 warning*) or packaging or mpower or advertizing or advertising or legislat* or statut* or ((population-based or public health) adj3 strateg*)).ab,ti.) and (exp child/ or exp infant/ or exp pediatrics/ or exp Child Health Services/ or Hospitals, Pediatric/ or (infan* or newborn* or (new adj born*) or baby or babies or neonat* or child* or kid or kids or toddler* or boy* or girl* or minors or underag* or (under adj (age* or aging)) or youth* or kindergar* or prepubescen* or prepubert* or pediatric* or paediatric* or school* or preschool* or intrauterine or intra-uterine or prenatal or antenatal or (age adj3 analy*)).ab,ti.) (181733)

3 (exp epidemiology/ or Epidemiologic Methods/ or Epidemiological Monitoring/ or exp Epidemiologic Research Design/ or exp Data Collection/ or Biomedical Research/ or Clinical Trial.pt. or Controlled Clinical Trial.pt. or Multicenter Study.pt. or Observational Study.pt. or Randomized Controlled Trial.pt. or Evaluation Studies.pt. or exp cohort studies/ or Program Evaluation/ or (epidemiol* or clinical* or intervent* or trial* or random* or rct* or prospective* or retrospect* or cohort* or longitudinal* or time series or "difference in difference" or "before and after" or effectiv* or Questionnaire* or associat* or survey* or evaluat* or observat* or (follow* adj up*) or followup or compar* or impact*).ab,ti.) not (letter or news or comment or editorial or congresses or abstracts).pt. (11994259)

4 1 and 2 and 3 (6898)

Appendix 2: Panel of consulted experts in the field

Prof. A. Amos
Prof. S. Glantz
Dr. S.S. Hawkins
Dr. A. Hyland
Dr. Z. Kabir
Prof. D.T. Levy
Dr. D. Mackay
Dr. S. Markowitz
Prof. A. Gilmore
Dr. E.Tursan D'Espaignet
Prof. M. Willemsen

Appendix 3. Data extraction form

General information
Article ID: Click here to enter text.
Data extractor: Choose an item.
Date: Click here to enter a date.
Article title: Click here to enter text.
Author names: Click here to enter text.
Author affiliations: Click here to enter text.
Publication year: Click here to enter text.
Type of publication (journal, book, dissertation etc.): Click here to enter text.
URL (if applicable): Click here to enter text.
Study funders: Click here to enter text.
Conflicts of interest reported: Click here to enter text.

Study characteristics
Study type: Click here to enter text.
Study site(s): Click here to enter text.
Study period(s): Click here to enter text.
Data sources used: Click here to enter text.

Participants
Number of clusters and cluster sizes (if relevant): Click here to enter text.
Number of participants: Click here to enter text.
Treatment group sizes: Click here to enter text.
Inclusion/exclusion criteria: Click here to enter text.
Participant characteristics (age, gender, socioeconomic status): Click here to enter text.

Intervention(s)
Description of intervention(s): Click here to enter text.
Timing of intervention(s): Click here to enter text.
Measure of degree/comprehensiveness of intervention(s): Click here to enter text.
Description of comparator: Click here to enter text.
MPOWER Category: Click here to enter text.

Outcome measures and definitions
Primary outcomes:
☐ Perinatal mortality (stillbirth + neonatal mortality)
☐ Preterm birth (live birth at gestational age <37 weeks)
☐ Asthma exacerbations requiring hospital attendance (emergency department visit and/or hospital admission):
☐ Respiratory tract infections requiring hospital attendance (emergency department visit and/or hospital admission:

Specify the outcome definition used by authors: Click here to enter text.

Secondary outcomes:
Mortality:
☐ Stillbirth (born dead at gestational age ≥ 24 weeks)
☐ Neonatal mortality (death within 28 days)
☐ Early neonatal mortality (death within 7 days)
☐ Late neonatal mortality (death between 7 and 28 days)
☐ Postneonatal mortality (death between 28 days and 1 year)
☐ Infant mortality (death in the first year of life)
☐ Child mortality

Please specify if the definition of outcome(s) used are different than described above: Click here to enter text.

Perinatal outcomes among live births:
☐ Extremely low birth weight (birth weight <1000 g)
☐ Very low birth weight (birth weight <1500 g)
☐ Low birth weight (live birth with birth weight <2500 g)
☐ Birth weight (continuous scale)
☐ Small for gestational age (birth weight <10th centile for gestational age)
☐ Very small for gestational age (birth weight <3rd centile for gestational age)
☐ Extremely preterm birth (gestational age <28 weeks)
☐ Very preterm birth (gestational age <32 weeks)
☐ Gestational age (continuous scale)
☐ Congenital anomalies

Please specify if the definition of outcome(s) used are different than described above: Click here to enter text.

Childhood outcomes:
☐ Asthma
☐ Wheezing
☐ Respiratory infections
☐ Upper respiratory infections
☐ Lower respiratory infections
☐ Otitis media with effusion
☐ Chronic cough

Extra information:
☐ Changes in maternal (when pregnant) and/or child SHS exposure
☐ Changes in maternal smoking during pregnancy
☐ Changes in paternal smoking behaviour
☐ Changes in child/adult smoking behaviour

Primary Outcomes:

**[Outcome measure]**				**Page/Paragraph in article**
	**Total**	**Before intervention**	**After Intervention**	
**Population at risk (n)**				
**Events (n)**				
**Rates (%)**				
	**Unadjusted relative / absolute change (95% CI)**	**Adjusted relative / absolute change (95% CI)**	**Covariates adjusted for**	**Page/Paragraph in article**
**Association between intervention and outcome**				

Secondary Outcomes:

[Outcome measure]				Page/Paragraph in article
	**Total**	**Before intervention**	**After Intervention**	
**Population at risk (n)**				
**Events (n)**				
**Rates (%)**				
	**Unadjusted relative / absolute change (95% CI)**	**Adjusted relative / absolute change (95% CI)**	**Covariates adjusted for**	**Page/Paragraph in article**
**Association between intervention and outcome**				

Extra information:

Outcome	Definition	Change

If clustered study, are the results adjusted for clustering? Choose an item.
If yes, is the ICC reported? Choose an item.
If so, what is this? Click here to enter text.

Statistical analysis technique(s) used: Click here to enter text.
Bias assessment: Click here to enter text.
Adverse effects: Click here to enter text.
Follow-up rate: Click here to enter text.
Handling of dropouts: Click here to enter text.

Reviewer's comments: Click here to enter text.

Do we need to contact the author for further details? Choose an item.
Contact details: Click here to enter text.

Table S1: Studies excluded from systematic review

Study		Reason for Exclusion
1.	Duffin, C., Smoking ban linked to fall in number of babies born early. *Nursing Children & Young People* 2012; **24**(3): p.5–5.	Article reports on an already included study
2.	Fernández Rodríguez M. and Orejón G. La ley antitabaco en espacios públicos se relaciona con la disminución de ingresos por asma infantile. The smoking ban in public places is related to the decrease in child asthma admissions. *Rev Pediatr Aten Primaria* 2013; **15**(60): p.371–374.	Article reports on an already included study
3.	Galan, I., et al., Assessing the effects of the Spanish partial smoking ban on cardiovascular and respiratory diseases: methodological issues. *BMJ Open* 2015; **5**(12): p.e008892.	Participants do not fit the inclusion criteria
4.	Harris JE, Balsa AI, Triunfo P. Tobacco control campaign in Uruguay: Impact on smoking cessation during pregnancy and birth weight. *J Health Econ* 2015; **42**: p.186–196.	Study design does not fit the inclusion criteria
5.	Holford, T.R., et al., Tobacco Control and the Reduction in Smoking-Related Premature Deaths in the United States, 1964–2012. *JAMA* 2014; **311**(2): p.164–171.	Participants do not fit the inclusion criteria
6.	King C, Markowitz S, Ross H. Tobacco control policies and sudden infant death syndrome in developed nations. *Health Econ* 2015; **24**(8): p.1042–1048.	Study design does not fit the inclusion criteria
7.	Koh, H.K., et al., The first decade of the Massachusetts tobacco control program. *Public Health Rep* 2005; **120**(5): p.482–495.	Outcome of interest not measured
8.	Lien, D. and W. Evans, Estimating the impact of large cigarette tax hikes the case of maternal smoking and infant birth weight. *Journal of Human resources* 2005.	Study design does not fit the inclusion criteria
9.	Lin, H.C., J.Y. Park, and D.C. Seo, Comprehensive US Statewide Smoke-Free Indoor Air Legislation and Secondhand Smoke Exposure, Asthma Prevalence, and Related Doctor Visits: 2007–2011. *Am J Public Health* 2015; **105**(8): p.1617–1622.	Outcome of interest not measured
10.	Markowitz S. The effectiveness of cigarette regulations in reducing cases of Sudden Infant Death Syndrome. *J Health Econ* 2008; **27**(1): p.106–133.	Study design does not fit the inclusion criteria
11.	Naiman, A., R.H. Glazier, and R. Moineddin, Association of anti-smoking legislation with rates of hospital admission for cardiovascular and respiratory conditions. *CMAJ* 2010; **182**(8): p.761–767.	Participants do not fit the inclusion criteria
12.	Oude Wesselink, S.F., et al., Provision and effect of quit-smoking counselling by primary care midwives. *Midwifery* 2015; **31**(10): p.986–992.	Intervention does not fit the inclusion criteria
13.	Reading, R., Impact of a stepwise introduction of smoke-free legislation on the rate of preterm births: Analysis of routinely collected birth data. *Child: Care, Health and Development* 2013; **39**(4): p.615.	Article reports on an already included study
14.	Reading, R., Hospital admissions for childhood asthma after smoke-free legislation in England. *Child: Care, Health and Development* 2013; **39**(3): p.458.	Article reports on an already included study
15.	Sexton, M. and J. Hebel, A clinical trial of change in maternal smoking and its effect on birth weight. *JAMA* 1984; **251**(7): p.911–915.	Intervention does not fit the inclusion criteria
16.	Silva, R., et al., Preventing low birth weight in Illinois: Outcomes of the Family Case Management Program. *Maternal and Child Health Journal* 2006; **10**(6): p.481–488.	Intervention does not fit the inclusion criteria
17.	Stein, C.R., et al., Decline in smoking during pregnancy in New York City, 1995–2005. *Public Health Rep* 2009; **124**(6): p.841–849.	Outcome of interest not measured
18.	Walker, N., et al., Effect of a family-centered, secondhand smoke intervention to reduce respiratory illness in indigenous infants in Australia and New Zealand: A randomized controlled trial. *Nicotine Tob Res* 2015; **17**(1): p.48–57.	Intervention does not fit the inclusion criteria
19.	Yan J. The effects of a minimum cigarette purchase age of 21 on prenatal smoking and infant health. *East Econ J* 2014; **40**(3): p.289–308.	Intervention does not fit the inclusion criteria
20.	Yildiz, F., et al., Role of smoke-free legislation on emergency department admissions for smoking-related diseases in Kocaeli, Turkey. *East Mediterr Health J* 2014; **20**(12): p.774–780.	Participants do not fit the inclusion criteria

21.	Zulkifli, A., et al., Implementation of smoke-free legislation in Malaysia: are adolescents protected from respiratory health effects? *Asian Pac J Cancer Prev* 2014; **15**(12): p.4815–4821.	Participants do not fit the inclusion criteria

Table S2: Characteristics of included EPOC studies

MPOWER: Protect people from tobacco smoke (i.e. smoke-free legislation)														
**Study (year)**	**Country (region)**	**Area pop ($x10^6$)***	**Study design**	**Intervention**				**Age of participants**	**Outcome**				**Summary risk of bias†**
				**Details**	**Level**	**Date**	**Previous intervention in place**		**Eligible outcomes**	**Definition**	**Data source**	**Inclusion criteria**	
Adams (2013)[1]	USA (multiple states)	197·73	CITS	All restaurants	State	Various timings	Unclear	Neonates	Primary: None<br><br>Secondary: BW; GA	BW in g; GA in wk	Pregnancy Risk Assessment Monitoring System; birth certificates	Singleton live births	Low
Amaral (2009)[2]	USA (California)	38·04	CITS	Public places and workplaces (including restaurants)	State and local (county/city)	01/01/1995 (state); variable for local	None (in intervention group)	Neonate	Primary: None<br><br>Secondary: BW; GA; LBW; VLBW	BW in g;<br>GA in days;<br>LBW: BW<2500g;<br>VLBW: BW<1500g	Birth certificates (California Department for Health Services)	GA ≤ 4 SD	High
Bakolis (2016)[3]	UK (England)	53·01	Regression discontinuity	Public places and workplaces (including restaurants and bars)	National	01/07/2007	No previous smoke-free law	Neonates	Primary: PTB<br><br>Secondary: BW; GA; LBW; SGA; VLBW	PTB: GA<37 wk<br><br>BW in g;<br>GA in days;<br>LBW: BW<2500g;<br>SGA: BW<p10 for GA and sex using centiles derived from all eligible births;<br>VLBW: BW<1500g	Hospital Episode Statistics	Singleton live births, GA 24−44 wk, BW 200−5000g, sex known, non-intersex infants, maternal age 15−44 years	Low
Bartholomew (2016)[4]	USA (West Virginia)	1·85	ITS	1. Comprehensive (workplaces, restaurants, bars)<br><br>2. Restrictive (workplaces, restaurants, no restriction in bars)<br><br>3. Moderate (workplaces, partial restriction in restaurants, no restriction in bars)<br><br>4. Limited (partial	County	Various timings	Variable	Neonates	Primary: PTB<br><br>Secondary: BW; GA; LBW; VLBW	PTB: GA<37wk<br><br>BW in g;<br>GA in days;<br>LBW: BW<2500g;<br>VLBW: BW<1500g	West Virginia Vital Statistics	Singleton live births	Low

				restriction in workplaces, any restriction in restaurants, no restriction in bars)									
Been, Mackay (2015)[5]	UK (England)	53·01	ITS	Public places and workplaces (including restaurants and bars)	National	01/07/2007	No previous smoke-free law	Neonates	Primary: None<br><br>Secondary: Early neonatal mortality; infant mortality; late neonatal mortality; LBW; neonatal mortality; post neonatal mortality; stillbirth; VLBW	Early neonatal mortality: death in 1st wk of life; Infant mortality: death within the 1st yr of life; Late neonatal mortality: death between 7 and 28 days of life; LBW: BW<2500g; Neonatal mortality: death in the 1st 28 days of life; Post-neonatal mortality: death between 28 days of life and the first birthday; stillbirth: intrauterine death from GA 24wk; VLBW: BW<1500g	Office for National Statistics, linked to death certificates	Singleton live births	Low
Been, Millett (2015)[6]	UK (England)	53·01	ITS	Public places and workplaces (including restaurants and bars)	National	01/07/2007	No previous smoke-free law	Children aged 0 to 14 years	Primary: Acute RTI hospital admissions; acute LRTI hospital admissions; acute URTI hospital admissions<br>Secondary: None	ICD−10 codes. LRTIs: J10.0, J11.0, J12–J18, J20–J22 and J40–J42;<br><br>URTIs: A37, H66–H67, J02.0, J00–J06 and J09–J11 (excluding J10.0 and J11.0)	Hospital Episode Statistics	Children aged ≤ 14 years	Low
Been, Szatkowski (2015)[7]	UK	64·1	ITS	Public places and workplaces (including restaurants and bars)	National	01/07/2007 (England); 26/03/2006 (Scotland);	No previous smoke-free laws	Children aged 0 to 12 years	Primary: None<br><br>Secondary:	Diagnostic Read codes	Clinical Practice Research Datalink	Children aged ≤ 12 years	Low

						02/04/2007 (Wales); 30/04/2007 (Northern Ireland)			GP RTI diagnoses; GP LRTI diagnoses (England only); GP URTI diagnoses (England only); GP wheezing/ asthma diagnoses				
Bharadwaj (2014)[8]	Norway	4·95	CITS	Restaurants and bars	National	01/06/2004	Public places, workplaces (excluding restaurants and bars)	Neonates	Primary:<br>PTB<br><br>Secondary:<br>BW; congenital anomalies; ELBW; LBW; VLBW	PTB: GA<36 wk<br><br>BW in g; congenital anomalies: a form of birth defect;<br>ELBW: BW<1000g;<br>LBW: BW<2500g;<br>VLBW: BW<1500g	Medical Birth Registry of Norway	Mothers who worked in a shop, restaurant, or bar	Moderate
Briggs (2012)[9]	USA (multiple states)	Unknown	CITS	Workplaces: 100% smoke-free; qualified‡; some coverage§<br><br>Restaurants: 100% smoke-free; qualified‡; some coverage§<br><br>Bar: 100% smoke-free; qualified‡; some coverage§	State and local	Various timings	No previous smoke-free law	Neonates	Primary:<br>PTB<br><br>Secondary:<br>GA	PTB: GA<37 wk<br><br>BW in g;<br>LBW: BW<2500g	Natality Detail File 1989 to 2004; National Vital Statistics	Singleton live births, mothers living in counties with population >100,000	Moderate
Ciaccio (2016)[10]	USA (multiple regions)	13·25	CITS	Indoor public places (various policies)	State and local	Various timings	Unknown	Children aged 0 to 17 years	Primary:<br>Asthma ED visits	ICD−9-CM code 493	Pediatric Health Information System	Children aged ≤ 17 years	Low
Cox (2013)[11]	Belgium (Flanders)	6·25	ITS	1. Public places and workplaces (excluding catering industry)<br>2. As above but including restaurants<br>3. As above but including bars serving	National	1. 01/01/2006<br>2. 01/01/2007<br>3. 01/01/2010	1. None<br>2. Public places, workplace (excluding catering industry)	Neonates	Primary:<br>PTB<br><br>Secondary:<br>BW; LBW; SGA	PTB: GA<37 wk<br><br>BW in g;<br>LBW: BW<2500g;<br>SGA: BW<p10	Study Centre for Perinatal Epidemiology	Singleton live births; BW>500g; GA 24−44 wk	Low

				food			3. Public places, workplace (including restaurants, not bars)			for GA			
Croghan (2015)[12]	USA (Olmsted County, Minnesota)	0·15	ITS	Public places and workplaces (including restaurants and bars)	State	16/05/2007 (Law passed);<br><br>01/10/2007 (Law enacted)	No previous smoke-free law	Children aged 0 to 18 years	Primary: Asthma-related ED visits<br><br>Secondary: None	ICD−9 code 493	The Rochester Epidemiology Project (Mayo Clinic and Olmsted Medical Center)	Children aged ≤ 18 years	High
Galán (2017)[13]	Spain (five provinces)	6·92	ITS	1st smoke-free law: Complete smoke-free workplaces and partial smoke-free restaurants and bars<br><br>2nd smoke-free law: Public places and workplaces (including restaurants and bars)	National	1st smoke-free law: 01/01/2006<br><br>2nd smoke-free law: 01/01/2011	1st smoke-free law: None<br><br>2nd smoke-free law: Complete smoke-free workplaces and partial smoke-free restaurants and bars	Children aged 0 to 14 years	Primary: Hospital admissions via EDs due to asthma	ICD−9 code 493	Hospital Discharge Records Database of the Spanish National Health System (CMBD-H)	Children aged ≤14 years	Low
Gao (2017)[14]	USA (multiple states)	Unknown	CITS	Public places and workplaces (including restaurants and bars)	State and local (county/sub-county)	Various timing	Variable	Neonates	Primary: None<br><br>Secondary: GA; BW; LBW; VLBW	BW in g<br>LBW: BW<2500g;<br>VLBW: BW<1500g;	Natality Detail File 1995 to 2009	Singleton live births, maternal age 14−45 yrs (with complete data on BW and GA), living in counties with population >250,000	Moderate
Gaudreau (2013)[15]	Canada (Prince Edward Island)	0·14	ITS	Public places and workplaces (including restaurants and bars), allowing designated smoking areas	Province	01/06/2003	Unclear	0−14 years	Primary: Acute care hospital asthma admissions<br><br>Secondary: None	ICD−9 493, ICD−10 J45/46	Discharge Abstract Database	Children aged ≤ 14 years	Moderate
Hade (2011)[16]	USA (Ohio)	11·54	ITS	Public places and workplaces (including restaurants and bars)	State	03/05/2007¶	Variable (regional smoke-free laws)	Neonates	Primary: PTB<br><br>Secondary: LBW	PTB: GA<37 wk<br><br>LBW: BW<2500g	Ohio certificates of livebirth	Singleton live births	Moderate

Hajdu (2017)[17]	Hungary	9·85	CITS	Public places and workplaces (including restaurants and bars)	National	Introduced on 01/01/2012, fully implemented on 01/04/2012	Smoking was allowed only in designated smoking areas in workplaces and public places. Smoking was allowed in restaurants and bars.	Newborns	Primary: PTB<br><br>Secondary: GA; VPTB; BW; LBW; VLBW; Infant mortality	PTB: GA<37 wk<br><br>GA in wk; VPTB: GA<32 wk; BW in g; LBW: BW<2500g; VLBW: BW<1500g; Infant mortality: death before 1 year of age	Hungarian Central Statistical Office	Working mothers (in restaurants/bars in intervention group and places other than restaurants/bars in control group)	Low
Hankins (2016)[18]	USA (multiple states)	Unknown	ITS	Workplaces: 100% smoke-free; qualified‡; some coverage§<br><br>Restaurants: 100% smoke-free; qualified‡; some coverage§<br><br>Bar: 100% smoke-free; qualified‡; some coverage§	State/county	Various timing	Variable	Neonates	Primary: PTB<br><br>Secondary: LBW	PTB: GA<37 wk<br><br>EPTB: GA<28 wk; LBW: BW<2500g; VLBW: BW<1500g; VPTB: GA<32 wk	Natality Detail Files from the National Center for Health Statistics, Centers for Disease Control and Prevention	Singleton live births that occurred in the same county as mother's county of residence	Low
Hawkins (2014)[19]	USA (multiple states)	139·68	ITS	100% smoke-free workplaces and restaurants	State	Various timings	Variable	Neonates	Primary: PTB<br><br>Secondary: BW; LBW; SGA	PTB: GA<37 wk<br><br>BW in g; LBW: BW<2500g; SGA: BW<p10 for GA and sex	US Natality Files (National Vital Statistics System (2000−2004); Public Health Statistics and Information Systems (2005−2010))	Singleton live births, GA 30−44 wk, maternal age 18−50 yrs (with complete data on smoking, maternal nativity, education, or BW), BW consistent with GA	Low

Hawkins (2016)[20]	USA (Massachusetts, New Hampshire, Vermont)	8·70	ITS	State or local 100% smoke-free workplaces and/or restaurants	State/county	Massachusetts: 05/07/2004 Smoke-free workplaces, restaurants<br><br>New Hampshire: 17/09/2007 Smoke-free restaurants<br><br>Vermont: 01/09/2005 Smoke-free restaurants 01/07/2009 Smoke-free workplaces<br><br>Local smoke-free legislation: variable	Variable	Children aged 0 to 17 years	Primary: Asthma ED visits; LRTI ED visits; URTI ED visits<br><br>Secondary: None	ICD−9-CM codes. Asthma: 493; LRTI: 466, 480−488; URTI: 460−465	Massachusetts: The Massachusetts Centre for Health Information and Analysis<br><br>New Hampshire: New Hampshire Department of Health and Human Services<br><br>Vermont: Green Mountain Care Board	Children aged ≤ 17 years	Low
Kabir (2013)[21]	Ireland	4·58	ITS	Public places and workplaces (including restaurants and bars)	National	29/03/2004	None	Neonates	Primary: None<br><br>Secondary: SGA; VSGA	SGA: BW<p5 for GA; VSGA: BW<p3 for GA	National Perinatal Reporting System	Singleton live births	Low
Landers (2014)[22]	USA (multiple states)	110·73	CITS	100% smoke-free workplaces, restaurants, and bars‖	State/county	Various timings	Variable	Children aged 0 to 19 years	Primary: Asthma discharges<br><br>Secondary: None	Asthma discharge rates	Healthcare Cost and Utilization Project	Children aged ≤ 19 years	Moderate
Lee (2016)[23]	China (Hong Kong)	7·19	ITS	Public places and workplaces (including restaurants)	City	01/01/2007	None	Children aged 0 to 18 years	Primary: LRTI hospital admissions<br><br>Secondary: None	ICD−9-CM codes for principal diagnosis on discharge: 464.1 to 464.4, 465.0, 466.0 to 466.1, 478.22, 478.24, 480, 482.0 to 482.2, 482.30 to 482.32, 482.4, 482.82 to 482.83, 483·0, 487.0, 486, 475,	Hospital Authority Hospitals central computerised database	Children aged ≤ 18 years	Moderate

										490, 510.9, 511, 513·0			
Mackay (2010)[24]	UK (Scotland)	5·30	ITS	Public places and workplaces (including restaurants and bars)	National	26/03/2006	None	Childre n aged 0 to 14 years	Primary: Emergency asthma admissions<br><br>Secondary: None	ICD−10 J45/46 (primary diagnosis)	Scottish Morbidity Record 01	Children aged ≤ 14 years	Low
Mackay (2012)[25]	UK (Scotland)	5·30	ITS	Public places and workplaces (including restaurants and bars)	National	26/03/2006	None	Neonat es	Primary: PTB<br><br>Secondary: LBW; SGA; VPTB; VSGA	PTB: GA<37 wk<br><br>LBW: BW<2500g; SGA: BW<p10 for GA; VPTB: GA<32 wk; VSGA: BW<p3 for GA	Scottish Morbidity Record 02	Singleton live births, GA 24−44 wk	Low
Markowitz (2013)[26]	USA (multiple states)	Unclear (29 states and New York City)	CITS	Workplace: complete smoke-free law, smoking restrictions (requiring designated smoking areas)<br><br>Restaurants: complete smoke-free law, smoking restrictions (requiring designated smoking areas)	State	Various timings	Variable	Neonat es	Primary: PTB<br><br>Secondary: BW; EPTB; GA; LBW; VLBW; VPTB	PTB: GA <37 wk<br><br>BW in g; EPTB: GA 20−27 wk; GA in wk; LBW: BW 1500−2499g; VLBW: BW<1500g; VTPB: GA 28−33 wk	Pregnancy Risk Assessment Monitoring System	Singleton live births	Low
McKinnon (2015)[27]	Canada (Quebec)	8·22	ITS	Public places and workplaces (including restaurants and bars)	State	31/05/2006	None	Neonat es	Primary: PTB<br><br>Secondary: BW; LBW; SGA; VPTB	PTB: GA<37 wk<br><br>BW in g; LBW: BW<2500g; SGA: BW<p10 for GA and sex; VPTB: GA<34 wk	Quebec provincial birth file	Singleton live births, GA 22−44 wk	Moderate
Millett (2013)[28]	UK (England)	53·01	ITS	Public places and workplaces (including restaurants and bars)	National	01/07/2007	None	Childre n aged 0 to 14 years	Primary: Emergency asthma hospital admissions	ICD−10 J45/46 (primary diagnosis)	Hospital Episode Statistics	Children aged ≤ 14 years	Moderate

									Secondary: None				
Page (2012)[29]	USA (Pueblo, El Paso, Colorado)	0·47	CITS	Public places and workplaces (including restaurants and bars)	City	01/07/2003	None	Neonates	Primary: PTB<br><br>Secondary: LBW	PTB: GA<37 wk<br><br>LBW: BW<2500g	Colorado birth registry	Singleton live births	Moderate
Peelen (2016)[30]**	Netherlands	16·8	ITS	1st smoke-free law: Workplaces and public transport except: restaurants and bars†† (allowing designated smoking areas)<br><br>2nd smoke-free law: Expanding smoke-free law 1 to include restaurants and bars‡‡ (allowing designated smoking areas)	National	1st smoke-free law: 01/01/2004<br><br>2nd smoke-free law: 01/07/2008	1st smoke-free law: None<br><br>2nd smoke-free law: Workplace and public transport except: hotels, restaurants and bars, including designated smoking areas	Neonates	Primary: Perinatal mortality, PTB<br><br>Secondary: Congenital anomalies; early neonatal mortality; LBW, SGA; stillbirth; VLBW; VSGA; VPTB	Perinatal mortality: all stillbirths and early neonatal mortality combined; PTB: GA<37 wk<br><br>Congenital anomalies§§; early neonatal mortality: death within the 1st 7 days after birth; LBW: BW<2500g; SGA: BW<p10; VLBW: BW<1500g; VSGA: BW<p2·3 for GA; VPTB: GA<32 wk	Netherlands Perinatal Registry	Singleton live births, BW>500g, no chromosomal anomalies, GA 24−42 wk	Low
Rayens (2008)[31]	USA (Lexington-Fayette, Kentucky)	0·30	ITS	Public buildings (including restaurants and bars) ||||	County	27/04/2004	None	Children aged 0 to 19 years	Primary: Asthma ED visits<br><br>Secondary: None	ICD−9 493 (primary or secondary diagnosis)	Individual hospital (n=4) ED discharge records	Children aged ≤ 19 years	Moderate
Shetty (2011)[32]	USA (multiple states)	Unknown (26 states)	ITS	1. All workplaces except restaurants and bars: 100% smoke-free<br><br>2. Any smoke-free workplace, restaurant, or bar law	State/region	Various timings	Variable	Children aged 0 to 17 years	Primary: Asthma hospital admissions<br><br>Secondary: Child mortality	ICD−9 and ICD−10 codes	Multiple Cause of Death database, Medicare claims, and the Nationwide Inpatient	Children aged ≤ 17 years	Moderate

											Survey		
Simón (2017)[33]	Spain	46·56	ITS	1st smoke-free law: Complete smoke-free workplaces and partial smoke-free restaurants and bars<br><br>2nd smoke-free law: Public places and workplaces (including restaurants and bars)	National	1st smoke-free law: 01/01/2006<br><br>2nd smoke-free law: 01/01/2011	1st smoke-free law: None<br><br>2nd smoke-free law: Complete smoke-free workplaces and partial smoke-free restaurants and bars	Newborns	Primary: PTB<br><br>Secondary: LBW; SGA	PTB: GA<37 wk<br><br>LBW: BW<2500g; SGA: BW<p10 for GA	Spanish National Statistics Institute Birth Registry	Live births	Moderate
Vicedo-Cabrera (2016)[34]	Switzerland (multiple regions)	8·08	ITS	Public places and workplaces (including restaurants and bars), with several exceptions in the hospitality sector¶¶	National /cantonal	Federal level: 01/05/2010<br><br>Cantonal level: various timings	Variable (12 out of 26 cantons had previously introduced their own laws with a higher level of protection for hospitality workers)	Neonates	Primary: PTB<br><br>Secondary: None	PTB: GA<37 wk	The Swiss Federal Office of Statistics	Singleton live births in Switzerland whose mothers resided in the country, GA 22−42 wk	Low
Vicedo-Cabrera (2017)[35]	Switzerland (multiple regions)	8·08	ITS	Public places and workplaces (including restaurants and bars), with several exceptions in the hospitality sector¶¶	National /cantonal	Federal level: 01/05/2010<br><br>Cantonal level: various timings	Variable (12 out of 26 cantons had previously introduced their own laws with a higher level of protection for hospitality workers)	Children aged 0 to 15 years	Primary: Emergency hospital admissions for respiratory infections<br><br>Secondary: Infant mortality	RTI: ICD-10 codes J00–22<br><br>Infant mortality: death before 1 year of age	Health Registry of the Swiss Federal Statistical Office (Bundesamt für Statistik)	Children aged ≤15 years	Low

**MPOWER: Raise taxes on tobacco**													
Study (year)	Country (region)	Area pop (x10^{6})*	Study design	Intervention				Age of participants	Outcome				Summary risk of bias†
				Details	Level	Date	Previous intervention in place		Eligible outcomes	Definition	Data source	Inclusion criteria	
Adams (2013)[1]	USA (multiple states)	197·73	CITS	Real cigarette price (in 2008 USD)	State	Various timings	NA	Neonates	Primary: None<br><br>Secondary: BW; GA	BW in g; GA in wk	Pregnancy Risk Assessment Monitoring System; birth certificates	Singleton live births	Low

Bhai (2015)[36]	USA (multiple states)	Unknown	CITS	Cigarette excise tax increase (in 2007 USD)	State	Various timings	NA	Children aged 0 to 17 years	Primary: None<br><br>Secondary: Asthma prevalence	Self-reported asthma prevalence	National Survey of Children's Health	Children aged ≤ 17 years	Low
Briggs (2012)[9]	USA (multiple states)	Unknown	CITS	Cigarette excise tax increase in USD	State	Various timings	NA	Neonates	Primary:<br><br>Secondary: GA, BW, LBW	GA in wk; BW in g; LBW: BW<2500g	Natality Detail File 1989 to 2004; National Vital Statistics	Singleton live births, mothers living in counties with population >100,000	Moderate
Evans (1999)[37]	USA (multiple states)	Unknown	CITS	Cigarette excise tax increase in USD	State	Various timings	NA	Neonates	Primary: None<br><br>Secondary: BW; LBW; VLWB	BW in g; LBW: BW<2500g; VLBW: BW<1500g	Natality Detail File 1989 to 1992	Singleton live births, maternal age 15−44 years	Low
Hawkins (2014)[19]	USA (multiple states)	139·68	ITS	Cigarette excise tax increase (in December 2010 USD)	State	Various timings	NA	Neonates	Primary: PTB<br><br>Secondary: BW; LBW; SGA	PTB: GA<37 wk<br><br>BW in g; LBW: BW<2500g; SGA: GA<p10 for sex	US Natality Files (National Vital Statistics System (2000−2004); Public Health Statistics and Information Systems (2005−2010))	Singleton live births, GA 30−44 wk, maternal age 18−50 years (with complete data on smoking, maternal nativity, education, or BW), BW consistent with GA	Low
Hawkins (2016)[20]	USA (Massachusetts, New Hampshire, Vermont)	8·70	ITS	Cigarette excise tax increase:<br><br>Massachusetts:<br>1. USD 0·76 -> 1·51<br>2. USD 1·51 -> 2·51<br><br>New Hampshire:<br>1. USD 0·52 -> 0·80<br>2. USD 0·80 -> 1·08<br>3. USD 1·08 -> 1·33<br>4. USD 1·33 -> 1·78<br><br>Vermont:	State	Massachusetts:<br>1. 25/07/2002<br>2. 01/07/2008<br><br>New Hampshire:<br>1. 01/07/2005<br>2. 01/07/2007<br>3. 01/10/2008<br>4. 01/07/2009<br><br>Vermont:<br>1. 01/07/2002<br>2. 01/07/2003	NA	Children aged 0 to 17 years	Primary: Asthma ED visits; LRTI ED visits; URTI ED visits<br><br>Secondary: None	ICD−9-CM codes. Asthma: 493; LRTI: 466, 480−488; URTI: 460−465	Massachusetts: The Massachusetts Centre for Health Information and Analysis<br><br>New Hampshire: New Hampshire Department	Children aged ≤ 17 years	Low

				1. USD 0·44 -> 0·93<br>2. USD 0·93 -> 1·19<br>3. USD 1·19 -> 1·79<br>4. USD 1·79 -> 1·99<br>5. USD 1·99 -> 2·24		3. 01/07/2006<br>4. 01/07/2008<br>5. 01/07/2009					of Health and Human Services<br><br>Vermont: Green Mountain Care Board		
Landers (2014)[22]	USA (multiple states)	110·73	CITS	Cigarette excise tax increase in USD	State	Various timings	NA	Children aged 0 to 19 years	Primary: Asthma discharges<br><br>Secondary: None	Asthma discharge rates	Healthcare Cost and Utilization Project	Children aged ≤ 19 years	Moderate
Ma (2013)[38]	USA (Pennsylvania)	12·79	ITS	1. USD 0·69 cigarette excise tax increase<br>2. USD 0·35 cigarette excise tax increase	State	1. 15/07/2002<br>2. 07/01/2004	NA	Children aged 0 to 18 years	Primary: Asthma hospitalisations<br><br>Secondary: None	Asthma quarterly discharge rates	Pennsylvania Health Care Cost Containment Council hospital discharge database	Children aged ≤ 18 years	Moderate
Markowitz (2013)[26]	USA (multiple states)	Unclear (29 states and New York City)	CITS	1. Cigarette excise tax increase (in 2008 USD)<br><br>2. Cigarette price increase (in 2008 USD)	State/local	Various timings	NA	Neonates	Primary: PTB<br><br>Secondary: BW; EPTB; GA; LBW; VLBW; VPTB	PTB: GA<37 wk<br><br>BW in g;<br>EPTB: GA 20−27 wk;<br>GA in wk;<br>LBW: BW 1500−2499g;<br>VLBW: BW<1500g;<br>VTPB: GA 28−33 wk	Pregnancy Risk Assessment Monitoring System	Singleton live births	Low
Patrick (2016)[39]	USA (all states)	318·9	ITS	1. Cigarette excise tax increase (in 2010 USD)<br><br>2. Cigarette price increase (in 2010 USD)	State + federal	Various timings	NA	Infants aged 0 to 12 months	Primary: None<br><br>Secondary: Infant mortality	Infant mortality: death before 1 year of age	Centers for Disease Control and Prevention Wide-Ranging Online Data for Epidemiologic Research system	Infants aged ≤ 12 months	Moderate

Sen (2011)[40]	Canada	35·16	CITS	Lagged cumulative federal and provincial excise and sales tax per 200 cigarettes in real Canadian dollars	Province + federal	01/02/1994	NA	Neonates	Primary:<br>None<br><br>Secondary:<br>Fetal death;<br>Infant mortality;<br>LBW	NR	Canadian Socio-economic Information Management	NR	Moderate

MPOWER: Offer help to quit tobacco use (e.g. providing smoking cessation services)

Study (year)	Country (region)	Area pop ($x10^6$)*	Study design	Intervention				Age of participants	Outcome				Summary risk of bias†
				Details	Level	Date	Previous intervention in place		Eligible outcomes	Definition	Data source	Inclusion criteria	
Adams (2013)[1]	USA (multiple states)	197·73	CITS	Medicaid coverage of nicotine replacement therapies, medications, and cessation counselling	State	Various timings	None	Neonates	Primary:<br>None<br><br>Secondary:<br>BW; GA	BW in g; GA in wk	Pregnancy Risk Assessment Monitoring System; birth certificates	Singleton live births	Low
Hawkins (2016)[20]	USA (Massachusetts)	6·75	ITS	Health reform legislation which provided counselling for smoking cessation, and tobacco cessation treatment to Medicaid recipients	State	01/07/2006	None	Children aged 0 to 17 years	Primary:<br>Asthma ED visits; LRTI ED visits; URTI ED visits<br><br>Secondary:<br>None	ICD−9-CM codes. Asthma: 493;<br>LRTI: 466, 480−488;<br>URTI: 460−465	Massachusetts: The Massachusetts Centre for Health Information and Analysis<br><br>New Hampshire: New Hampshire Department of Health and Human Services<br><br>Vermont: Green Mountain Care Board	Children aged ≤ 17 years	Low
Jarlenski (2014)[41]	USA (multiple states)	69·89	CITS	State adoption of one of two optional Medicaid enrolment policies, allowing more low-income pregnant women to receive prenatal care,	State	Various timings	None for the target population (low-income women not eligible for	Neonates	Primary:<br>PTB<br><br>Secondary:<br>SGA	PTB: GA<37 wk<br><br>SGA: BW <p10 for GA	Pregnancy Risk Assessment Monitoring System	Singleton live births, maternal age 19−44 years, maternal smoking during 3 months before	Low

				including smoking cessation services (presumptive eligibility and the unborn child option)***			Medicaid enrolment)<br><br>Note: Low-income pregnant women who were already eligible for Medicaid always had access to this prenatal care.					conception, mothers eligible for Medicaid during pregnancy in their state in the year the pregnancy began	

BW=birth weight; CITS=controlled interrupted time series; CM codes=clinical modification codes; ED=emergency department; ELBW=extremely low birth weight; EPTB=extremely preterm birth; g=grams; GA=gestational age; GP=general practitioner; ICD=International Classification of Diseases; ITS=interrupted time series; LBW=low birth weight; LRTI=lower respiratory tract infection; pop=population; NA=not applicable; p=percentile; PTB=preterm birth; RTI=respiratory tract infection; SD=standard deviation; SGA=small for gestational age; URTI=upper respiratory tract infection; USD=US dollars; wk=weeks; VLBW=very low birth weight; VPTB=very preterm birth; VSGA=very small for gestational age; y=year

* Area population statistics were obtained from the most recent possible estimates.
† See Appendix pp 32, 33 for details.
‡ Qualified: all workplaces/restaurants/bars smoke-free with two possible general exceptions: (1) workplaces/restaurants with a specified number of employees/seats or fewer; (2) smoking permitted in enclosed, separately ventilated smoking rooms.
§ Some coverage: some coverage for workplaces, restaurants, and bars, but less than that of the 100% smoke-free and qualified categories.
|| Different states passed different 100% smoke-free laws: workplaces, restaurants, and bars (eight states), restaurants and bars (two states), workplaces and restaurants (one state), and workplaces (one state).
¶ Day enforcement began, smoke-free law implemented December 2006
** Both smoke-free laws were accompanied by a tobacco tax increase and mass-media campaign.
†† Exceptions to this smoking smoke-free law were: hotels, bars and restaurants, sports, arts and culture venues, amusement arcades, tobacconist shops, international passenger transport, private spaces, open air, and designated areas for smoking within each facility.
‡‡ The smoking smoke-free law now included hospitality venues: hotels, bars and restaurants, sports, art and culture venues, amusement arcades, tobacconist shops, and international passenger transport. Designated smoking areas within each facility were still allowed.
§§ Congenital anomalies were defined as: cardiovascular/heart defects, musculoskeletal defects, missing/extra digits, limb reduction defects, clubfoot, craniosynostosis, facial defects, eye defects, orofacial clefts, gastrointestinal defects, gastroschizis, anal atresia, and abdominal wall defects
|||| Including, but not limited to: restaurants, bars, bowling alleys, bingo halls, convenience stores, laundromats, and other business open to the public.
¶¶ Authorised smoking in establishments <80m^2 and designated smoking areas in larger establishments.
*** Presumptive eligibility: low-income pregnant women are presumed to be eligible for Medicaid, so they can receive care (including smoking cessation services) while their Medicaid applications are still pending. The unborn-child option: the state can consider a fetus a “targeted low-income child”, allowing coverage of prenatal care (including smoking cessation services) and delivery to low-income pregnant women, even if they cannot provide documentation of citizenship or residency.

Table S3: Characteristics of included non-EPOC studies

Study (year)	Country (region)	Study Design	Date of Intervention	Intervention details / level	Age of participants	Inclusion criteria	Data source	Eligible outcomes	Population at risk			Direct change in events (%): step change (95% CI)	Sustained change in events per year (%): slope change (95% CI)	Summary of findings
									Total	Before intervention	After intervention			
**Smoke-free legislation (P)**														
Bianchi (2011)[42]	Italy (Lombardy)	UBA	10 Jan 2005	Unknown.<br><br>Region (local)	0 to 14 years	Children aged ≤ 14 years	Administrative Regional Database	Primary: Asthma exacerbations requiring hospital attendance<br><br>Secondary: None	Unknown	Unknown	Unknown	–30·7% (–22·8 to –38·6)	N/A	Smoke-free legislation was associated with a 30·7% reduction in the rate of hospitalisations for childhood asthma.
Dove (2011)[43]	USA (multiple regions)	UBA	Various timings	At least one smoke-free workplace, restaurant, or bar law at county or state level.	3 to 15 years	Non-smoking children* aged 3–15 years.	National Health and Nutrition Examination Survey	Primary: Asthma exacerbations requiring hospital attendance<br><br>Secondary: None	8,800	6,573 (without smoke-free law)	2,227 (with smoke-free law)	Unadjusted †: OR 0·77 (0·43 to 1·39)<br>Adjusted ‡: OR 0·55 (0·27 to 1·13)	N/A	Smoke-free laws were associated with a reduction in asthma emergency-department visits.
Kabir (2009)[44]	Ireland (Dublin)	UBA	Mar 2004	Comprehensive workplace smoke-free law at country (national) level.	Neonates	Singleton live-birth babies with complete birthweight data.	Euroking K2 maternity systems	Primary: PTB	15,241	7,593	7,648	OR 0·75 (0·59 to 0·96)	N/A	Significant 25% decline in preterm births after smoke-free law.
								Secondary: LBW	15,241	7,593	7,648	OR 1·43 (1·10 to 1·85)	N/A	Significant 43% increase in LBW after the smoke-free law.

CI=confidence interval; LBW=low birth weight; OR=odds ratio; PTB=preterm birth; UBA=uncontrolled before-after study.

* Defined by both cotinine levels and self-reported smoking status.
† Unadjusted model: No covariates adjusted for.
‡ Adjusted model: Survey cycle, gender, age, race, ratio of income to poverty, region, health insurance, mother's age at birth, mother's smoking status during pregnancy, low birth weight, BMI, and household size.

Table S4: Model specifications of included studies

First author (year)	Controls	Time frames*			Time points		Model description	
		Pre-intervention	Post-intervention	Interval	Points pre-intervention	Points post-intervention	Type of model	Adjusted model
**Protect people from tobacco smoke**								
Adams (2013)[1]	Regions and time points without ban	Variable	Variable	Month	Variable	Variable	Ordinary least squares regression	Underlying trend, maternal race/ethnicity, maternal age, marital status, maternal education, prior birth and outcome, alcohol consumption during pregnancy, experienced physical abuse by partner or spouse, number of stressful life factors mother experienced during 12 months before delivery, intendedness of pregnancy, infant sex, trimester prenatal care initiated, presence of diabetes or hypertension, state level real income, state cigarette price, increased Medicaid coverage of tobacco cessation services
Amaral (2009)[2]	Regions and time-points without ban	State-wide: 01/07/1988 to 31/12/1994<br><br>Local: variable	State-wide: 01/01/1995 to 31/12/1999<br><br>Local: variable	Quarter (3 months)	State-wide: 26<br><br>Local: variable	State-wide: 20<br><br>Local: variable	Difference in differences regression	Sex, parity, plurality, maternal age, maternal education, maternal race/ethnicity, city, and underlying time trend
Bakolis (2016)[3]	Pre-ban	± 1 month: 15/05/2007 to 15/06/2007<br>± 2 months: 15/04/2007 to 15/06/2007<br>± 3 months: 15/03/2007 to 15/06/2007<br>± 5 months: 15/01/2007 to 15/06/2007	± 1 month: 15/07/2007 to 15/08/2007<br>± 2 months: 15/07/2007 to 15/09/2007<br>± 3 months: 15/07/2007 to 15/10/2007<br>± 5 months: 15/07/2007 to 15/12/2007	NA	NA	NA	Fuzzy regression discontinuity	Maternal age, seasonality, and underlying trend†
Bartholomew (2016)[4]	Regions and time-points without ban	Variable	Variable	Month	Variable	Variable	Individual-level weighted least squares regression	Maternal age, race, maternal education, marital status, parity, month prenatal care began, gestational weight gain, air pollution‡, county, county-specific trends, and seasonality
Been, Mackay (2015)[5]	Pre-ban	01/01/1995 to 30/06/2007	01/07/2007 to 31/12/2011	Month	150	54	Logistic regression using individual-level data	Underlying trend§, maternal age, maternal marital status, sex, SES, region, urbanisation level, birth weight, and seasonality

Been, Millett (2015)[6]	Pre-ban	01/01/2001 to 30/06/2007	01/07/2007 to 31/12/2012	Month	78	66	Negative binomial regression	Age, sex, region, urbanisation, SES, seasonality, underlying trend, and autocorrelation
Been, Szatkowski (2015)[7]	Pre-ban	England: 01/01/1997 to 30/06/2007<br><br>Scotland: 01/01/1997 to 31/03/2006<br><br>Wales: 01/01/1997 to 31/03/2007<br><br>Northern-Ireland: 01/01/1997 to 30/04/2007	England: 01/07/2007 to 31/12/2012<br><br>Scotland: 01/04/2006 to 31/12/2012<br><br>Wales: 01/04/2007 to 31/12/2012<br><br>Northern-Ireland: 01/05/2007 to 31/12/2012	Month	England: 126<br><br>Scotland: 111<br><br>Wales: 123<br><br>Northern-Ireland: 124	England: 66<br><br>Scotland: 81<br><br>Wales: 69<br><br>Northern-Ireland: 68	Poisson generalised additive mixed models	Underlying trend, ambient temperature, school holidays, pandemic influenza, variations in the number of days in a month, number of days GP practices were open, seasonality, and autocorrelation
Bharadwaj (2014)[8]	Babies born to mothers working in shops	01/01/2004 to 31/05/2004	01/11/2004 to 31/03/2005	Month	5	5	Difference in differences regression	Maternal income, maternal age, maternal education, maternal working hours, parity, singleton status, county, seasonality, autocorrelation
Briggs (2009)[9]	Regions and time-points without ban	Variable	Variable	Month	Variable	Variable	Difference in differences regression within local-level fixed effects models	Underlying trend, average real price of cigarettes, maternal, infant, and birth characteristics
Ciaccio (2016)[10]	Pre-ban within each region	Variable (3 years)	Variable (3 years)	Month	36	36	Negative binomial regression	Underlying trend, seasonality, sex, race, payer source, age of admission

Cox (2013)[11]	Pre-ban	Ban 1 (public places and workplace): 01/01/2002 to 31/12/2005<br><br>Ban 2 (including restaurants): 01/01/2002 to 31/12/2006<br><br>Ban 3 (including bars): 01/01/2002 to 31/12/2009	Ban 1: 01/01/2006 to 31/12/2011<br><br>Ban 2: 01/01/2007 to 31/12/2011<br><br>Ban 3: 01/01/2010 to 31/12/2011	Month	Ban 1: 48<br><br>Ban 2: 60<br><br>Ban 3: 96	Ban 1: 72<br><br>Ban 2: 60<br><br>Ban 3: 24	Logistic regression	Sex, maternal age, parity, SES, urbanisation level, month, day, holidays, influenza epidemics, pollution, ambient temperature, underlying trend, seasonality
Croghan (2015)[12]	Pre-ban	01/01/2005 to 30/04/2007	01/10/2007 to 31/12/2009	Month	28	27	Segmented Poisson regression	Age, sex, underlying trend
Galán (2017)[13]	Pre-ban periods	1st smoke-free law: 01/01/2003 – 31/12/2005<br><br>2nd smoke-free law: 01/01/2006 – 31/12/2010	1st smoke-free law: 01/01/2006 – 31/12/2010<br><br>2nd smoke-free law: 01/01/2011 – 30/11/2012	Day	1st smoke-free law: 1095<br><br>2nd smoke-free law: 1825	1st smoke-free law: 1095<br><br>2nd smoke-free law: 730	Poisson additive regression at province level / Random-effects meta-analysis at national level	Underlying trend, seasonality, day of the week, temperature, influenza epidemics, acute respiratory infections, and pollen counts
Gao (2016)[14]	Regions and time-points without ban	Variable	Variable	Quarter (3 months)	Variable	Variable	Ordinary least squares regression	Maternal and infant characteristics, underlying trend, state-level cigarette tax rates, and other policies that might affect infant birth outcomes.
Gaudreau (2013)[15]	Pre-ban	01/04/1995 to 31/05/2003	01/06/2003 to 31/12/2008	Month	98	67	ARIMA	Seasonality, autocorrelation
Hade (2011)[16]	Pre-ban	01/01/2006 to 02/05/2007	03/05/2007 to 31/12/2009	Month	16	32	ARIMA / GLM with Poisson distribution / Logistic regression	In logistic regression model: maternal age, race, ethnicity, marital status, insurance status, seasonality, autocorrelation
Hajdu (2017)[17]	Working mothers unexposed to cigarette smoke in their workplace during the period before implementation of smoke-free legislation with similar personal characteristics as the	NR	NR	Month	NR	NR	Ordinary least squares regression	Underlying trend, seasonality, maternal age, marital status, maternal education, number of pregnancies/live births/abortions, number of days between current and previous live birth, sex of newborn, paternal age, paternal education, and paternal employment status

	intervention group							
Hankins (2016)[18]	Regions and time-points without ban	Variable	Variable	Quarter (3 months)	Variable	Variable	Fixed effects ordinary least squares regression	Underlying trends, marital status, maternal education, maternal age, ethnicity, state level average cigarette prices (in 2009 USD), county, and quarter
Hawkins (2014)[19]	Regions and time-points without ban	Variable	Variable	Month	Variable	Variable	2 step modelling approach:<br><br>1. Probit regression for probability of maternal smoking during pregnancy<br><br>2. Linear/probit regression for outcomes, conditioned on probability of maternal smoking during pregnancy	Maternal race/ethnicity, maternal race/ethnicity × tax, maternal education, maternal education × tax, marital status, country of birth, number of live births, prenatal care, maternal race/ethnicity × maternal age, maternal race/ethnicity × state, maternal race/ethnicity × year; conditioned on probability of maternal smoking
Hawkins (2016)[20]	Regions and time-points without ban	Variable	Variable	Month	Variable	Variable	Negative binomial regression	Municipality, seasonality, population size, % of population covered by Medicaid, age, state smoke-free legislation × age
Kabir (2013)[21]	Pre-ban	01/01/1999 to 30/04/2004	01/05/2004 to 31/12/2008	Month	64	56	Mixed models	Sex, maternal smoking¶, maternal age, parity, marital status, antenatal care, parental occupation, regional clustering, underlying trend. Tested for 1st order autocorrelation: not detected
Landers (2014)[22]	Regions and time-points without ban	Variable	Variable	Quarter (3 months)	Variable	Variable	Ordinary least squares regression	Cigarette tax, smoking prevalence, asthma prevalence, urbanisation level, SES‖, ethnicity**, percentage of insured people, presence of (teaching) hospital in county, number of primary care physicians per 10,000 residents, year and state, and seasonality

Lee (2016)[23]	Pre-ban	01/01/2004 to 31/12/2006	01-01-2007 to 31-12-2012	Day	NR	NR	Negative binomial regression	Meteorological factors††, multiple pollutants‡‡, daily hospital admissions for influenza, holidays, day of the week, day, and seasonality
Mackay (2010)[24]	Pre-ban	01/01/2000 to 25/03/2006	26/03/2006 to 31/10/2009	Month	75	43	Negative binomial regression	Age, sex, SES, urbanisation level, region, underlying trend, seasonality
Mackay (2012)[25]	Pre-ban	01/01/1996 to 31/12/2005	01/01/2006 to 31/12/2009	Week	520	208	Logistic regression	Sex, maternal age, SES, week, previous abortions, parity, underlying trend, seasonality
Markowitz (2013)[26]	Regions and time-points without ban	Variable	Variable	NR	Variable	Variable	Reduced form model	Cigarette tax, cigarette price, real estate income per capita, maternal ethnicity, maternal education, marital status, prior birth complications, mistimed birth, unwanted birth, abuse, stressors, alcohol use, insurance, sex, prenatal care, diabetes, hypertension, indicator variables for missing values, state, year, state-specific underlying time trends
McKinnon (2015)[27]	Pre-ban	01/01/2003 to 31/05/2006	01/06/2006 to 31/12/2010	Month	41	55	Linear and logistic regression	Maternal age, sex, parity, marital status, maternal nativity, material deprivation§§, underlying trend, and month of birth
Millett (2013)[28]	Pre-ban	01/04/2002 to 30/06/2007	01/07/2007 to 30/112010	Month	63	41	Negative binomial regression	Age, sex, urbanisation level, SES, region, month, underlying trend, seasonality
Page (2012)[29]	City without smoking ban	01/04/2001 to 01/07/2003	01/04/2004 to 01/07/2006	Month	27	27	Logistic regression	Low BW: sex, maternal age, race, ethnicity, maternal education, marital status, maternal smoking, maternal alcohol consumption, parity, maternal hypertension, pre-pregnancy hypertension, caesarean section, previous preterm or SGA birth, underlying trend.<br>Preterm birth: as above, plus: maternal diabetes, maternal anaemia, pregnancy complications, BW
Peelen (2016)[30]	Pre-ban periods	1st ban: 01/01/2000 to 31/12/2003<br><br>2nd ban: 01/01/2000 to 30/05/2008	1st ban: 01/01/2004 to 31/12/2011<br><br>2nd ban: 01/07/2008 to 31/12/2011	Month	1st ban: 47<br><br>2nd ban: 101	1st ban: 97<br><br>2nd ban: 43	Logistic regression	Underlying trends, month, maternal age, ethnicity, SES, urbanisation level, parity, preeclampsia, sex, caesarean section, and seasonality

Rayens (2008)[31]	Pre-ban	01/01/2001 to 26/04/2004	27/04/2004 to 31/12/2006	Month	40	32	Negative binomial regression¶¶	Age, sex, age*sex, underlying trend, seasonality
Shetty (2011)[32]	Regions and time points without bans	Variable	Variable	Year	Variable	Variable	Fixed effects logistic regression	Underlying trend, state cigarette tax, region
Simón (2017)[33]	Pre-ban periods	1st smoke-free law: 01/01/2000 – 31/12/2005<br><br>2nd smoke-free law: 01/01/2006 – 31/12/2010	1st smoke-free law: 01/01/2006 – 31/12/2010<br><br>2nd smoke-free law: 01/01/2011 – 31/12/2013	Month	1st smoke-free law: 60<br><br>2nd smoke-free law: 48	1st smoke-free law: 48<br><br>2nd smoke-free law: 36	Segmented Poisson regression	Maternal age, SES, maternal region of residence, place of delivery, health professional-assisted birth, multiple birth, annual prevalence of tobacco consumption during pregnancy
Vicedo-Cabrera (2016)[34]	Pre-ban within each canton	Variable	Variable	Week	Variable	Variable	Quasi-Poisson regression at canton level / Random-effects meta-analysis at national level	Underlying trend, seasonality, pregnancy-outcome specific offset
Vicedo-Cabrera (2017)[35]	Pre-ban within each canton	Variable (2 years)	Variable (2 years)	Month	24	24	Quasi-Poisson regression at canton level / Random-effects meta-analysis at national level	Underlying trend, seasonality, influenza peaks, number of days of the month
**Raise taxes on tobacco**								
Adams (2013)[1]	Regions and time points before changes in cigarette price	Variable	Variable	Month	Variable	Variable	Ordinary least squares regression	Underlying trend, maternal race/ethnicity, maternal age, marital status, maternal education, prior birth and outcome, alcohol consumption during pregnancy, experienced physical abuse by partner or spouse, number of stressful life factors mother experienced during 12 months before delivery, intendedness of pregnancy, infant sex, trimester prenatal care initiated, presence of diabetes or hypertension, state level real income, increased Medicaid

								coverage of tobacco cessation services, indicator of a state ban on indoor smoking in all restaurants
Bhai (2015)[36]	Regions and time points before changes in cigarette tax	Variable	Variable	Year	Variable	Variable	Difference in differences	Underlying trend, seasonality, race, gender, local macroeconomic conditions
Evans (1999)[37]	Regions and time points before changes in cigarette tax	Variable	Variable	Month	Variable	Variable	Difference in differences	Underlying trend, state, age, race, sex, parity, marital status, maternal education, adequacy of prenatal care, weight gain during pregnancy
Hawkins (2014)[19]	Regions and time points before changes in cigarette tax	Variable	Variable	Month	Variable	Variable	2 step modelling approach:<br><br>1. Probit regression for probability of maternal smoking during pregnancy<br><br>2. Linear/probit regression for outcomes, conditioned on probability of maternal smoking during pregnancy	Maternal race/ethnicity, maternal education, marital status, country of birth, number of live births, prenatal care, maternal age, state, year

Hawkins (2016)[20]	Regions and time points before changes in cigarette tax	Variable	Variable	Month	Variable	Variable	Negative binomial regression	Municipality, seasonality, population size, % of population covered by Medicaid, age, state smoke-free legislation × age
Landers (2014)[22]	Regions and time points before changes in cigarette tax	Variable	Variable	Quarter (3 months)	Variable	Variable	Difference in differences	Cigarette tax, smoking prevalence, asthma prevalence, urbanisation level, SES‖, ethnicity**, percentage of insured people, presence of (teaching) hospital in county, number of primary care physicians per 10,000 residents, year and state, and seasonality
Ma (2013)[38]	Periods before tax increase	1st period: 01/01/2000 to 30/06/2002<br><br>2nd period: 01/01/2000 to 31/12/2003	1st period: 01/07/2002 to 31/12/2008<br><br>2nd period: 01/01/2004 to 31/12/2008	Quarter (3 months)	1st period: 10<br>2nd period: 16	1st period: 26<br>2nd period: 20	Segmented regression	Autocorrelation
Markowitz (2013)[26]	Regions and time points before changes in cigarette tax	Variable	Variable	NR	Variable	Variable	Reduced form model	Cigarette tax, cigarette price, real estate income per capita, maternal ethnicity, maternal education, marital status, prior birth complications, mistimed birth, unwanted birth, abuse, stressors, alcohol use, insurance, sex, prenatal care, diabetes, hypertension, indicator variables for missing values, state, year, state-specific underlying time trends
Patrick (2016)[39]	Regions and time points before changes in cigarette tax	Variable	Variable	Year	Variable	Variable	Linear regression	Underlying trend, educational attainment, mean inflation-adjusted per-capita income, and state random effects

Sen (2011)[40]	Regions and time points before changes in cigarette tax	Variable	Variable	Month	Variable	Variable	Generalised least squares regression and ordinary least squares regression	Underlying trend, province, real beer prices, minimum drinking age, number of physicians per 1,000 individuals, real government health expenditures, provincial unemployment rates
**Offer help to quit tobacco use**								
Adams (2013)[1]	Regions and time points before increased Medicaid coverage of tobacco cessation services	Variable	Variable	Month	Variable	Variable	Ordinary least squares regression	Underlying trend, maternal race/ethnicity, maternal age, marital status, maternal education, prior birth and outcome, alcohol consumption during pregnancy, experienced physical abuse by partner or spouse, number of stressful life factors mother experienced during 12 months before delivery, intendedness of pregnancy, infant sex, trimester prenatal care initiated, presence of diabetes or hypertension, state level real income, state cigarette price, indicator of a state ban on indoor smoking in all restaurants
Hawkins (2016)[20]	Period before health care reform	01/01/2001 to 30/06/2006	01/07/2006 to 30/09/2010	Month	66	51	Negative binomial regression	Municipality and seasonality
Jarlenski (2014)[41]	Regions and time-points without one of the two enrolment policies	Variable	Variable	NR	Variable	Variable	Logistic regression	Individual variables: maternal age, maternal race/ethnicity, maternal education, marital status, number of cigarettes smoked per day before conception, consumption of alcoholic drinks during pregnancy, parity, pregnancy intention, number of stressors experienced during the 12 months before delivery, insurance status before conception, having a preterm birth previously<br><br>State-level variables: smoking prohibition, state excise taxes on cigarettes, state Medicaid income eligibility thresholds, whether a state had a high, medium or low proportion of Medicaid beneficiaries enrolled in a managed care organization

* dd/mm/yyyy format
† Any existing temporal trends that occur every year around the cut-off date were accounted for by dividing the sample into five cohorts centred around the cut-off and adding them into the model (2005, 2006, 2007, 2008, 2009)
‡ Measured by average annual county level of sulphur dioxide
§ Non-linear underlying time trends (via B-splines)
¶ Maternal smoking was based on estimates for ±90% of the population
|| Defined as percentage living in poverty
** Defined as percentage of the population who were non-White
†† Temperature, relative humidity and rainfall
‡‡ particulate matter with aerodynamic diameter <10 µm, sulphur dioxide, nitrogen dioxide and ozone
§§ Quintiles of material deprivation, an area-level index that is estimated for Canadian neighbourhoods based on the postal codes of mothers at the time of birth
¶¶ Additional analyses were performed using 1st-order autoregressive time series model (results not reported)

Table S5: Risk of bias assessment

Study (Year)	Was the intervention independent of other changes?	Was the shape of the intervention effect pre-specified?	Was the intervention unlikely to affect data collection?	Was knowledge of the allocated interventions adequately prevented during the study?	Were incomplete outcome data adequately addressed?	Was the study free from selective outcome reporting?	Was the study free from other risks of bias?	Relevant confounders accounted for?	Summary risk of bias
Adams (2013)[1]	Unclear	Low	Low	Low	Low	Low	Unclear	Low	Low
Amaral (2009)[2]	Unclear	Low	Low	Low	Unclear	Unclear	Moderate	Moderate	High
Bakolis (2016)[3]	Low	Low	Low	Low	Unclear	Low	Low	Low	Low
Bartholomew (2016)[4]	Unclear	Low	Low	Low	Unclear	Low	Low	Low	Low
Been, Mackay (2015)[5]	Low	Low	Low	Low	Low	Low	Low	Low	Low
Been, Millett (2015)[6]	Low	Low	Low	Low	Low	Low	Low	Low	Low
Been, Szatkowski (2015)[7]	Low	Low	Low	Low	Low	Low	Low	Low	Low
Bhai (2015)[36]	Unclear	Low	Low	Low	Unclear	Low	Unclear	Low	Low
Bharadwaj (2014)[8]	Low	Low	Low	Low	Unclear	Low	High	Moderate	Moderate
Briggs (2009)[9]	Low	Low	Low	Low	Unclear	Low	High	Moderate	Moderate
Ciaccio (2016)[10]	Low	Low	Low	Low	Low	Low	Low	Low	Low
Cox (2013)[11]	Low	Low	Low	Low	Low	Low	Low	Moderate	Low
Croghan (2015)[12]	High	Low	Low	Low	Unclear	Low	Unclear	High	High
Evans (1999)[37]	Unclear	Low	Low	Low	Unclear	Low	Unclear	Low	Low
Galán (2017)[13]	Unclear	Low	Low	Low	Unclear	Low	Low	Low	Low
Gao (2017)[14]	Low	Low	Low	Low	Unclear	Low	High	Moderate	Moderate
Gaudreau (2013)[15]	Moderate	Low	Low	Low	Unclear	Low	High	Moderate	Moderate
Hade (2011)[16]	Unclear	Low	Low	Low	Unclear	Low	Moderate	High	Moderate
Hajdu (2017)[17]	Low	Low	Low	Low	Low	Low	Low	Low	Low
Hankins (2016)[18]	Unclear	Unclear	Low	Low	Unclear	Low	Low	Low	Low
Hawkins (2014)[19]	Unclear	Low	Low	Low	Unclear	Low	Low	Low	Low

Hawkins (2016)[20]	Unclear	Low	Low	Low	Unclear	Low	Low	Low	Low
Jarlenski (2014)[41]	Unclear	Low	Low	Low	Unclear	Low	Low	Low	Low
Kabir (2013)[21]	Moderate	Low	Low	Low	Low	Low	Low	Low	Low
Landers (2014)[22]	Unclear	Unclear	Low	Low	Unclear	Low	Low	Moderate	Moderate
Lee (2016)[23]	Moderate	Low	Low	Low	Unclear	Low	Low	Moderate	Moderate
Ma (2013)[38]	Unclear	Low	Low	Low	Unclear	Low	Low	High	Moderate
Mackay (2010)[24]	Low	Low	Low	Low	Low	Low	Low	Moderate	Low
Mackay (2012)[25]	Low	Low	Low	Low	Low	Low	Low	Low	Low
Markowitz (2013)[26]	Unclear	Low	Low	Low	Unclear	Low	Unclear	Low	Low
McKinnon (2015)[27]	Unclear	Low	Low	Low	Unclear	Low	Moderate	Low	Moderate
Millett (2013)[28]	Low	Low	Low	Low	Unclear	Low	Low	Moderate	Moderate
Page (2012)[29]	Unclear	Low	Low	Low	Unclear	Low	Low	Moderate	Moderate
Patrick (2016)[39]	Unclear	Unclear	Low	Low	Low	Low	Moderate	Moderate	Moderate
Peelen (2016)[30]	Low	Low	Low	Low	Low	Low	Low	Low	Low
Rayens (2008)[31]	Moderate	Low	Low	Low	Low	Low	Moderate	Moderate	Moderate
Sen (2011)[40]	Unclear	Unclear	Low	Low	Unclear	Low	Moderate	Moderate	Moderate
Shetty (2011)[32]	Low	Low	Low	Low	Unclear	Low	Low	High	Moderate
Simón (2017)[33]	Unclear	Unclear	Low	Low	Unclear	Unclear	Low	Low	Moderate
Vicedo-Cabrera (2016)[34]	Unclear	Low	Low	Low	Low	Low	Low	Unclear	Low
Vicedo-Cabrera (2017)[35]	Unclear	Low	Low	Low	Low	Low	Low	Unclear	Low

Figure S1: Funnel plot of preterm birth

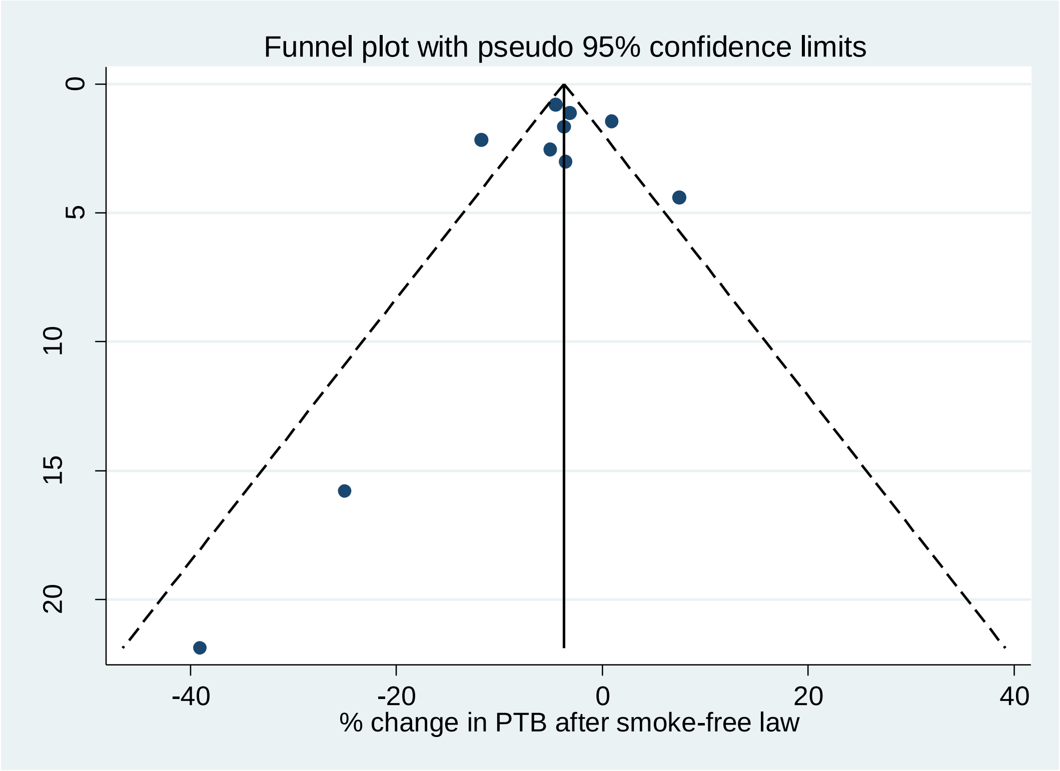

Egger's test for small-study effects: p = 0·65

Figure S2: Sensitivity analysis including non-EPOC studies

A – Preterm birth

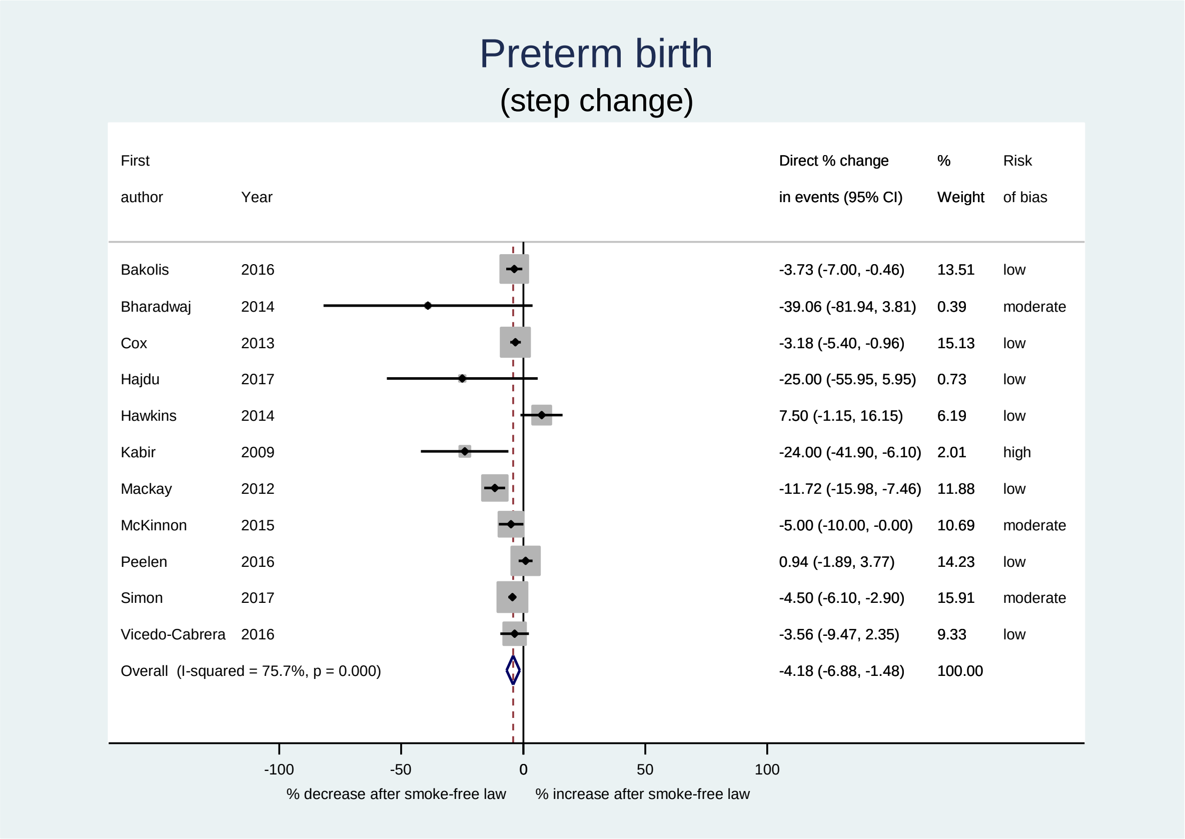

B – Asthma exacerbations requiring hospital attendance

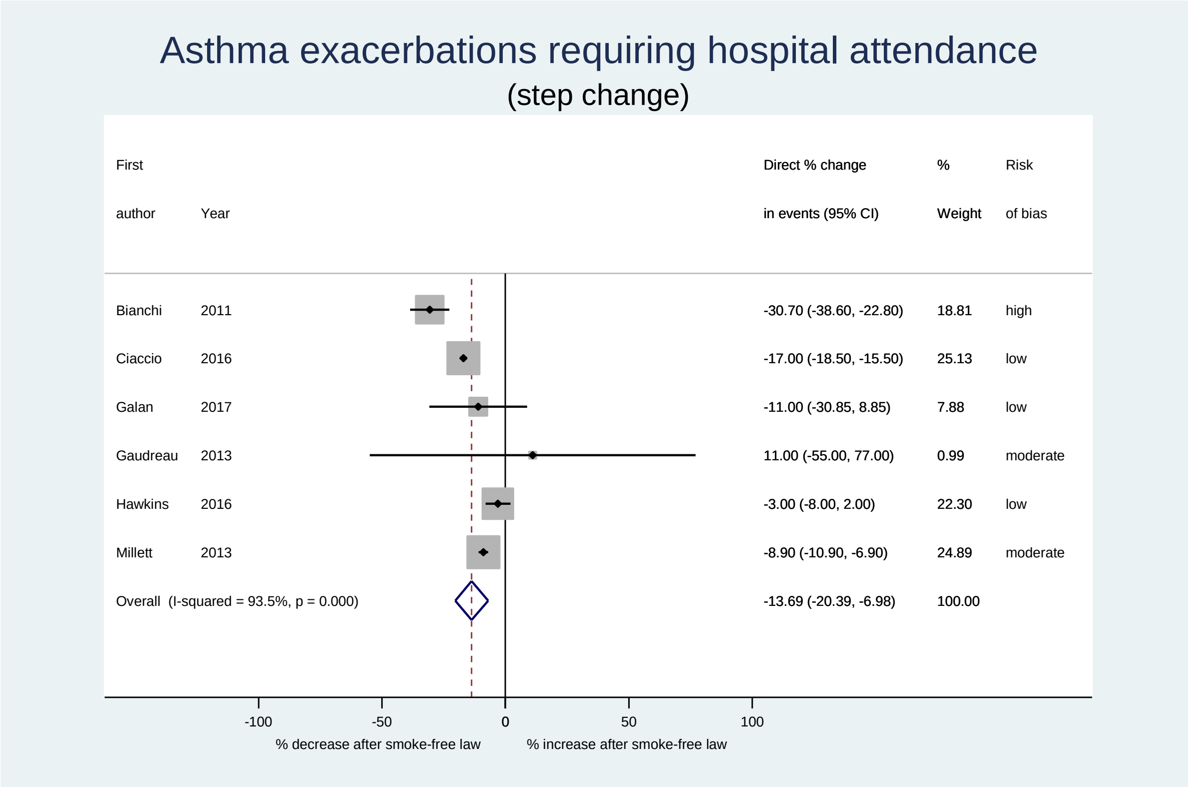

Figure S3: Sensitivity analysis including only studies with low and moderate risk of bias

A – Preterm birth

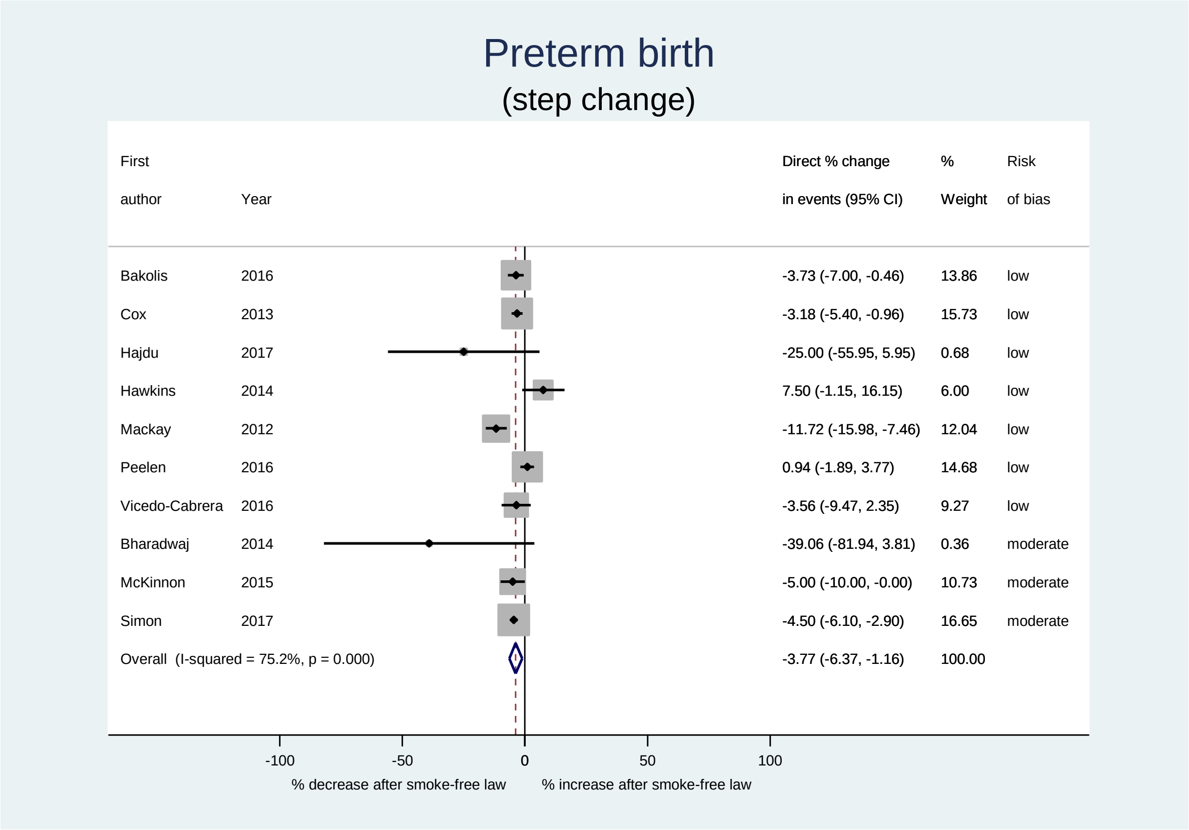

B – Preterm birth (gradual change)

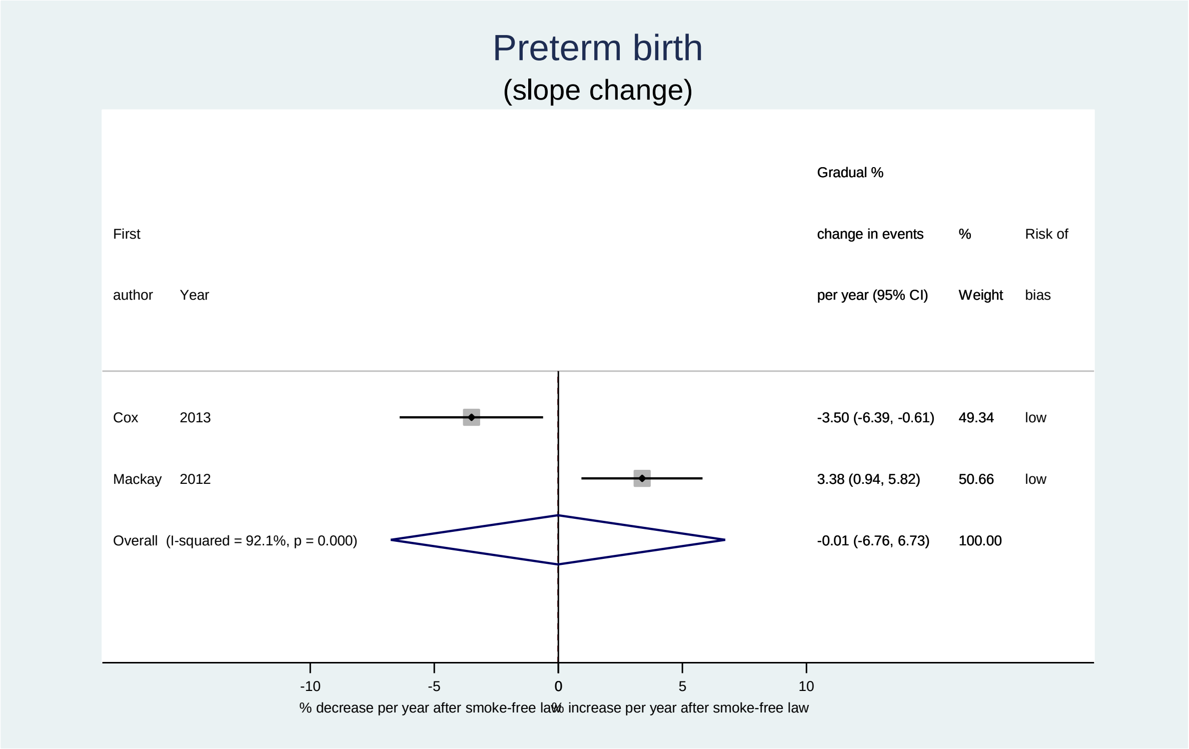

C – Asthma exacerbations requiring hospital attendance

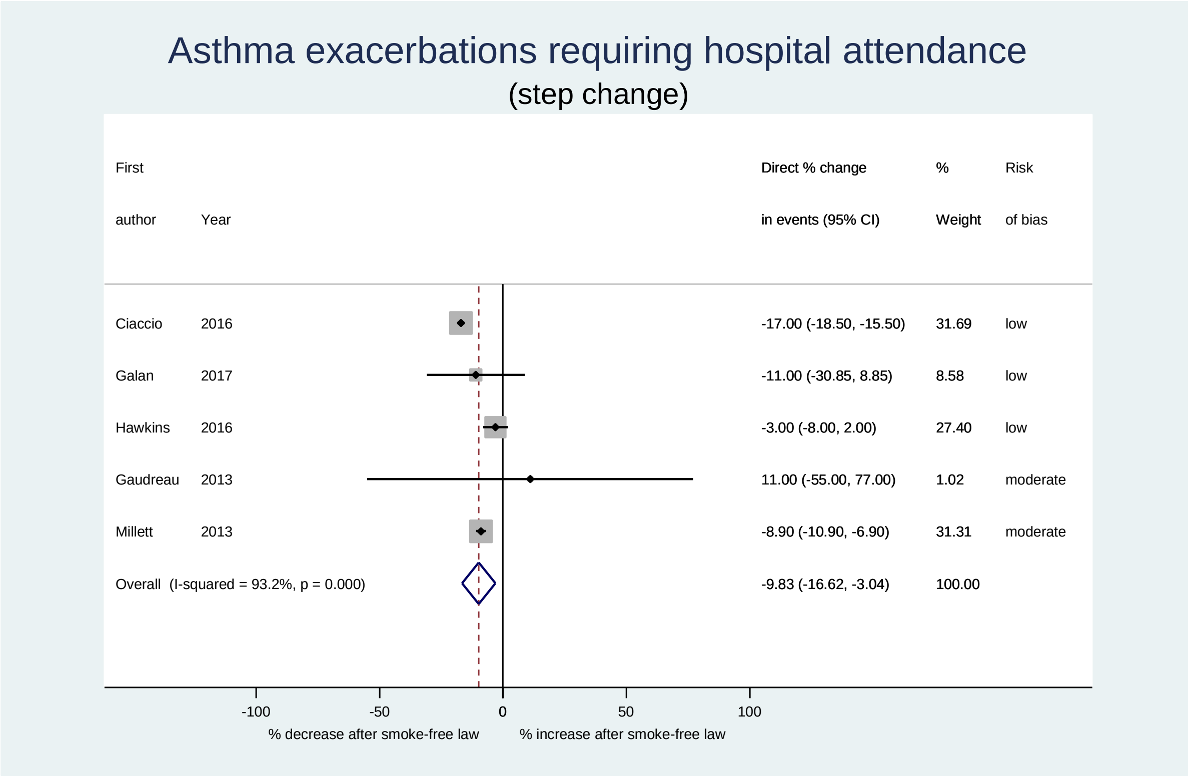

D – Asthma exacerbations requiring hospital attendance (gradual change)

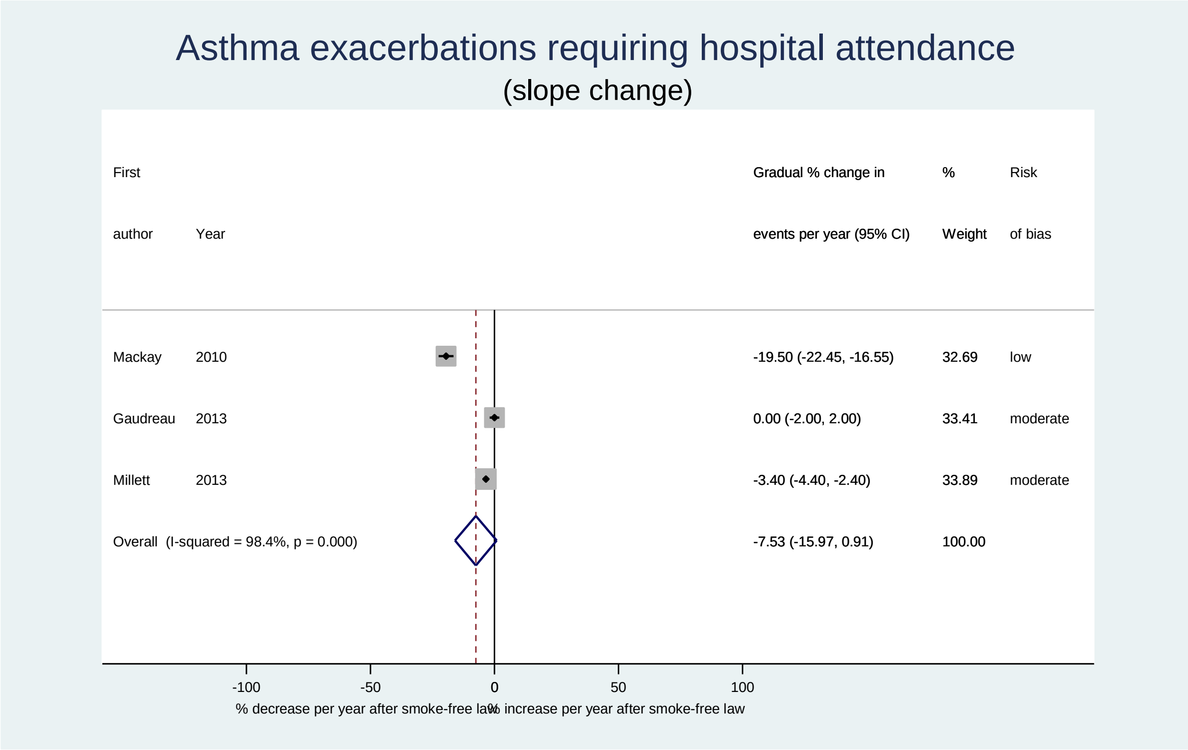

E – Respiratory tract infections requiring hospital attendance

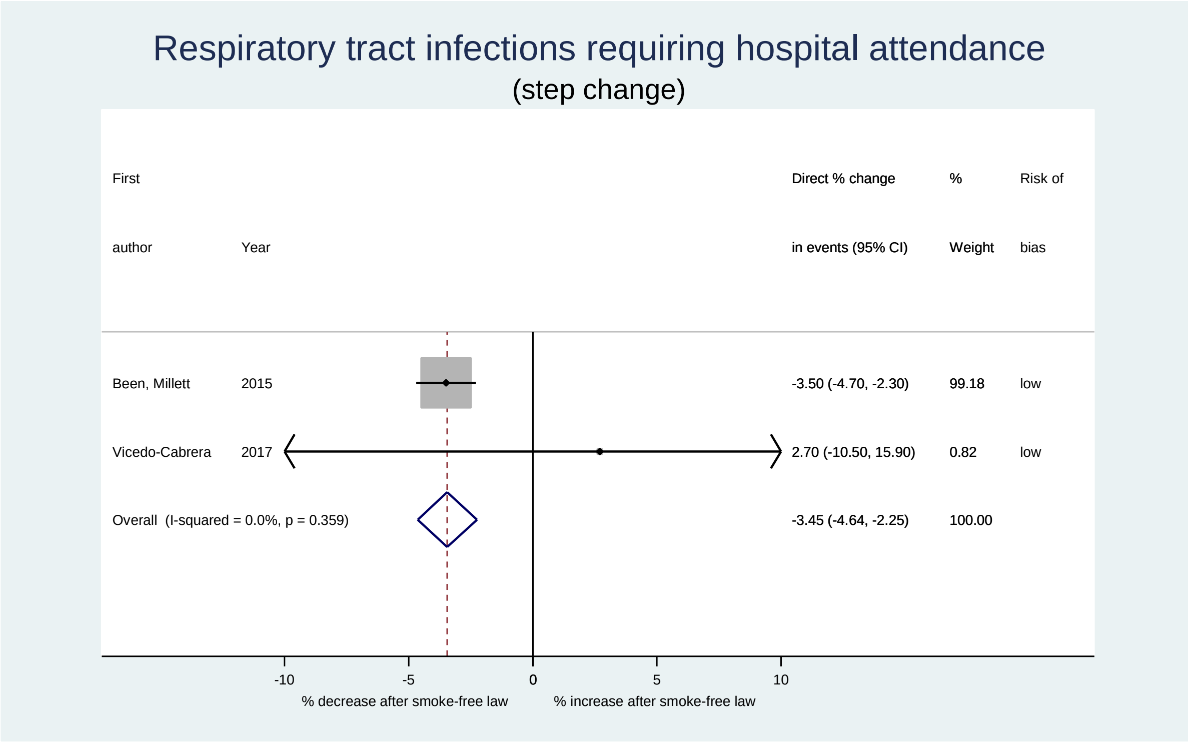

F – Upper respiratory tract infections requiring hospital attendance

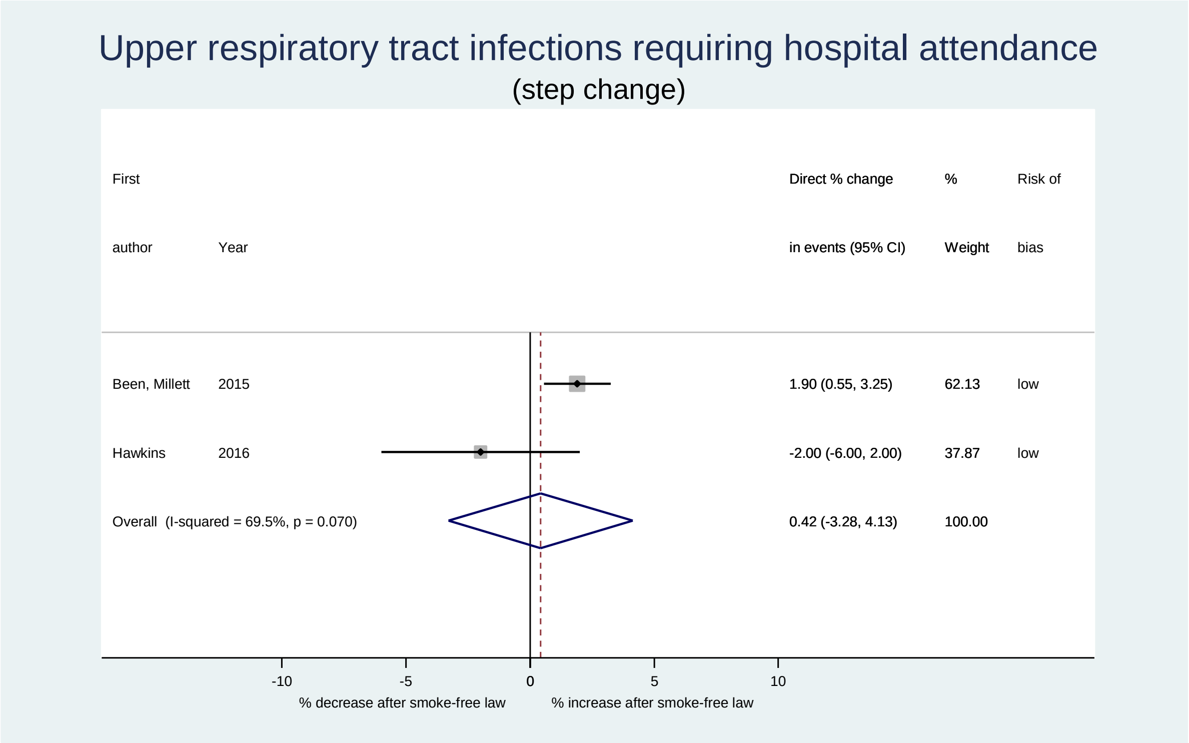

G – Lower respiratory tract infections requiring hospital attendance

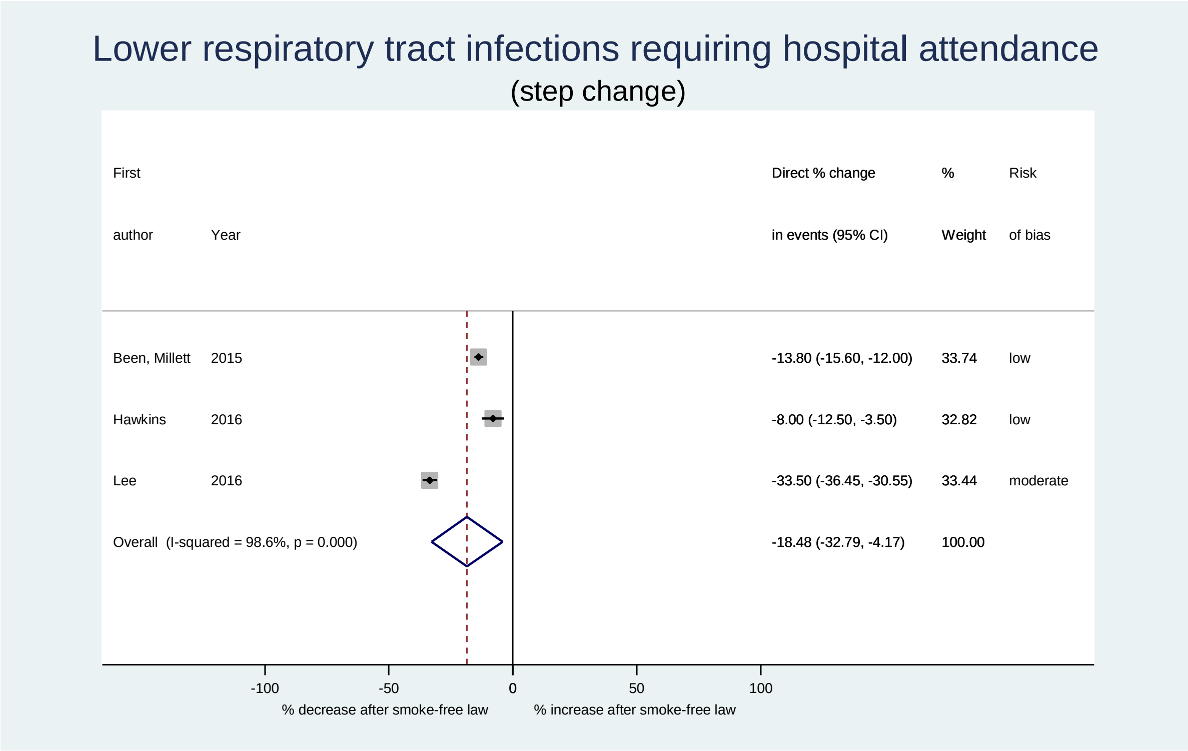

H – Lower respiratory tract infections requiring hospital attendance (gradual change)

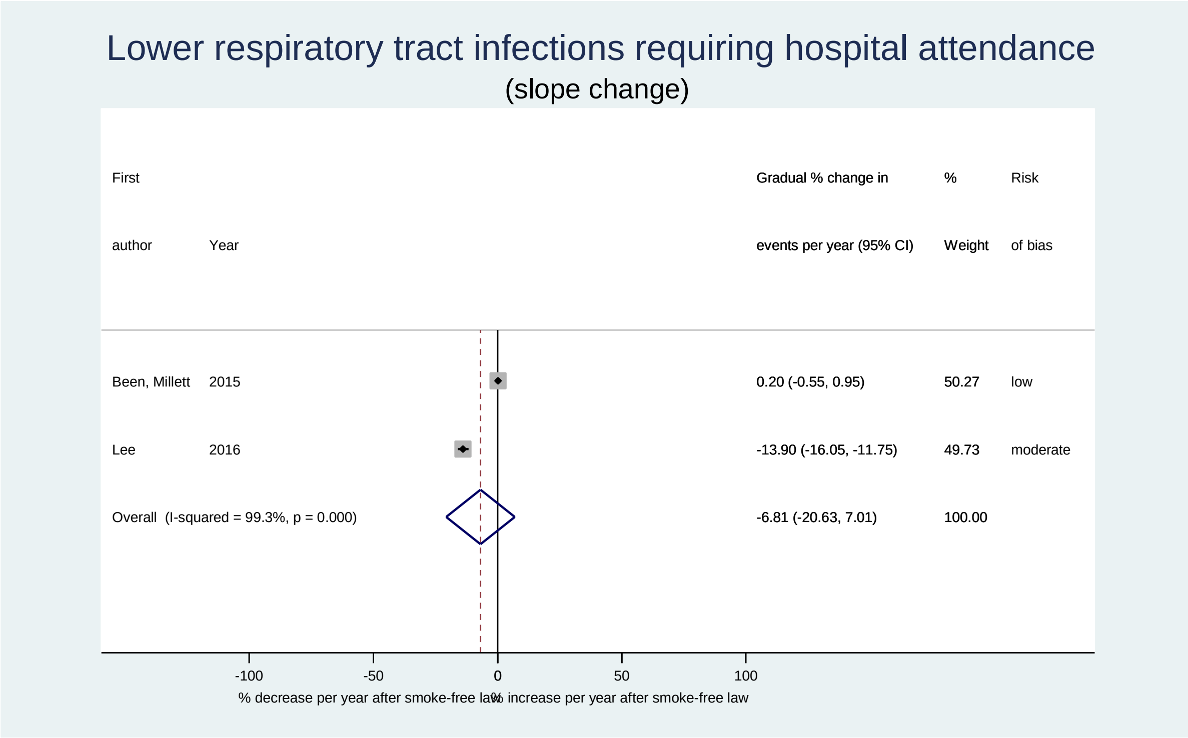

Figure S4: Subgroup analysis on comprehensiveness of smoke-free legislation

A – Preterm birth

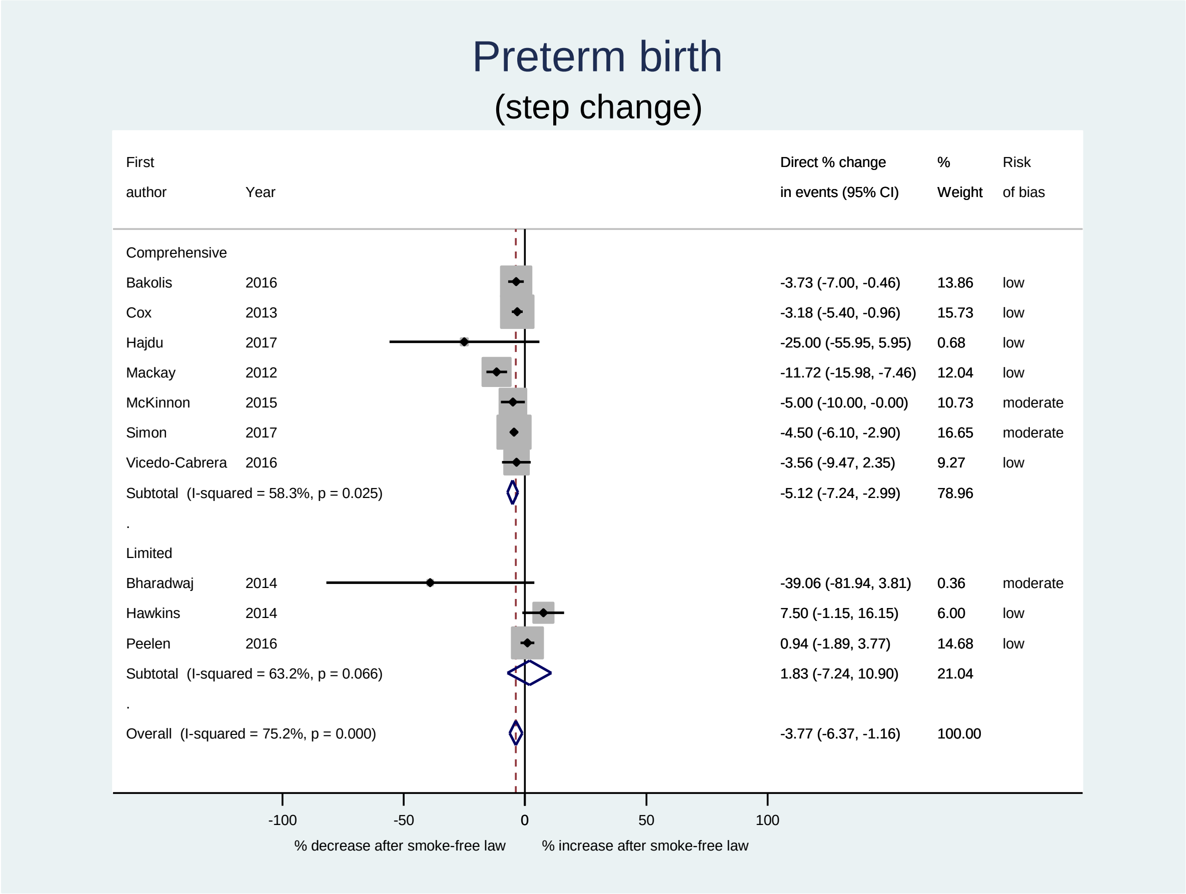

B – Asthma exacerbations requiring hospital attendance

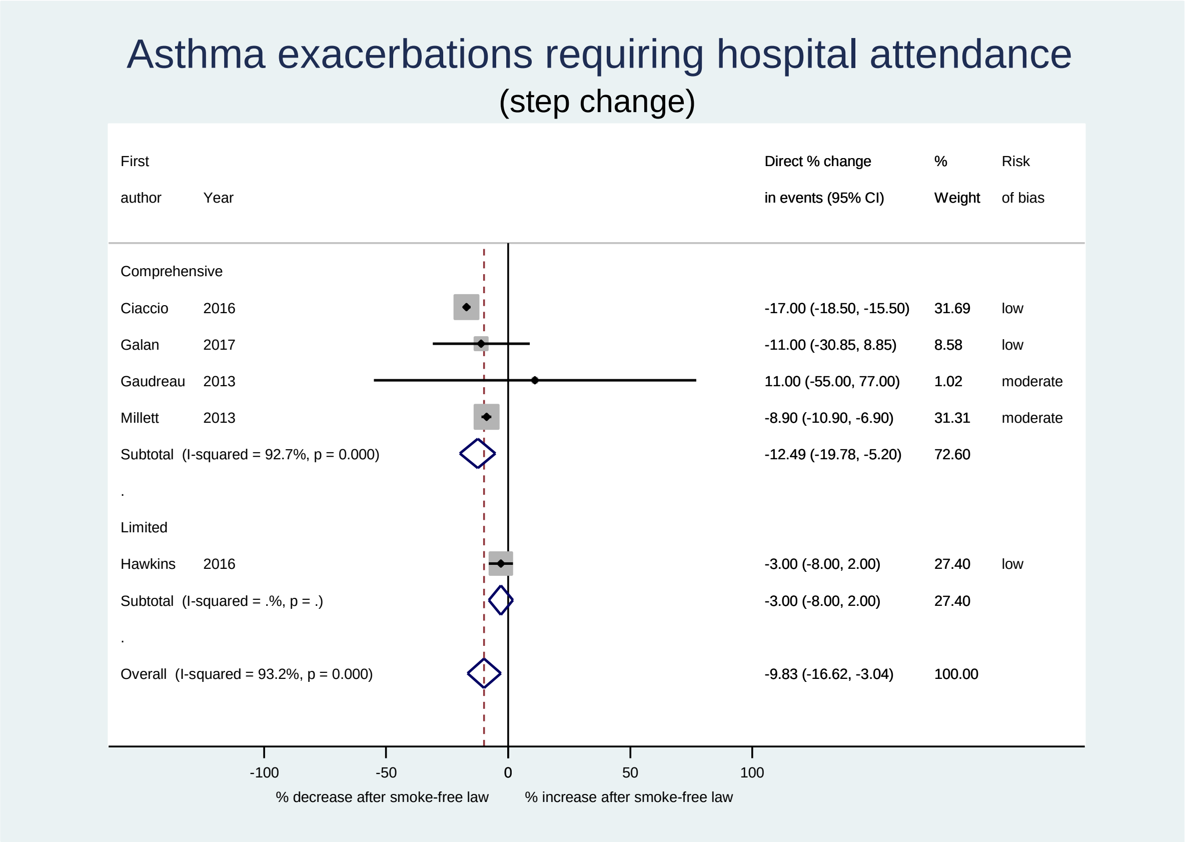

C – Birth weight

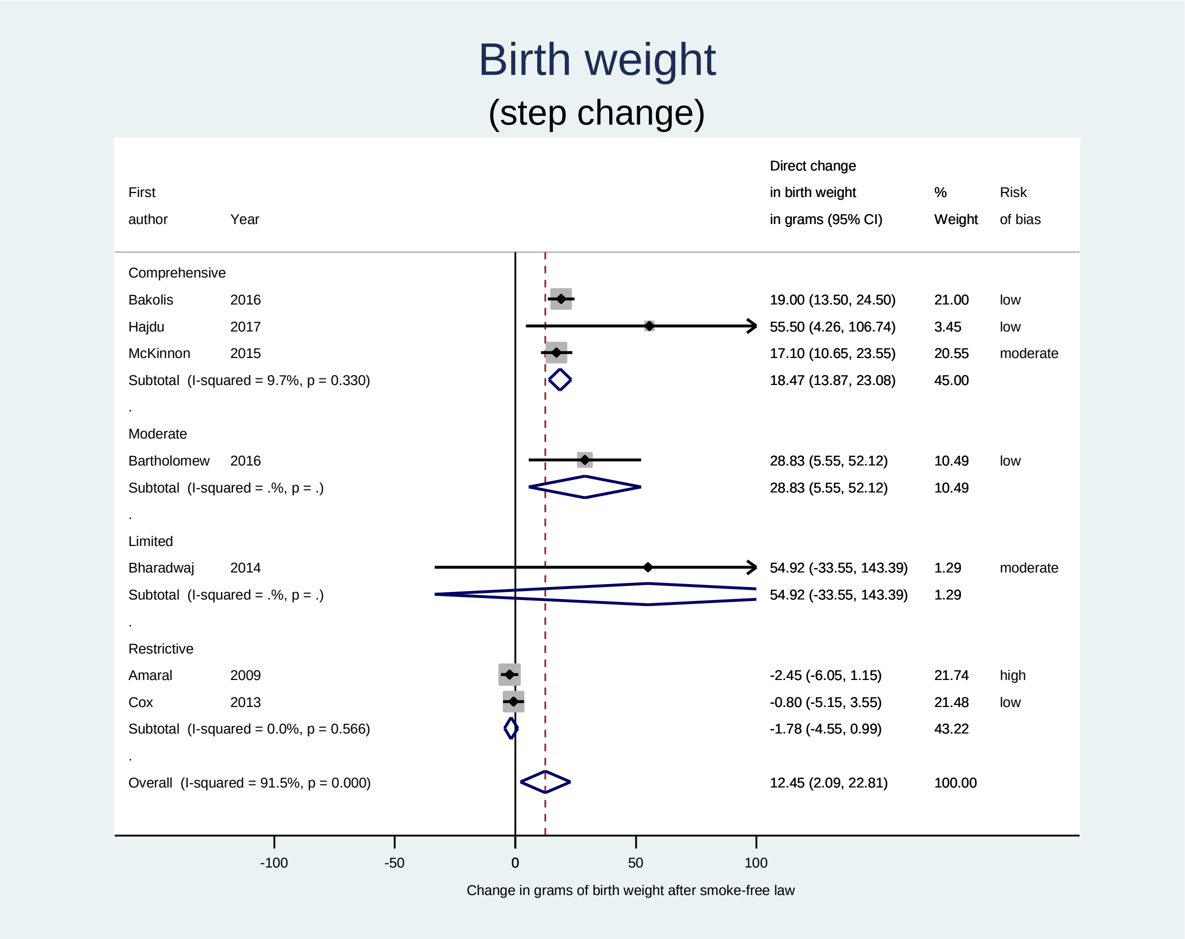

D – Low birth weight

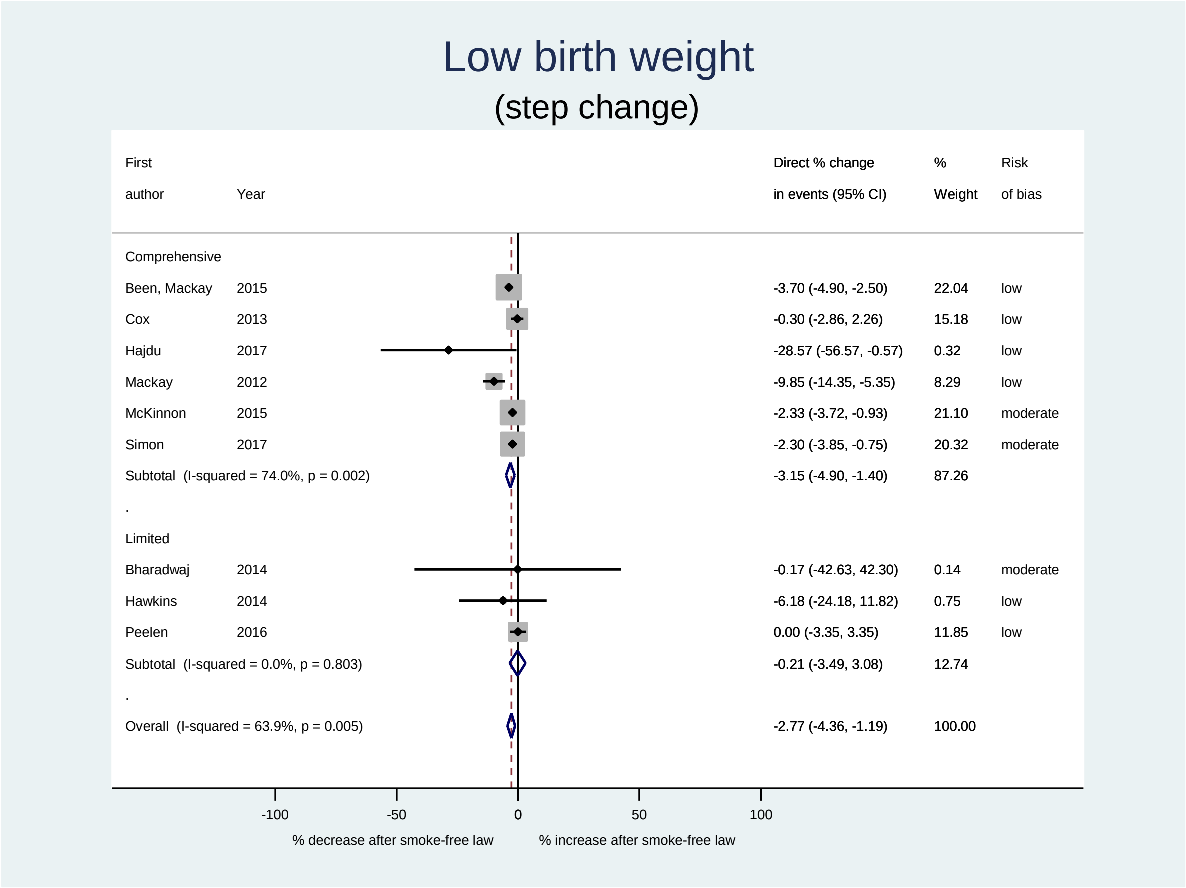

E – Very low birth weight

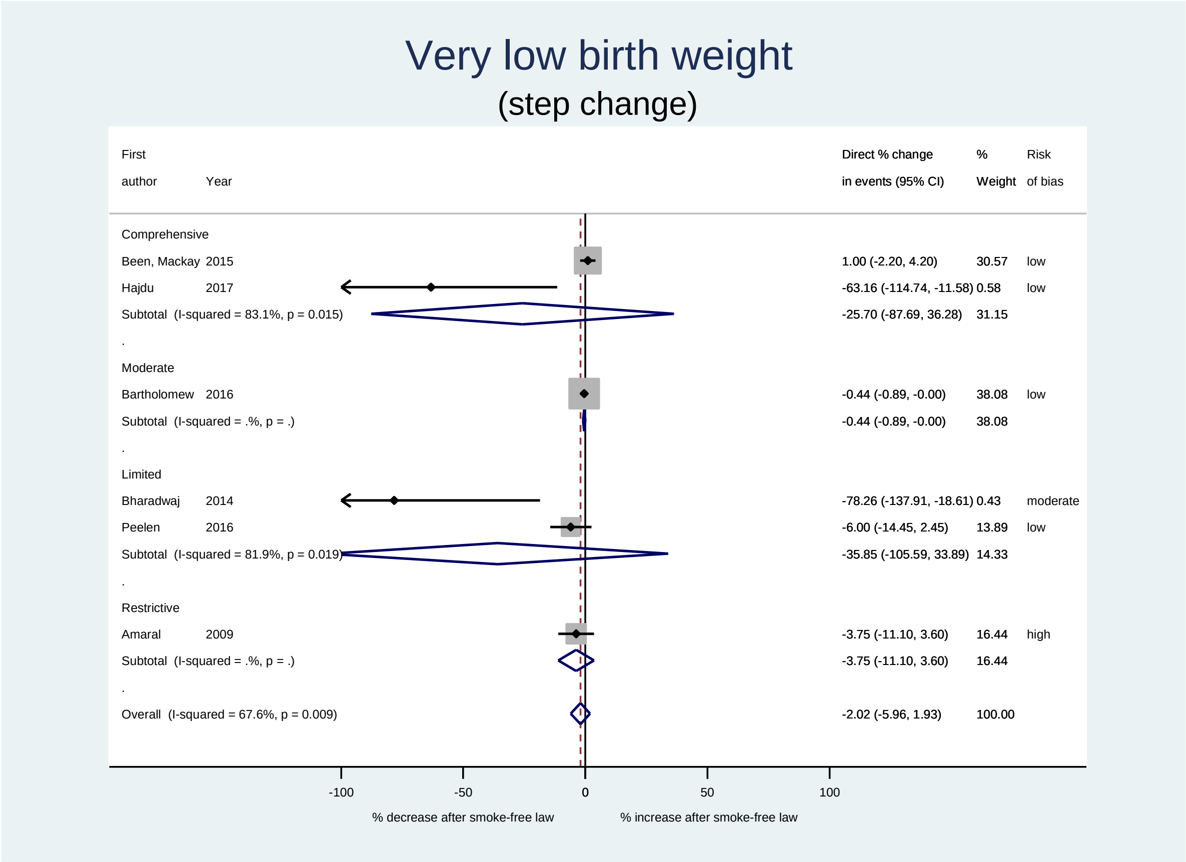

F – Small for gestational age

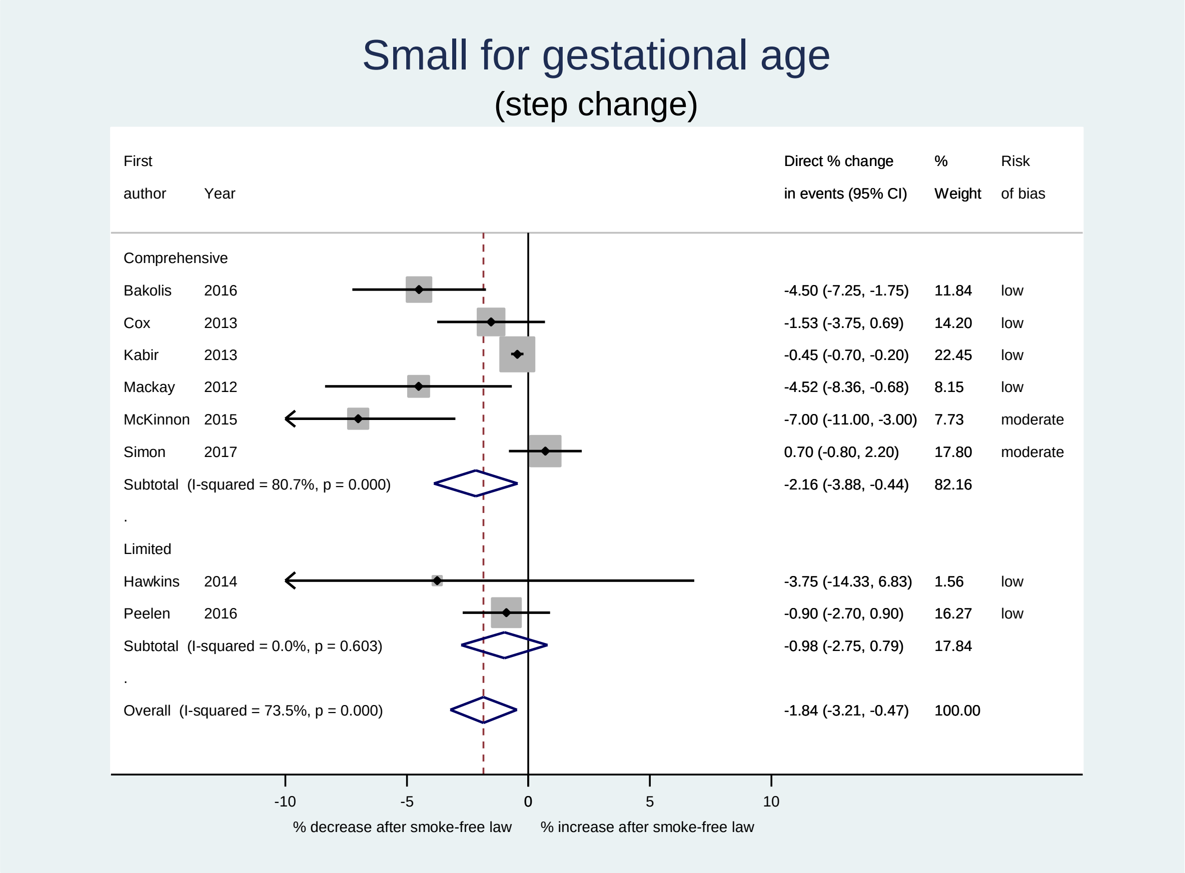

G – Very small for gestational age

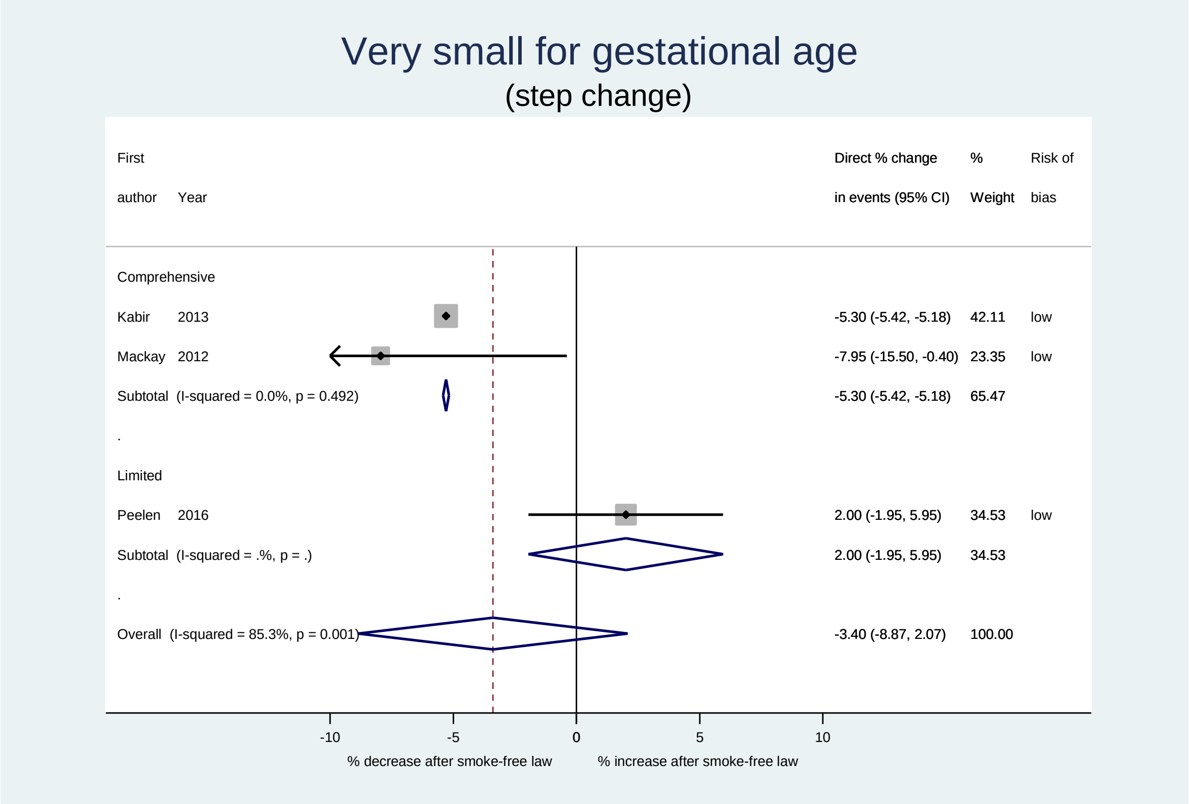

Table S6: Variations in association between MPOWER policies and outcomes according to socioeconomic status

Study (year)	SES Subgroup	Definition	Summary of findings
**Protect people from tobacco smoke**			
Amaral (2009)[2]	Maternal education	Education categorised as: high school dropout, high school graduate, some college, and college or more	Workplace smoking restrictions both state-wide and local were not associated with changes in BW, LBW, VLBW and GA overall, however associations varied according to maternal education. Local smoking ordinances: Only mothers with a college education or more showed a decrease in VLBW following the introduction of local smoking legislation (−0·13%* 95%CI −0·23 to −0·03). For BW and GA, no significant associations were found in any of the education categories. State-wide smoke-free legislation: A −9·25 grams decrease in BW (95%CI −14·87 to -3·63) was found in the sample of mothers who did not complete high school, and a −9·51 grams decrease in BW (95%CI −17·43 to −1·59) was found among mothers who completed college or more. In contrast, a 10·00 grams increase in BW (95%CI 2·83 to 17·17) was observed in the sample of mothers with a high school degree. Only mothers who did not complete high school showed a −0·04 week decrease in GA significant at p<0·1 (95%CI −0·08 to 0·00). Only mothers with a high school degree showed a significant −0·20%* decrease in VLBW (95%CI −0·34 to −0·06).
Bakolis (2016)[3]	Small area deprivation index	IMD quintiles (1: least deprived, 5: most deprived)	The introduction of smoke-free legislation was associated with an overall reduction in risk for LBW, VLBW, PTB, and SGA. Significant reductions observed across the four time windows (1, 2, 3, and 5 months) varied according to deprivation. Particularly the risk of VLBW among quintile 2 (ranging from OR 0·37 (95% CI: 0·15 to 0·88) to OR 0·65 (95% CI: 0·46 to 0·81)) and LBW among quintile 4 (ranging from OR 0·74 (95% CI: 0·59 to 0·92) to OR 0·88 (95% CI: 0·80 to 0·96)) was significantly reduced but not for the remaining birth outcomes or for quintiles 1, 3 and 5.
Been (2015)[6]	Small area deprivation index	IMD quintiles (1: least deprived, 5: most deprived)	Smoke-free legislation was associated with a significant immediate and gradual reduction in RTIs in children overall. The immediate change in RTIs did not vary significantly according to SES. The gradual change in RTIs varied significantly according to SES: the greatest decrease in RTI admissions was observed among the most deprived children: –1·5% (95% CI: –2·1 to –1·0) per year. The association between area-level deprivation index and RTI admission rates was clearly demonstrated, with >30% of RTIs occurring in the most deprived quintile.
Hajdu (2017)[17]	Maternal education	Education level categorised as: low education, high education.<br>High education: high school or university/college graduates	Changes in outcomes were usually more beneficial among parents with low education (maternal education: GA, PTB, VPTB, BW, LBW, VLBW; paternal education: GA, PTB, BW, LBW). Among parents with high education, there were no significant changes in the outcomes.
Mackay (2010)[24]	Small area deprivation index	Scottish Index of Multiple Deprivation quintiles (1: affluent, 5: deprived)	Smoke-free public places and workplaces were associated with a gradual −19·5% decrease per year (−22·4 to −16·5) in paediatric emergency asthma admissions. The gradual reduction in hospital admissions for asthma among children did not vary significantly according to SES.
McKinnon (2015)[27]	Maternal education	Education in years (12 or less, 13–15, and 16 or more)	State-wide smoke-free legislation was associated with reductions in the risk of in PTB, LBW, SGA, BW and VPTB. These changes did not vary significantly according to SES.
Millet (2013)[28]	Small area deprivation index	IMD quintiles (1: least deprived, 5: most deprived)	The introduction of smoke-free legislation was associated with both an immediate and gradual reduction in child asthma hospital admissions for all levels of deprivation. These reductions did not vary significantly according to SES.
Simón (2017)[33]	Parental socio-economic position based on mother's and/or father's occupational status	NR	No significant differences in outcomes between different levels of parental socio-economic position.
Vicedo-Cabrera (2016)[34]	Economical position of the canton†	SES scores derived from a PCA, in tertiles (lower, intermediate, and higher)	Smoke-free legislation was not associated with a significant change in PTB. The change in risk of PTB did not vary significantly according to SES.
**Raise taxes on tobacco**			
Bhai (2015)[36]	Household poverty level	Low SES: households that are below 100% of the US federal poverty level.<br>Middle SES: households that are	The largest reductions in asthma prevalence following increases in state cigarette excise tax were seen among low SES children: −3·2% per USD increase* (−4·8 to 1·6), as compared to middle SES (−1·3%* [−2·1 to −0·5], and high SES (−1·2%* [−1·8 to −0·6]).

		between 100% and 300% of the poverty level. High SES: households that are above 300% of the poverty level.	
Hawkins (2014)[19]	Maternal education	Education in years (12 or less, 13–15, and 16 or more)	Increased cigarette taxes were associated with a significant reduction in risk of several adverse birth outcomes. Mothers with the least amount of education had the strongest response to cigarette taxes with greater changes in BW, PTB, LBW, and SGA.

BW = Birth weight; GA = gestational age; IMD = Index of multiple deprivation; LBW = Low birth weight; PCA = Principal components analysis; PTB = Preterm birth; SES = socioeconomic status; SGA = Small for gestational age; VPTB = Very preterm birth.
*Percentage points
†The indicators related to the economical position of the canton were: foreign population, urban population, gross domestic product, educational level, population density, family size and status index.

Table S7: Association between implementation of tobacco control policies and secondary outcomes

MPOWER: Protect people from tobacco smoke (i.e. smoke-free legislation)							
**First author (year)**	**Details of intervention**	**Population at risk**	**Total number of events (n (%)) / overall mean (SD)**	**Slope before intervention (% change in events per year)**	**Direct change in events (step change; % (95% CI) / mean difference (95% CI))**	**Sustained change in events per year (slope change; % (95% CI))**	**Summary of findings**
**Stillbirth**							
Been, Mackay (2015)[5]	Public places and workplaces (including restaurants and bars)	9,984,278	52,163	NA due to non-linear time trend	−7·8% (−11·8 to −3·5)	NA	National smoke-free legislation was associated with an immediate 8% reduction in stillbirth.
Peelen (2016)[30]*	1st smoke-free law: Workplaces and public transport except : restaurants and bars† (allowing designated smoking areas)	1,983,761	9,163	NA due to non-linear time trend	−1·0% (−9·0 to 8·0)	NA	Both policies were not associated with significant changes in the odds of stillbirth.
	2nd smoke-free law: Expanding smoke-free law 1 to include restaurants and bars‡ (allowing designated smoking areas)				−3·0% (−12·0 to 6·0)		
**Gestational age**							
Adams (2013)[1]	Smoke-free restaurants	Group 1 – enrolled in Medicaid before pregnancy: 52,372	Group 1: 38·99 wk (95%CI not given)	NR	Group 1: 0·090 wk (−0·006 to 0·186)	NA	State-wide smoke-free restaurants were not associated with significant changes in GA.
		Group 2 – entered Medicaid during pregnancy: 104,211	Group 2: 39·21 wk (95%CI not given)		Group 2: 0·020 wk (−0·053 to 0·093)		
		Group 3 – enrolled in Medicaid either before or during pregnancy: 151,938	Group 3: 39·15 wk (95%CI not given)		Group 3: 0·031 wk (−0·030 to 0·092)		
Amaral (2009)[2]	Public places and workplaces (including restaurants)	NA	39·57 wk (39·02 to 40·12)	NR	State-wide: −0·0092 wk (−0·0369 to 0·0369)	NA	State-wide and local workplace smoke-free laws were not associated with significant changes in GA at birth.
					Local: 0·0049 wk (−0·0145 to 0·0244)		
Bakolis (2016)[3]	Public places and workplaces (including restaurants and bars)	NA	Median: 40 wk, P10−90: 37 to 41	NR	± 1 month: 0·01 wk (−0·02 to 0·02)<br>± 2 months: 0·02 wk (−0·02 to 0·05)<br>± 3 months: 0·02 wk (−0·02 to 0·04)<br>± 5 months: 0·02 wk (−0·01 to 0·04)	NA	National smoke-free legislation was not associated with significant changes in GA at birth
Bartholomew	1.Comprehensive (workplaces, restaurants, bars)	293,715	38·78 wk	NR	Comprehensive:	NA	County-wide comprehensive

(2016)[4]			(95%CI not given)		0·234 wk (0·101 to 0·368)		smoke-free legislation was associated with a 0·234 week (1·6 day) increase in GA at birth.
	2.Restrictive (workplaces, restaurants, no restriction in bars)				Restrictive: −0·007 wk (−0·089 to 0·102)		
	3.Moderate (workplaces, partial restriction in restaurants, no restriction in bars)				Moderate: −0·051 wk (−0·118 to 0·017)		
	4. Limited (partial restriction in workplaces, any restriction in restaurants, no restriction in bars).				Limited: 0·029 wk (−0·041 to 0·099)		
Briggs (2009)[9]	Workplaces:<br>1. some coverage	34,817,843	38·84 wk (33·69 to 43·99)	NR	State level −0·018 wk (−0·026 to −0·010)<br>County level 0·013 wk (0·007 to 0·019)	NA	State level 100% smoke-free workplace laws were associated with a 0·032 wk increase in GA, whereas 100% smoke-free bars were associated with a −0·035 wk decrease in GA. County level 100% smoke-free workplace laws were associated with a −0·027 wk decrease in GA, and smoke-free bar laws with a −0·160 wk decrease, whereas 100% smoke-free restaurant laws were associated with a 0·105 wk increase in GA.<br><br>State level qualified smoke-free workplace laws were associated with a 0·044 wk increase in GA, and smoke-free restaurants with a 0·079 wk increase. County level qualified smoke-free restaurant laws were associated with a 0·039 wk increase in GA.<br><br>State level workplace smoke-free laws with some coverage were associated with a −0·018 wk decrease in GA, restaurant smoke-free laws with some coverage with a −0·020 wk decrease, and smoke-free bar laws with some coverage with a −0·095 wk decrease. County level workplace smoke-free
	Workplaces:<br>2. qualified				State level 0·044 wk (0·015 to 0·073)<br>County level −0·049 wk (−0·098 to 0·000)		
	Workplaces:<br>3. 100% smoke-free				State level 0·032 wk (0·010 to 0·054)<br>County level −0·027 wk (−0·054 to 0·000)		
	Restaurants:<br>4. some coverage				State level −0·020 wk (−0·034 to −0·006)<br>County level −0·006 wk (−0·018 to 0·006)		
	Restaurants:<br>5. qualified				State level 0·079 wk (0·018 to 0·140)<br>County level 0·039 wk (0·019 to 0·059)		
	Restaurants:<br>6. 100% smoke-free				State level −0·011 wk (−0·027 to 0·005)<br>County level 0·105 wk (0·060 to 0·150)		
	Bars:<br>7. some coverage				State level −0·095 wk (−0·132 to −0·058)<br>County level −0·315 wk (−0·478 to −0·152)		

	Bars:<br>8. 100% smoke-free				State level −0·035 wk (−0·057 to −0·013)<br>County level −0·160 wk (−0·213 to −0·107)		laws with some coverage were associated with a 0·013 wk increase in GA, whereas smoke-free bar laws with some coverage were associated with a −0·315 wk decrease in GA.<br>.
Gao (2017)[14]	Any smoke-free law (including Restaurant/bar and workplace)	Maternal age 14−24: 13,918,429<br><br>Maternal age 25−34: 20,491,501<br><br>Maternal age 35−45: 5,744,190	Maternal age 14−24: Unknown<br><br>Maternal age 25−34: Unknown<br><br>Maternal age 35−45: Unknown	NR	Maternal age 14−24: 0·0033 (0·21)<br><br>Maternal age 25−34: 0·0107 (0·63)<br><br>Maternal age 35−45: 0·0119 (0·79)<br><br>Note: T-statistics in parenthesis	NA	Any smoke-free laws were not associated with any significant changes in PTB for any of maternal age groups.
	Smoke-free restaurants/bar law	Maternal age 14−24: 13,918,429<br><br>Maternal age 25−34: 20,491,501<br><br>Maternal age 35−45: 5,744,190	Maternal age 14−24: Unknown<br><br>Maternal age 25−34: Unknown<br><br>Maternal age 35−45: Unknown	NR	Maternal age 14−24: 0·0134 (0·82)<br><br>Maternal age 25−34: 0·0170 (0·96)<br><br>Maternal age 35−45: 0·0196 (1·35)<br><br>Note: T-statistics in parenthesis	NA	Restaurant/bar smoke-free laws were not associated with any significant changes in PTB for any of maternal age groups.
	Smoke-free workplace law	Maternal age 14−24: 13,918,429<br><br>Maternal age 25−34: 20,491,501<br><br>Maternal age 35−45: 5,744,190	Maternal age 14−24: Unknown<br><br>Maternal age 25−34: Unknown<br><br>Maternal age 35−45: Unknown	NR	Maternal age 14−24: −0·0094 (−0·64)<br><br>Maternal age 25−34: −0·0002 (−0·01)<br><br>Maternal age 35−45: 0·0027 (0·19)<br><br>Note: T-statistics in parenthesis	NA	Workplace smoke-free laws were not associated with any significant changes in PTB for any of maternal age groups.
Hajdu (2017)[17]	Public places and workplaces (including restaurants and bars)	18,755	NR	NR	0·189 wk (0·018 to 0·360)	NA	National smoke-free legislation was associated with a 0·2 week increase in GA.
Markowitz (2013)[26]	1. Workplaces: complete smoke-free law	Maternal age:<br>≤19 y: 54,132<br>20−24 y: 101,723<br>25−34 y: 183,763<br>≥35 y: 53,109	Maternal age:<br>≤19 y: 39·10 wk<br>20−24 y: 39·28<br>25−34 y: 39·26	NR	Maternal age:<br>≤19 y: −0·03 wk (−0·31 to 0·26)<br>NR for other maternal age groups	NA	Both state-wide complete smoke-free laws and smoking restrictions were associated with a 0·1 wk (0·8 day) increase in GA at birth among

	2. Workplaces: smoking restrictions (requiring designated smoking areas)		wk<br>≥35 y: 39·01<br><br>(95%CI not given)		Maternal age:<br>≤19 y: 0·01 wk (−0·28 to 0·31)<br>NR for other maternal age groups		women aged 25−34.
	3. Restaurants: complete smoke-free law				Maternal age:<br>≤19 y: 0·06 wk (−0·17 to 0·30)<br>20−24 y: 0·04 wk (−0·11 to 0·18)<br>25−34 y: 0·12 wk (0·05 to 0·19)<br>≥35 y: 0·07 wk (−0·08 to 0·21)		
	4. Restaurants: smoking restrictions (requiring designated smoking areas)				Maternal age:<br>≤19 y: 0·11 wk (−0·13 to 0·34)<br>20−24 y: −0·01 wk (−0·16 to 0·14)<br>25−34 y: 0·09 wk (0·04 to 0·15)<br>≥35 y: 0·07 wk (−0·05 to 0·20)		
**Very preterm birth**							
Hajdu (2017)[17]	Public places and workplaces (including restaurants and bars)	18,755	NR	NR	–0·9%§ (–1·9 to 0·1)	NA	National smoke-free legislation was not associated with significant changes in VPTB.
Mackay (2012)[25]	Public places and workplaces (including restaurants and bars)	709,756	6,265	NR	Crude: −16·60% (−25·92 to −6·11)	Crude: 2·40% (−3·37 to 8·52)	Smoke-free public places and workplaces were associated with an immediate 17% decrease in VPTB.
					Adjusted: −17·41% (−26·86 to −6·73)	Adjusted: 4·27% (−1·73 to 10·65)	
Markowitz (2013)[26]	1. Workplaces: complete smoke-free law	Maternal age:<br>≤19 y: 54,132<br>20−24 y: 101,723<br>25−34 y: 183,763<br>≥35 y: 53,109	Maternal age:<br>≤19 y: 2165 (4%)<br>20−24 y: 2034 (2%)<br>25−34 y: 3675 (2%)<br>≥35 y: 1062 (2%)<br><br>(95%CI not given)	NR	NR	NA	State-wide complete smoke-free laws were not associated with significant changes in VPTB, but state-wide restaurant smoking restrictions were associated with a 0·3 percentage point decrease in VPTB among women aged 25−34.
	2. Workplaces: smoking restrictions (requiring designated smoking areas)				NR		
	3. Restaurants: complete smoke-free law				Maternal age:<br>≤19 y: 0·2%§ (−1·4 to 1·8)<br>20−24 y: −0·1%§ (−0·3 to 0·1)<br>25−34 y: −0·1%§ (−0·3 to 0·1)<br>≥35 y: −0·2%§ (−0·6 to 0·2)		

	4. Restaurants: smoking restrictions (requiring designated smoking areas)				Maternal age:<br>≤19 y: −0·3%§ (−1·5 to 0·9)<br>20−24 y: −0·0%§ (−0·4 to 0·4)<br>25−34 y: −0·3%§ (−0·5 to −0·1)<br>≥35 y: −0·1%§ (−0·6 to 0·4)		
McKinnon (2015)[27]	Public places and workplaces (including restaurants and bars)	470,199	9,491	NR	Crude: −18% (−26 to −9)<br>Adjusted: −5% (−10 to −1)	NA	State-wide smoke-free legislation was associated with a 5% decrease in VPTB.
Peelen (2016)[30]*	1st smoke-free law: Workplaces and public transport except : restaurants and bars† (allowing designated smoking areas)	1,972,163	14,960	NA due to non-linear time trend	−6·0% (−14·0 to 3·0)	NA	National smoke-free workplaces and public transport were not associated with significant changes in the VPTB.<br>Expanding the smoke-free law to include restaurants and bars was associated with an 11% decrease in VPTB.
	2nd smoke-free law: Expanding smoke-free law 1 to include restaurants and bars‡ (allowing designated smoking areas)				−10·9% (−18·9 to −3·0)		
**Extremely preterm birth**							
Markowitz (2013)[26]	1. Workplaces: complete smoke-free law	Maternal age:<br>≤19 y: 54,132<br>20−24 y: 101,723<br>25−34 y: 183,763<br>≥35 y: 53,109	Maternal age:<br>≤19 y: 541 (1%)<br>20−24 y: 102 (1%)<br>25−34 y: NR (<1%)<br>≥35 y: 531 (1%)	NR	NR		State-wide complete smoke-free laws in restaurants were not associated with significant changes in EPTB. State-wide smoking restrictions in restaurants were associated with a 0·1 percentage point decrease in EPTB among women aged 25-34.
	2. Workplaces: smoking restrictions (requiring designated smoking areas)				NR		
	3. Restaurants: complete smoke-free law				Maternal age:<br>≤19 y: 0·1%§ (−0·7 to 0·9)<br>20−24 y: −0·0%§ (−0·1 to 0·1)<br>25−34 y: −0·0%§ (−0·1 to 0·0)<br>≥35 y: −0·1%§ (−0·3 to 0·1)		
	4. Restaurants: smoking restrictions (requiring designated smoking areas)				Maternal age:<br>≤19 y: −0·1%§ (−0·5 to 0·3)<br>20−24 y: −0·0%§ (−0·2 to 0·2)<br>25−34 y: −0·1%§ (−0·2 to −0·0)<br>≥35 y: −0·0%§ (−0·2 to 0·1)		
**Birth weight**							

Adams (2013)[1]	Smoke-free restaurants	Group 1 – enrolled in Medicaid before pregnancy: 57,283	Group 1: 3200 g (95%CI not given)	NR	Group 1: 14·76 g (−7·56 to 37·07)	NA	State-wide smoke-free restaurants were not associated with significant changes in BW.
		Group 2 – entered Medicaid during pregnancy: 113,464	Group 2: 3273 g (95%CI not given)		Group 2: 7·66 g (−12·10 to 27·43)		
		Group 3 – enrolled in Medicaid either before or during pregnancy: 165,686	Group 3: 3251 g (95%CI not given)		Group 3: 11·00 g (−5·51 to 27·50)		
Amaral (2009)[2]	Public places and workplaces (including restaurants)	NA	3375g (68)	NR	State-wide: −2·45 g (−6·05 to 1·15)	NA	State-wide and local workplace smoke-free laws were not associated with significant changes in BW.
					Local: −1·83 g (−5·16 to 1·49)		
Bakolis (2016)[3]	Public places and workplaces (including restaurants and bars)	NA	Median: 3380 g 10th, 90th centile: 2700 to 4030	NR	± 1 month: 17 (6 to 29)<br>± 2 months: 19 (10 to 27)<br>± 3 months: 20 (13 to 27)<br>± 5 months: 19 (14 to 25)	NA	National smoke-free legislation was associated with an increase between 17g and 19g in BW
Bartholomew (2016)[4]	1. Comprehensive (workplaces, restaurants, bars)	293,715	3302 g (95%CI not reported)	NR	Comprehensive: 28·83 g (5·55 to 52·12)	NA	County-wide comprehensive smoke-free legislation was associated with an immediate 28 g increase in BW, and moderate smoke-free legislation was associated with an immediate 23 g increase in BW.
	2. Restrictive (workplaces, restaurants, no restriction in bars)				Restrictive: −2·77 g (−16·80 to 11·25)		
	3. Moderate (workplaces, partial restriction in restaurants, no restriction in bars)				Moderate: −23·34 g (−34·16 to −12·52)		
	4. Limited (partial restriction in workplaces , any restriction in restaurants, no restriction in bars).				Limited: −5·58 g (−16·24 to 5·09)		
Bharadwaj (2014)[8]	Restaurants and bars (in addition to already existing smoke-free laws in public places and workplaces)	NA	Treatment group before smoke-free legislation: 3444 (2039 to 4849)	NR	54·92 g (−33·55 to 143·39)	NA	Smoke-free restaurants and bars were not associated with significant changes in BW among women working in restaurants and bars.
Briggs (2009)[9]	Workplaces:<br>1. some coverage	34,817,843	3311 g (2119 to 4504)	NR	State level −1·105g (−2·597 to 0·387)<br>County level −0·039 (−1·058 to 0·980)	NA	State level 100% smoke-free workplace laws were associated with a −13 g decrease in BW, whereas

	Workplaces:<br>2. qualified				State level −9·375 g (−15·010 to −3·740)<br>County level 3·209 g (−6·150 to 12·568)		100% smoke-free restaurant laws were associated with a 6 g increase in BW.<br><br>State level qualified smoke-free workplace laws were associated with a −9 g decrease in BW, whereas qualified smoke-free restaurant laws were associated with a 35 g increase in BW. County level qualified smoke-free restaurant laws were associated with a 8 g increase in BW.<br><br>State level smoke-free restaurant laws with some coverage were associated with a −23 g decrease in BW. County level smoke-free restaurant laws with some coverage were associated with a 5 g increase in BW.
	Workplaces:<br>3. 100% smoke-free				State level −13·093 g (−17·123 to −9·063)<br>County level 3·058 g (−2·003 to 8·119)		
	Restaurants:<br>4. some coverage				State level −23·027 g (−25·501 to −20·554)<br>County level 5·076 g (3·006 to 7·146)		
	Restaurants:<br>5. qualified				State level 35·192 g (23·883 to 46·501)<br>County level 7·759 g (3·996 to 11·522)		
	Restaurants:<br>6. 100% smoke-free				State level 5·613 g (2·648 to 8·578)<br>County level −8·106 g (−16·795 to 0·583)		
	Bars:<br>7. some coverage				State level 3·275 g (−3·712 to 10·262)<br>County level 3·922 g (−26·650 to 34·494)		
	Bars:<br>8. 100% smoke-free				State level −1·822 g (−5·752 to 2·108)<br>County level −4·744 g (−14·891 to 5·403)		
Cox (2013)[11]	Public places and workplaces (excluding catering industry)	606,877	3347g (519)	3·3g (1·8 to 4·8)	−0·8 (−5·2 to 3·5)	−1·1 (−2·8 to 0·6)	Smoke-free public places and workplaces were not associated with significant changes in BW.
	Restaurants<br>(in addition to already existing smoke-free laws in public places and workplaces)	606,877	3347g (519)	2·4 (1·3 to 3·5)	3·0 (−1·3 to 7·2)	−0·8 (−2·3 to 0·6)	Expanding smoke-free legislation to include restaurants was not associated with significant changes in BW.
	Bars serving food (in addition to already existing smoke-free laws in public places and workplaces, including restaurants)	606,877	3347g (519)	3·1 (2·6 to 3·6)	−3·4 (−8·9 to 2·2)	−3·1 (−7·3 to 1·1)	Expanding smoke-free legislation to include bars was not associated with significant changes in BW.
Gao (2017)[14]	Any smoke-free law (including Restaurant/bar and workplace)	Maternal age 14−24: 13,918,429<br><br>Maternal age 25−34: 20,491,501	Maternal age 14−24: Unknown<br><br>Maternal age 25−34:	NR	Maternal age 14−24: −0·8954 (−0·57)<br><br>Maternal age 25−34: 0·497 (0·29)	NA	Any smoke-free laws were not associated with any significant changes in BW for any of maternal age groups.

		Maternal age 35−45: 5,744,190	Unknown<br><br>Maternal age 35−45: Unknown		Maternal age 35−45: 0·9906 (0·57)<br><br>Note: T-statistics in parenthesis		
	Restaurants/bar smoke-free law	Maternal age 14−24: 13,918,429<br><br>Maternal age 25−34: 20,491,501<br><br>Maternal age 35−45: 5,744,190	Maternal age 14−24: Unknown<br><br>Maternal age 25−34: Unknown<br><br>Maternal age 35−45: Unknown	NR	Maternal age 14−24: 1·6462 (0·65)<br><br>Maternal age 25−34: 1·8550 (0·84)<br><br>Maternal age 35−45: 2·5041 (1·20)<br><br>Note: T-statistics in parenthesis	NA	Restaurant/bar smoke-free laws were not associated with any significant changes in BW for any of maternal age groups.
	Workplace smoke-free law	Maternal age 14−24: 13,918,429<br><br>Maternal age 25−34: 20,491,501<br><br>Maternal age 35−45: 5,744,190	Maternal age 14−24: Unknown<br><br>Maternal age 25−34: Unknown<br><br>Maternal age 35−45: Unknown	NR	Maternal age 14−24: −1·9251 (−1·27)<br><br>Maternal age 25−34: 0·4556 (0·26)<br><br>Maternal age 35−45: 1·2995 (0·70)<br><br>Note: T-statistics in parenthesis	NA	Workplace smoke-free laws were not associated with any significant changes in BW for any of maternal age groups.
Hajdu (2017)[17]	Public places and workplaces (including restaurants and bars)	18,753	NR	NR	55·5 g (4·3 to 106·7)	NA	National smoke-free legislation was associated with a 56 g increase in BW.
Hawkins (2014)[19]	100% smoke-free workplaces and restaurants	NA	3339 g (95%CI not given)	NR	−0·03g (−3·51 to 3·46)	NA	State-wide smoke-free workplaces and restaurants were not associated with significant changes in BW.
Markowitz (2013)[26]	1. Workplaces: complete smoke-free law	Maternal age:<br>≤19 y: 54,132<br>20−24 y: 101,723<br>25−34 y: 183,763<br>≥35 y: 53,109	Maternal age:<br>≤19 y: 3182 g<br>20−24 y: 3275 g<br>25−34 y: 3372 g<br>≥35 y: 3368 g<br><br>(95%CI not given)	NR	1. Maternal age:<br>≤19 y: 11·22 g (−29·51 to 51·96)<br>NR for other maternal age groups	NA	State-wide complete smoking smoke-free laws or smoking restrictions in restaurants were not associated with significant changes in BW.
	2. Workplaces: smoking restrictions (requiring designated smoking areas)				2. Maternal age:<br>≤19 y: 0·24 g (−47·39 to 47·87)<br>NR for other maternal age groups		
	3. Restaurants: complete smoke-free law				3. Maternal age:<br>≤19 y: 15·17 g (−29·88 to 60·22)		

					20−24 y: 4·00 g<br>(−42·13 to 50·13)<br>25−34 y: 16·10 g<br>(−3·26 to 35·46)<br>≥35 y: −23·84 g<br>(−57·70 to 10·02)		
	4. Restaurants: smoking restrictions (requiring designated smoking areas)				4. Maternal age:<br>≤19 y: 5·72 g<br>(−35·83 to 47·28)<br>20−24 y: 15·51 g<br>(−23·98 to 55·00)<br>25−34 y: 7·27 g<br>(−10·55 to 25·09)<br>≥35 y: −17·97 g<br>(−49·42 to 13·78)		
McKinnon (2015)[27]	Public places and workplaces (including restaurants and bars)	470,136	3387g (3386 to 3389)	NR	Crude: 18·6g (12·3 to 24·9)<br>Adjusted: 17·1g (10·7 to 23·6)	NA	State-wide smoke-free legislation was associated with a 17g increase in BW.
**Low birth weight**							
Amaral (2009)[2]	Public places and workplaces (including restaurants)	NR	NR	NR	State−wide: −0·00%§ (−0·14 to 0·14)<br>Local: 0·09%§ (−0·07 to 0·25)	NA	State-wide and local workplace smoke-free laws were not associated with significant changes in LBW.
Bakolis (2016)[3]	Public places and workplaces (including restaurants and bars)	1,800,906	102,006 (6%)	NR	± 1 month: −13·3% (−22·0 to −4·7)<br>± 2 months: −12·4% (−18·1 to −5·7)<br>± 3 months: −11·4% (−16·2 to −6·6)<br>± 5 months: −7·6% (−11·4 to −3·8)	NA	National smoke-free legislation was associated with a reduction between 8% and 14% in LBW.
Bartholomew (2016)[4]	1. Comprehensive (workplaces, restaurants, bars)	293,715	20,002	NR	Comprehensive: −0·005%§ (−0·013 to 0·004)	NA	County-wide smoke-free legislation was not associated with significant changes in LBW.
	2. Restrictive (workplaces, restaurants, no restriction in bars)				Restrictive: 0·002%§ (−0·005 to 0·008)		
	3. Moderate (workplaces, partial restriction in restaurants, no restriction in bars)				Moderate: 0·008%§ (−0·001 to 0·016)		
	4. Limited (partial restriction in workplaces , any restriction in restaurants, no restriction in bars).				Limited: −0·001%§ (−0·006 to 0·004)		

Been, Mackay (2015)[5]	Public places and workplaces (including restaurants and bars)	9,933,349	606,800	NA due to non-linear time trend	−3·7% (−4·8 to −2·4)	NA	National smoke-free legislation was associated with an immediate 4% reduction in LBW.
Bharadwaj (2014)[8]	Restaurants and bars (in addition to already existing smoke-free laws in public places and workplaces)	822 (I); 3185 (C)	49 (I); 185 (C)	NR	−0·01%§ (−2·56 to 2·54)	NA	Smoke-free restaurants and bars were not associated with significant changes in LBW among women working in restaurants and bars.
Briggs (2009)[9]	Workplaces:<br>1. some coverage	34,817,843	2,785,427 (8%)	NR	State level −0·001%§ (−0·002 to −0·000)<br>County level 0·000%§ (−0·000 to 0·000)	NA	State level smoke-free workplace laws with some coverage were associated with a −0·001 percentage point decrease in LBW, whereas smoke-free restaurant laws with some coverage were associated with a 0·003 percentage point increase in LBW.<br><br>County level smoke-free restaurant laws with some coverage were associated with a −0·002 percentage point decrease in LBW.
	Workplaces:<br>2. qualified				State level 0·001%§ (−0·002 to 0·004)<br>County level −0·002%§ (−0·007 to 0·003)		
	Workplaces:<br>3. 100% smoke-free				State level 0·000%§ (−0·002 to 0·002)<br>County level −0·002%§ (−0·004 to 0·000)		
	Restaurants:<br>4. some coverage				State level 0·003%§ (0·002 to 0·004)<br>County level −0·002%§ (−0·003 to −0·001)		
	Restaurants:<br>5. qualified				State level 0·003%§ (−0·002 to 0·008)<br>County level 0·001%§ (−0·001 to 0·003)		
	Restaurants:<br>6. 100% smoke-free				State level 0·000%§ (−0·001 to 0·001)<br>County level 0·002%§ (−0·002 to 0·006)		
	Bars:<br>7. some coverage				State level −0·002%§ (−0·005 to 0·001)<br>County level −0·002%§ (−0·016 to 0·012)		

	Bars:<br>8. 100% smoke-free				State level 0·000%§ (−0·002 to 0·002)<br>County level −0·001%§ (−0·005 to 0·003)		
Cox (2013)[11]	Public places and workplaces (excluding catering industry)	606,877	28,678	NR	Single smoke-free law¶: −0·19% (−2·48 to 2·16)<br>Final model‖: no significant changes	Single smoke-free law¶: 0·39% (−1·38 to 2·20)<br>Final model‖: no significant changes	Smoke-free public places and workplaces were not associated with significant changes in LBW.
	Restaurants<br>(in addition to already existing smoke-free laws in public places and workplaces)	606,877	28,678	NR	Single smoke-free law¶: 0·06 (−2·33 to 2·52)<br>Final model‖: no significant changes	Single smoke-free law¶: 0·21% (−1·44 to 1·89)<br>Final model‖: no significant changes	Expanding smoke-free legislation to include restaurants was not associated with significant changes in LBW.
	Bars serving food (in addition to already existing smoke-free laws in public places and workplaces, including restaurants)	606,877	28,678	NR	Single smoke-free law¶: −0·49% (−2·54 to 1·60)<br>Final model‖: no significant changes	Single smoke-free law¶: −1·26% (−4·35 to 1·92)<br>Final model‖: no significant changes	Expanding smoke-free legislation to include bars was not associated with significant changes in LBW.
Gao (2016)[14]	Any smoke-free law (including Restaurant/bar and workplace)	Maternal age 14−24: 13,918,429<br>Maternal age 25−34: 20,491,501<br>Maternal age 35−45: 5,744,190	Maternal age 14−24: Unknown<br>Maternal age 25−34: Unknown<br>Maternal age 35−45: Unknown	NR	Maternal age 14−24: 0·0010 (2·59)<br>Maternal age 25−34: 0·0003 (0·75)<br>Maternal age 35−45: −0·0002 (−0·27)<br>Note: T-statistics in parenthesis	NA	Smoke-free laws were associated with a 1·3% increase for LBW for younger mothers (age 14 to 24), however not for the older age groups.
	Restaurants/bar smoke-free law	Maternal age 14−24: 13,918,429<br>Maternal age 25−34:	Maternal age 14−24: Unknown<br>Maternal age	NR	Maternal age 14−24: 0·0002 (0·51)<br>Maternal age 25−34: 0·0002 (0·52)	NA	Restaurant/bar smoke-free laws were not associated with any significant changes in LBW for any of maternal age groups.

		20,491,501<br><br>Maternal age 35−45: 5,744,190	25−34: Unknown<br><br>Maternal age 35−45: Unknown		Maternal age 35−45: −0·0006 (−0·87)<br><br>Note: T-statistics in parenthesis		
	Workplace smoke-free law	Maternal age 14−24: 13,918,429<br><br>Maternal age 25−34: 20,491,501<br><br>Maternal age 35−45: 5,744,190	Maternal age 14−24: Unknown<br><br>Maternal age 25−34: Unknown<br><br>Maternal age 35−45: Unknown	NR	Maternal age 14−24: 0·0010 (1·84)<br><br>Maternal age 25−34: 0·0005 (1·10)<br><br>Maternal age 35−45: −0·0004 (−0·62)<br><br>Note: T-statistics in parenthesis	NA	Workplace smoke-free laws were not associated with any significant changes in LBW for any of maternal age groups.
Hade (2011)[16]	Public places and workplaces (including restaurants and bars)	583,530	50,185	NR	ARIMA: 1·0% (−2·0 to 4·0)	ARIMA: −1·4% (−1·5 to −1·3)	Smoke-free public places and workplaces were associated with a gradual 1% per year decrease in LBW.
					Logistic regression: 2·0% (−1·0 to 6·0)	Logistic regression: NA	
Hajdu (2017)[17]	Public places and workplaces (including restaurants and bars)	18,753	NR	NR	–2·2%§ (–4·4 to –0·0)	NA	National smoke-free legislation was associated with a 2 percentage point decrease in LBW.
Hankins (2016)[18]	1. Workplaces	NR	NR	NR	Workplaces: 0·05%§ (−0·05 to 0·15)	NA	State-wide or county smoke-free workplaces, restaurants, or bars were not associated with significant changes in LBW.
	2. Restaurants				Restaurants: −0·11%§ (−0·27 to 0·05)		
	3. Bars				Bars: 0·09%§ (−0·03 to 0·21)		
Hawkins (2014)[19]	100% smoke-free workplaces and restaurants	16,198,654	890,926	NR	−0·34%§ (−1·33 to 0·65)	NA	State-wide smoke-free workplaces and restaurants were not associated with significant changes in LBW.
Mackay (2012)[25]	Public places and workplaces (including restaurants and bars)	709,279	39,623	NR	Crude: −9·53% (−13·82 to −5·04)	Crude: −1·08% (−3·42 to 1·32)	Smoke-free public places and workplaces were associated with an immediate 10% decrease in LBW.

					Adjusted: −9·85% (−14·24 to −5·23)	Adjusted: 0·89% (−1·56 to 3·41)	
Markowitz (2013)[26]	1. Workplaces: complete smoke-free law	Maternal age:<br>≤19 y: 54,132<br>20−24 y: 101,723<br>25−34 y: 183,763<br>≥35 y: 53,109	Maternal age:<br>≤19 y: 4872 (9%)<br>20−24 y: 7121 (7%)<br>25−34 y: 9188 (5%)<br>≥35 y: 3718 (7%)	NR	1. NR	NA	State-wide complete smoke-free laws or smoking restrictions in restaurants were not associated with significant changes in LBW.
	2. Workplaces: smoking restrictions (requiring designated smoking areas)				2. NR		
	3. Restaurants: complete smoke-free law				3. Maternal age:<br>≤19 y: 0·8%§ (−0·5 to 2·1)<br>20−24 y: −0·3%§ (−0·7 to 0·1)<br>25−34 y: −0·6%§ (−1·2 to 0·0)<br>≥35 y: −0·5%§ (−1·1 to 0·1)		
	4. Restaurants: smoking restrictions (requiring designated smoking areas)				4. Maternal age:<br>≤19 y: −0·5%§ (−1·8 to 0·8)<br>20−24 y: −0·4%§ (−0·8 to 0·0)<br>25−34 y: −0·4%§ (−1·0 to 0·2)<br>≥35 y: 0·1%§ (−1·1 to 1·3)		
McKinnon (2015)[27]	Public places and workplaces (including restaurants and bars)	470,136	19,982	NR	Crude: −0·12%§ (−0·18 to −0·06)	NA	State-wide smoke-free legislation was associated with a 0·1 percentage point decrease in LBW.
					Adjusted: −0·10%§ (−0·16 to −0·04)		
Page (2012)[29]	Public places and workplaces (including restaurants and bars)	6,717 (I); 32,293 (C)	558 (I); 2,612 (C)	NR	Crude: −13·3% (−28·4 to 5·0)	NA	City-wide smoke-free public places and workplaces were not associated with significant changes in LBW.
					Adjusted: 4·4% (−17·6 to 32·3)		
Peelen (2016)[30]*	1st smoke-free law: Workplaces and public transport except : restaurants and bars† (allowing designated smoking areas)	1,972,163	95,144	NA due to non-linear time trend	0·0% (−2·9 to 3·8)	NA	Both policies were not associated with significant changes in the odds of LBW.
	2nd smoke-free law: Expanding smoke-free law 1 to include restaurants and bars‡ (allowing designated smoking areas)				−2·9% (−5·7 to 1·0)		
Simón (2017)[33]	1st smoke-free law: Complete smoke-free workplaces and partial smoke-free restaurants and bars	5,293,700	489,443 (9·2%)	NR	1st smoke-free law: 0·6% (–1·0 to 2·1)	NA	National partial smoke-free legislation was not associated with changes in LBW. The subsequent national
	2nd smoke-free law: Public places and workplaces (including restaurants and bars)				2nd smoke-free law: –2·3% (–3·8 to –0·7)		

							comprehensive smoke-free legislation was associated with a 2% decrease in LBW.
**Very low birth weight**							
Amaral (2009)[2]	Public places and workplaces (including restaurants)	NR	NR	NR	State-wide: −0·03%§ (−0·09 to 0·03)<br>Local: −0·03%§ (−0·09 to 0·03)	NA	Both state-wide and local workplace smoke-free laws were not associated with significant changes in VLBW.
Bakolis (2016)[3]	Public places and workplaces (including restaurants and bars)	1,800,906	14,517 (1%)	NR	± 1 month: −27·8% (−45·8 to −3·0)<br>± 2 months: −28·8% (−40·8 to −13·9)<br>± 3 months: −31·8% (−40·8 to −20·9)<br>± 5 months: −27·8% (−35·8 to −18·9)	NA	National smoke-free legislation was associated with a 28% to 32% reduction in VLBW.
Bartholomew (2016)[4]	1.Comprehensive (workplaces, restaurants, bars)<br>2.Restrictive (workplaces, restaurants, no restriction in bars)<br>3.Moderate (workplaces, partial restriction in restaurants, no restriction in bars)<br>4.Limited (partial restriction in workplaces , any restriction in restaurants, no restriction in bars).	293,715	2,643	NR	Comprehensive: −0·004%§ (−0·008 to −0·000)<br>Restrictive: −0·002%§ (−0·004 to −0·000)<br>Moderate: 0·001%§ (−0·001 to 0·004)<br>Limited: −0·001%§ (−0·003 to 0·001)	NA	County-wide comprehensive smoke-free legislation was associated with an immediate 0·004 percentage point decrease in VLBW, and restrictive smoke-free legislation was associated with an immediate 0·002 percentage point decrease in VLBW.
Been, Mackay (2015)[5]	Public places and workplaces (including restaurants and bars)	9,933,349	97,246	NA due to non-linear time trend	1·0% (−2·2 to 4·2)	NA	No evidence of an association between national smoke-free legislation and VLBW.
Bharadwaj (2014)[8]	Restaurants and bars (in addition to already existing smoke-free laws in public places and workplaces)	822 (I); 3185 (C)	14 (I); 43 (C)	NR	−1·8%§ (−3·2 to −0·4)	NA	Smoke-free restaurants and bars were associated with an immediate 2 percentage points decrease in VLBW among women working in restaurants and bars.
Gao (2017)[14]	Any smoke-free law (including Restaurant/bar and workplace)	Maternal age 14−24: 13,918,429<br>Maternal age 25−34: 20,491,501	Maternal age 14−24: Unknown<br>Maternal age 25−34: Unknown	NR	Maternal age 14−24: 0·0004 (2·14)<br>Maternal age 25−34: −0·0000 (−0·24)<br>Maternal age 35−45:	NA	Any smoke-free laws were associated with a 3% increase in VLBW for younger mothers (age 14 to 24), however not for the older age groups.

		Maternal age 35−45: 5,744,190	Maternal age 35−45: Unknown		−0·0000 (−0·08)<br><br>Note: T-statistics in parenthesis		
	Restaurants/bar smoke-free law	Maternal age 14−24: 13,918,429<br><br>Maternal age 25−34: 20,491,501<br><br>Maternal age 35−45: 5,744,190	Maternal age 14−24: Unknown<br><br>Maternal age 25−34: Unknown<br><br>Maternal age 35−45: Unknown	NR	Maternal age 14−24: 0·0004 (2·54)<br><br>Maternal age 25−34: 0·0000 (0·02)<br><br>Maternal age 35−45: −0·0002 (−0·77)<br><br>Note: T-statistics in parenthesis	NA	Restaurant/bar smoke-free laws were associated with a 3% increase in VLBW for younger mothers (age 14 to 24), however not for the older age groups.
	Workplace smoke-free law	Maternal age 14−24: 13,918,429<br><br>Maternal age 25−34: 20,491,501<br><br>Maternal age 35−45: 5,744,190	Maternal age 14−24: Unknown<br><br>Maternal age 25−34: Unknown<br><br>Maternal age 35−45: Unknown	NR	Maternal age 14−24: 0·0004 (2·08)<br><br>Maternal age 25−34: 0·0000 (0·35)<br><br>Maternal age 35−45: −0·0002 (−0·92)<br><br>Note: T-statistics in parenthesis	NA	Workplace smoke-free laws were associated with an increase in VLBW for younger mothers (age 14 to 24), however not for the older age groups.
Hajdu (2017)[17]	Public places and workplaces (including restaurants and bars)	18,753	NR	NR	–1·2%§ (–2·2 to –0·2)	NA	National smoke-free legislation was associated with a 1 percentage point decrease in VLBW.
Markowitz (2013)[26]	1. Workplaces: complete smoke-free law	Maternal age:<br>≤19 y: 54,132<br>20−24 y: 101,723<br>25−34 y: 183,763<br>≥35 y: 53,109	Maternal age:<br>≤19 y: 541 (1%)<br>20−24 y: 1,017 (1%)<br>25−34 y: 1,838 (1%)<br>≥35 y: 531 (1%)	NR	1. NR	NA	State-wide complete smoke-free laws or smoking restrictions in restaurants were not associated with significant changes in VLBW.
	2. Workplaces: smoking restrictions (requiring designated smoking areas)				2. NR		
	3. Restaurants: complete smoke-free law				3. Maternal age:<br>≤19 y: 0·2%§ (−0·1 to 0·5)<br>20−24 y: −0·1%§ (−0·2 to 0·0)<br>25−34 y: −0·1%§ (−0·2 to 0·0)<br>≥35 y: −0·1%§ (−0·2 to 0·0)		
	4. Restaurants: smoking restrictions (requiring designated smoking areas)				4. Maternal age:<br>≤19 y: −0·1%§ (−0·4 to 0·2)<br>20−24 y: −0·1%§		

					(−0·2 to 0·0)<br>25−34 y: −0·1%§ (−0·2 to 0·0)<br>≥35 y: 0·0%§ (−0·1 to 0·1)		
Peelen (2016)[30]*	1st smoke-free law: Workplaces and public transport except : restaurants and bars† (allowing designated smoking areas)	1,972,163	13,974	NA due to non-linear time trend	−6·0% (−13·9 to 3·0)	NA	National smoke-free workplaces and public transport were not associated with significant changes in VLBW. Expanding the smoke-free law to include restaurants and bars was also not associated with significant changes in VLBW.
	2nd smoke-free law: Expanding smoke-free law 1 to include restaurants and bars‡ (allowing designated smoking areas)				−6·0% (−14·9 to 3·0)		
**Extremely low birth weight**							
Bharadwaj (2014)[8]	Restaurants and bars (in addition to already existing laws in public places and workplaces)	822 (I); 3185 (C)	NR	NR	−0·3%§ (−1·7 to 1·1)	NA	Smoke-free restaurants and bars were not associated with significant changes in ELBW among women working in restaurants and bars.
**Small for gestational age**							
Bakolis (2016)[3]	Public places and workplaces (including restaurants and bars)	1,800,906	175,940 (10%)	NR	± 1 month: −8·2% (−13·7 to −1·8)<br>± 2 months: −5·4% (−10·0 to −0·9)<br>± 3 months: −6·4% (−10·0 to −2·7)<br>± 5 months: −4·5% (−7·3 to −1·8)	NA	National smoke-free legislation was associated with a reduction in SGA between 5% and 9% .
Cox (2013)[11]	Public places and workplaces (excluding catering industry)	606,877	59,799	NR	Single smoke-free law¶: −0·25% (−2·07 to 1·60)	Single smoke-free law¶: −3·20% (−6·93 to 0·68)	Smoke-free public places and workplaces were not associated with significant changes in SGA.
					Final model‖: no significant changes	Final model‖: no significant changes	
	Restaurants<br>(in addition to already existing smoke-free laws in public places and workplaces)	606,877	59,799	NR	Single smoke-free law¶: −0·82% (−2·53 to 0·92)	Single smoke-free law¶: −3·44% (−7·96 to 1·31)	Expanding smoke-free legislation to include restaurants was not associated with significant changes in SGA.
					Final model‖: no significant changes	Final model‖: no significant changes	

	Bars serving food (in addition to already existing smoke-free laws in public places and workplaces, including restaurants)	606,877	59,799	NR	Single smoke-free law¶: 0·32% (−1·95 to 2·65)	Single smoke-free law¶: 0·80% (−2·81 to 4·54)	Expanding smoke-free legislation to include bars was not associated with significant changes in SGA.
					Final model‖: no significant changes	Final model‖: no significant changes	
Hawkins (2014)[19]	100% smoke-free workplaces and restaurants	16,198,654	1,684,660	NR	−0·39%§ (−1·49 to 0·71)	NA	State-wide smoke-free workplaces and restaurants were not associated with significant changes in SGA.
Kabir (2013)[21]	Public places and workplaces (including restaurants and bars)	588,997	39,773	NR	−0·45% (−0·70 to −0·19)	−0·02% (−0·03 to −0·01)	Smoke-free public places and workplaces were associated with an immediate 0·5% decrease, and a subsequent 0·02% per year decrease in SGA.
Mackay (2012)[25]	Public places and workplaces (including restaurants and bars)	709,279	64,600	NR	Crude: −4·54% (−8·21 to −0·73)	Crude: −2·68% (−4·54 to −0·77)	Smoke-free public places and workplaces were associated with an immediate 4·5% decrease in SGA.
					Adjusted: −4·52% (−8·28 to −0·60)	Adjusted: −1·54 (−3·47 to 0·44)	
McKinnon (2015)[27]	Public places and workplace (including restaurants and bars)	470,136	37,948	NR	Crude: −8% (−11 to −4)	NA	State-wide smoke-free legislation was associated with a 7% decrease in SGA.
					Adjusted: −7 (−11 to −3)		
Peelen (2016)[30]*	1st smoke-free law: Workplaces and public transport except : restaurants and bars† (allowing designated smoking areas)	1,972,157	187,966	NA due to non-linear time trend	−0·9% (−2·7 to 0·9)	NA	National smoke-free workplaces and public transport were not associated with significant changes in the odds of SGA.<br>Expanding the smoke-free law to include restaurants and bars was associated with a 4% decrease in odds of SGA.
	2nd smoke-free law: Expanding smoke-free law 1 to include restaurants and bars‡ (allowing designated smoking areas)				−3·6% (−5·5 to −1·8)		
Simón (2017)[33]	1st smoke-free law: Complete smoke-free workplaces and partial smoke-free restaurants and bars	5,302,374	414,716 (7·8%)	NR	1st smoke-free law: –4·9% (–6·2 to –3·5)	NA	National partial smoke-free legislation was associated with a 5% decrease in SGA. The subsequent comprehensive smoke-free legislation was not associated with significant changes in SGA.
	2nd smoke-free law: Public places and workplaces (including restaurants and bars)				2nd smoke-free law: 0·7% (–0·8 to 2·2)		

**Very small for gestational age**							
Kabir (2013)[21]	Public places and workplaces (including restaurants and bars)	588,997	26,055	NR	−5·3% (−5·43 to −5·17)	−0·600% (−0·604 to −0·596)	Smoke-free public places and workplaces were associated with an immediate 5% decrease, and a subsequent 0·6% per year decrease in VSGA.
Mackay (2012)[25]	Public places and workplaces (including restaurants and bars)	709,279	14,460	NR	Crude: −7·82% (−14·95 to −0·09)	Crude: −3·03% (−6·85 to 0·94)	Smoke-free public places and workplaces were associated with an immediate 8% decrease in VSGA.
					Adjusted: −7·95% (−15·19 to −0·08)	Adjusted: −1·23% (−5·17 to 2·88)	
Peelen (2016)[30]*	1st smoke-free law: Workplaces and public transport except : restaurants and bars†[a] (allowing designated smoking areas)	1,972,157	46,195	NA due to non-linear time trend	2·0% (−2·0 to 5·9)	NA	National smoke-free workplaces and public transport were not associated with significant changes in the odds of very small for GA. Expanding the smoke-free law to include restaurants and bars was associated with an 8% decrease in odds of very small for GA.
	2nd smoke-free law: Expanding smoke-free law 1 to include restaurants and bars‡ (allowing designated smoking areas)				−7·8% (−10·8 to −3·9)		
**Congenital anomalies**							
Bharadwaj (2014)[8]	Restaurants and bars (in addition to already existing smoke-free laws in public places and workplaces)	822 (I); 3185 (C)	NR	NR	0·04%§ (−3·7 to 3·8)	NA	Smoke-free restaurants and bars were not associated with significant changes in congenital anomalies among women working in restaurants and bars.
Peelen (2016)[30]*	1st smoke-free law: Workplaces and public transport except : restaurants and bars† (allowing designated smoking areas)	1,983,761	19,412	NA due to non-linear time trend	1·0% (−6·0 to 8·0)	NA	Both policies were not associated with significant changes in the odds of developing congenital anomalies.
	2nd smoke-free law: Expanding smoke-free law 1 to include restaurants and bars‡ (allowing designated smoking areas)				−2·0% (−8·9 to 5·9)		
**Neonatal mortality**							
Been, Mackay (2015)[5]	Public places and workplaces (including restaurants and bars)	9,933,349	31,200	NA due to non-linear time trend	−7·6% (−11·7 to −3·4)	NA	National smoke-free legislation was associated with an immediate 8% reduction in neonatal mortality.
**Early neonatal mortality**							

Been, Mackay (2015)[5]	Public places and workplaces (including restaurants and bars)	9,933,349	23,929	NA due to non-linear time trend	−4·2% (−11·0 to 3·2)	NA	No evidence of an association between national smoke-free legislation and early neonatal mortality.
Peelen (2016)[30]*	1st smoke-free law: Workplaces and public transport except : restaurants and bars† (allowing designated smoking areas)	1,972,163	3,864	NA due to non-linear time trend	−3·0% (−16·0 to 12·0)	NA	Both policies were not associated with significant changes in the odds of developing early neonatal mortality.
	2nd smoke-free law: Expanding smoke-free law 1 to include restaurants and bars‡ (allowing designated smoking areas)				−12·0% (−24·0 to 2·0)		
**Late neonatal mortality**							
Been, Mackay (2015)[5]	Public places and workplaces (including restaurants and bars)	9,911,272	7,271	NA due to non-linear time trend	−13·7% (−20·7 to −6·0)	NA	National smoke-free legislation was associated with an immediate 14% reduction in late neonatal mortality.
**Post neonatal mortality**							
Been, Mackay (2015)[5]	Public places and workplaces (including restaurants and bars)	9,904,292	15,832	NA due to non-linear time trend	−4·6% (−10·0 to 1·0)	NA	No evidence of an association between national smoke-free legislation and post neonatal mortality.
**Infant mortality**							
Been, Mackay (2015)[5]	Public places and workplaces (including restaurants and bars)	9,933,349	47,032	NA due to non-linear time trend	−6·3% (−9·6 to −2·9)	NA	National smoke-free legislation was associated with an immediate 6% reduction in infant mortality.
Hajdu (2017)[17]	Public places and workplaces (including restaurants and bars)	18,755	NR	NR	–0·5%§ (–1·1 to 0·1)	NA	National smoke-free legislation was not associated with a significant change in infant mortality.
Vicedo-Cabrera (2017)[35]	Public places and workplaces (including restaurants and bars), with several exceptions in the hospitality sector‡‡	NR	1,160	NR	–16·6 (–42·5 to 21·1)	NA	Federal smoke-free legislation was not associated with a significant change in infant mortality.
**Child mortality**							
Shetty (2011)[32]	1. All workplaces except restaurants and bars: 100% smoke-free	NR	NR	NR	100% smoke-free workplaces: –0·7% (–5·4 to 4·1)	NA	No evidence for an association between smoking restrictions and child mortality (0−17 years old).
	2. Any smoke-free workplaces, restaurant, or bar law				Any smoke-free law: –0·7% (–4·9 to 3·6)		
**Wheezing/Asthma**							
Been, Szatkowski (2015)[7]	Public places and workplaces (including restaurants and bars) in 1. England	5,720,687 patient-years	294,034	NA due to non-linear time trends	England: −6% (−19 to 9)	NA	National smoke-free legislation was not associated with significant changes in GP wheezing/asthma diagnoses.
	Public places and workplaces (including restaurants and bars) in 2. Northern Ireland	228,850 patient-years	14,920		Northern Ireland: −4% (−24 to 22)		

	Public places and workplaces (including restaurants and bars) in 3. Scotland	661,212 patient-years	29,277		Scotland: −1% (−17 to 19)		
	Public places and workplaces (including restaurants and bars) in 4. Wales	540,925 patient-years	28,411		Wales: 9% (−11 to 35)		
**Respiratory infections**							
Been, Szatkowski (2015)[7]	Public places and workplaces (including restaurants and bars) in 1. England	7,620,464 patient-years	3,555,769	NA due to non-linear time trends	England: −5% (−14 to 6)	NA	National smoke-free legislation was not associated with significant changes in GP RTI diagnoses.
	Public places and workplaces (including restaurants and bars) in 2. Northern Ireland	339,015 patient-years	133,951		Northern Ireland: −10% (−21 to 3)		
	Public places and workplaces (including restaurants and bars) in 3. Scotland	852,750 patient-years	269,452		Scotland: −4% (−17 to 12)		
	Public places and workplaces (including restaurants and bars) in 4. Wales	723,773 patient-years	365,617		Wales: −3% (−14 to 9)		
**Upper respiratory infections**							
Been, Szatkowski (2015)[7]	Public places and workplaces (including restaurants and bars) in England	7,620,464 patient-years	3,452,915	NA due to non-linear time trend	England: −5% (−14 to 6)	NA	National smoke-free legislation was not associated with significant changes in GP URTI diagnoses.
**Lower respiratory infections**							
Been, Szatkowski (2015)[7]	Public places and workplaces (including restaurants and bars) in England	7,620,464 patient-years	115,633	NA due to non-linear time trend	England: −4% (−19 to 15)	NA	National smoke-free legislation was not associated with significant changes in GP LRTI diagnoses.

**MPOWER: Offer help to quit tobacco use (i.e. providing smoking cessation services)**							
**Gestational age**							
Adams (2013)[1]	1. Medicaid cessation coverage of: nicotine replacement therapy, medication, and counselling	Group 1 – enrolled in Medicaid before pregnancy: 52,372<br><br>Group 2 – entered Medicaid during pregnancy: 104,211<br><br>Group 3 – enrolled in Medicaid either before or during pregnancy: 151,938	Group 1: 38·99 wk (95%CI not given)<br><br>Group 2: 39·21 wk (95%CI not given)<br><br>Group 3: 39·15 wk (95%CI not given)	NR	Group 1: 0·018 wk (−0·080 to 0·116)<br><br>Group 2: 0·086 wk (0·004 to 0·168)<br><br>Group 3: 0·063 wk (0·008 to 0·118)	NA	Living in a state with Medicaid cessation coverage of nicotine replacement therapy, medication, and counselling was associated with a 0·09 wk increase in GA among women who entered Medicaid during pregnancy, and with a 0·06 wk increase in GA among women who enrolled in Medicaid either before or during pregnancy.<br><br>Living in a state with some Medicaid cessation coverage
	2. Medicaid cessation coverage of: nicotine replacement therapy and medication, but no counselling				Group 1: 0·123 wk (−0·026 to 0·272)<br><br>Group 2: 0·036 wk (−0·040 to 0·112)<br><br>Group 3: 0·057 wk		

					(−0·006 to 0·120)		was associated with a 0·09 wk increase in GA among women who entered Medicaid during pregnancy, and among women who enrolled in Medicaid either before or during pregnancy.
	3. Some Medicaid cessation coverage of nicotine replacement therapy, medication, and counselling, but not already represented in categories 1 or 2				Group 1: 0·096 wk (−0·049 to 0·241)<br><br>Group 2: 0·087 wk (0·016 to 0·158)<br><br>Group 3: 0·090 wk (0·017 to 0·163)		
**Birth weight**							
Adams (2013)[1]	1. Medicaid cessation coverage of: nicotine replacement therapy, medication, and counselling	Group 1 – enrolled in Medicaid before pregnancy: 57,283<br><br>Group 2 – entered Medicaid during pregnancy: 113,464<br><br>Group 3 – enrolled in Medicaid either before or during pregnancy: 165,686	Group 1: 3200 g (95%CI not given)<br><br>Group 2: 3273 g (95%CI not given)<br><br>Group 3: 3251 g (95%CI not given)	NR	Group 1: 10·18 g (−12·83 to 33·19)<br><br>Group 2: 16·20 g (−9·20 to 41·60)<br><br>Group 3: 12·36 g (−2·36 to 27·07)	NA	Expansion of Medicaid covered smoking cessation services were not associated with changes in birth weight.
	2. Medicaid cessation coverage of: nicotine replacement therapy and medication, but no counselling				Group 1: 24·33 g (−23·97 to 72·62)<br><br>Group 2: 19·17 g (−6·37 to 44·71)<br><br>Group 3: 22·67 g (−4·95 to 50·29)		
	3. Some Medicaid cessation coverage of nicotine replacement therapy, medication, and counselling, but not already represented in categories 1 or 2				Group 1: 9·60 g (−26·47 to 45·67)<br><br>Group 2: 9·34 g (−3·50 to 22·18)<br><br>Group 3: 11·93 g (−3·12 to 26·98)		
**Small for gestational age**							
Jarlenski (2014)[41]	State adoption of one of two optional Medicaid enrolment policies, allowing more low-income pregnant women to receive prenatal care, including smoking cessation services (presumptive eligibility and the unborn child option)**	24,544	NR	NR	Overall: −3·3%§ (−6·5 to 0·37)	NA	No statistically significant change in SGA following policy implementation.
					Comprehensive: 0·81%§ (−2·0 to 3·6)		
					Non-comprehensive: 2·1%§ (−1·9 to 6·1)		

**MPOWER: Raise taxes on tobacco**							
**Gestational age**							
Adams (2013)[1]	Cigarette price increase (in 2008 USD)	Group 1 – enrolled in Medicaid before pregnancy: 52,372	Group 1: 38·99 wk (95%CI not given)	NR	Group 1: −0·06 0wk (−0·289 to 0·169)	NA	A 1 USD increase in cigarette price was associated with a 0·10 wk increase in GA among women who entered Medicaid during pregnancy, and a 0·09 wk increase in GA among women who enrolled in Medicaid either before or during pregnancy.
		Group 2 – entered Medicaid during pregnancy: 104,211	Group 2: 39·21 wk (95%CI not given)		Group 2: 0·100 wk (0·035 to 0·165)		
		Group 3 – enrolled in Medicaid either before or during pregnancy: 151,938	Group 3: 39·15 wk (95%CI not given)		Group 3: 0·086 wk (0·023 to 0·149)		
Briggs (2009)[9]	Cigarette excise tax increase	State level: 64·3 million<br><br>County level: Unknown	State level: 34·8 million<br><br>County level: 9·8 million	NR	0·110 wk (0·003); α =0·001	NA	A 1 USD increase in cigarette excise tax was associated with a 0·11 week increase in GA.
Markowitz (2013)[26]	1. Cigarette excise tax increase (in 2008 USD)	Maternal age:<br>≤19 y: 54,132<br>20−24 y: 101,723<br>25−34 y: 183,763<br>≥35 y: 53,109	Maternal age:<br>≤19 y: 39·10 wk<br>20−24 y: 39·28<br>25−34 y: 39·26 wk<br>≥35 y: 39·01<br><br>(95%CI not given)	NR	Cigarette excise tax:<br>Maternal age:<br>≤19 y: −0·00 wk (−0·20 to 0·19)<br>20−24 y: 0·08 wk (−0·01 to 0·17)<br>25−34 y: 0·04 wk (−0·04 to 0·13)<br>≥35 y: 0·04 wk (−0·08 to 0·15)	NA	State-wide increase in cigarette excise tax or cigarette price was not associated with significant changes in GA.
	2. Cigarette price increase (in 2008 USD)				Cigarette price:<br>Maternal age:<br>≤19 y: −0·02 wk (−0·21 to 0·17)<br>20−24 y: 0·06 wk (−0·01 to 0·13)<br>25−34 y: 0·04 wk (−0·04 to 0·12)<br>≥35 y: 0·02 wk (−0·07 to 0·10)		
**Very preterm birth**							
Markowitz (2013)[26]	1. Cigarette excise tax increase (in 2008 USD)	Maternal age:<br>≤19 y: 54,132<br>20−24 y: 101,723<br>25−34 y: 183,763<br>≥35 y: 53,109	Maternal age:<br>≤19 y: 2165 (4%)<br>20−24 y: 2034 (2%)<br>25−34 y: 3675 (2%)<br>≥35 y: 1062 (2%)	NR	Cigarette excise tax:<br>Maternal age:<br>≤19 y: −1·2%§ (−2·1 to −0·3)<br>20−24 y: −0·3%§ (−0·6 to −0·0)<br>25−34 y: −0·1%§ (−0·5 to 0·3)<br>≥35 y: −0·4%§ (−0·8	NA	State-wide increase in cigarette excise tax was associated with a 1·2 percentage point decrease in VPTB among women aged ≤19, a 0·3 percentage point decrease among women aged 20-24, and a 0·4 percentage point decrease among women

					to −0·0)		aged ≥35.
	2. Cigarette price increase (in 2008 USD)		(95%CI not given)		Cigarette price:<br>Maternal age:<br>≤19 y: −0·8%§ (−1·6 to 0·0)<br>NR for other maternal age groups		
**Extremely preterm birth**							
Markowitz (2013)[26]	1. Cigarette excise tax increase (in 2008 USD)	Maternal age:<br>≤19 y: 54,132<br>20−24 y: 101,723<br>25−34 y: 183,763<br>≥35 y: 53,109	Maternal age:<br>≤19 y: 541 (1%)<br>20−24 y: 102 (1%)<br>25−34 y: NR (<1%)<br>≥35 y: 531 (1%)	NR	Cigarette excise tax:<br>Maternal age:<br>≤19 y: −0·4%§ (−0·7 to −0·1)<br>20−24 y: −0·1%§ (−0·2 to −0·0)<br>25−34 y: −0·0%§ (−0·1 to 0·1)<br>≥35 y: −0·1%§ (−0·2 to −0·0)	NA	State-wide increase in cigarette excise tax was associated with a 0·4 percentage point decrease in EPTB among women aged ≤19, and a 0·1 percentage point decrease among women aged 20-24 and ≥35. State-wide increase in cigarette price was associated with a 0·3 percentage point decrease in EPTB among women aged ≤19.
	2. Cigarette price increase (in 2008 USD)				Cigarette price:<br>Maternal age:<br>≤19 y: −0·3%§ (−0·6 to −0·0)<br>NR for other maternal age groups		
**Birth weight**							
Adams (2013)[1]	Cigarette price increase (in 2008 USD)	Group 1 – enrolled in Medicaid before pregnancy: 57,283	Group 1: 3200 g (95%CI not given)	NR	Group 1: 40·66 g (−3·83 to 85·15)	NA	Increase in state cigarette price was not associated with significant changes in BW.
		Group 2 – entered Medicaid during pregnancy: 113,464	Group 2: 3273 g (95%CI not given)		Group 2: 13·32 g (−5·88 to 32·52)		
		Group 3 – enrolled in Medicaid either before or during pregnancy: 165,686	Group 3: 3251 g (95%CI not given)		Group 3: 20·26 g (−0·73 to 41·24)		
Briggs (2009)[9]	Cigarette excise tax increase	State level: 64·3 million<br><br>County level: Unknown	State level: 34·8 million<br><br>County level: 9·8 million	NR	−0·021 g (0·633)	NA	Increase in state cigarette excise tax was not associated with changes in BW.
Evans (1999)[37]	Cigarette excise tax increase in USD cents	10,571,642	3363 g (580)	NR	0·21 g (2.83)<br><br>Note: T-statistics in parenthesis	NA	A 0.01 USD increase in cigarette excise tax was associated with a 0·21 g increase in BW.
Hawkins (2014)[19]	Cigarette excise tax increase (in December 2010 USD)	NA	3339 g	NR	White/Years of maternal education:<br>0−11 y: 5·41 g (1·92	NA	Cigarette taxes were associated with an increase in BW amongst white mothers

					to 8·89)<br>12 y: 2·00 g (−0·56 to 4·56)<br>13−15 y: 0·94 g (−0·21 to 2·09)<br>≥16 y: 0·05 g (−0·46 to 0·55)		with the least amount of education, and with black mothers with any level of education.
					Black/Years of maternal education:<br>0−11 y: 3·98 g (1·91 to 6·04)<br>12 y: 1·88 g (0·59 to 3·17)<br>13−15 y: 1·54 g (0·61 to 2·47)<br>≥16 y: 0·34 g (0·03 to 0·64)		
					Hispanic/Years of maternal education:<br>0−11 y: −0·34 g (−0·83 to 0·16)<br>12 y: 0·22 g (−0·21 to 0·64)<br>13−15 y: 0·37 g (−0·08 to 0·82)<br>≥16 y: 0·08 g (−0·09 to 0·25)		
					Asian/Pacific Islander/Years of maternal education:<br>0−11 y: −0·40 g (−1·14 to 0·34)<br>12 y: 0·19 g (−0·03 to 0·41)<br>13−15 y: 0·04 g (−0·17 to 0·25)<br>≥16 y: −0·03 g (−0·05 to 0·00)		
					American Indian/Alaskan Native/Years of maternal education:<br>0−11 y: 1·42 g (−2·79 to 5·63)<br>12 y: −0·38 g (−2·27 to 1·50)<br>13−15 y: −0·05 g		

					(−2·08 to 1·98)<br>≥16 y: 0·31 g (−0·38 to 1·01)		
Markowitz (2013)[26]	1. Cigarette excise tax increase (in 2008 USD)	Maternal age:<br>≤19 y: 54,132<br>20−24 y: 101,723<br>25−34 y: 183,763<br>≥35 y: 53,109	Maternal age:<br>≤19 y: 3182 g<br>20−24 y: 3275 g<br>25−34 y: 3372 g<br>≥35 y: 3368 g<br><br>(95%CI not given)	NR	Cigarette excise tax:<br>Maternal age:<br>≤19 y: 30·71 g (−7·63 to 69·05)<br>20−24 y: 21·13 g (−9·55 to 51·81)<br>25−34 y: 1·95 g (−14·67 to 18·57)<br>≥35 y: −10·06 g (−33·81 to 13·69)		State-wide increase in cigarette excise tax or cigarette price was not associated with significant changes in BW.
	2. Cigarette price increase (in 2008 USD)				Cigarette price:<br>Maternal age:<br>≤19 y: 20·93 g (−7·76 to 49·61)<br>20−24 y: 13·61 g (−12·29 to 39·52)<br>25−34 y: 4·54 g (−8·73 to 17·81)<br>≥35 y: −12·28 g (−28·55 to 3·98)		
**Low birth weight**							
Briggs (2009)[9]	Cigarette excise tax increase	State level: 64·3 million<br><br>County level: Unknown	State level: 34·8 million<br><br>County level: 9·8 million	NR	0·003 (0·000); α= 0·001	NA	A 1 USD increase in cigarette excise tax was associated with an increase of LBW by 0·3 percentage points.
Evans (1999)[37]	Cigarette excise tax increase in USD cents	10,571,642	629,013 (6%)	NR	OLS model:<br>−0·00%§ (−1.45)<br><br>Probit model:<br>−0·00%§ (−1.39)<br><br>Note: T-statistics in parenthesis	NA	Increase in cigarette excise tax was not associated with changes in LBW.
Hawkins (2014)[19]	Cigarette excise tax increase (in December 2010 USD)	16,198,654	890,926	NR	White/Years of maternal education:<br>0−11 y: −0·08%§ (−0·14 to −0·03)<br>12 y: −0·02%§ (−0·05 to 0·01)<br>13−15 y: −0·01%§ (−0·02 to 0·00)<br>≥16 y: −0·00%§ (−0·00 to 0·00)	NA	Cigarette taxes were associated with a decrease in LBW amongst white mothers with the least amount of education, and with black mothers with the least amount of education.

					Black/Years of maternal education:<br>0−11 y: −0·12%§ (−0·17 to −0·06)<br>12 y: −0·05%§ (−0·08 to −0·01)<br>13−15 y: −0·03%§ (−0·05 to −0·01)<br>≥16 y: −0·01%§ (−0·01 to −0·00)		
					Hispanic/Years of maternal education:<br>0−11 y: 0·00%§ (−0·00 to 0·01)<br>12 y: −0·00%§ (−0·01 to 0·00)<br>13−15 y: −0·00%§ (−0·01 to 0·00)<br>≥16 y: −0·00%§ (−0·00 to 0·00)		
					Asian/Pacific Islander/Years of maternal education:<br>0−11 y: 0·01%§ (−0·01 to 0·03)<br>12 y: −0·00%§ (−0·01 to 0·00)<br>13−15 y: −0·00%§ (−0·00 to 0·00)<br>≥16 y: 0·00%§ (−0·00 to 0·00)		
					American Indian/Alaskan Native/Years of maternal education:<br>0−11 y: −0·02%§ (−0·07 to 0·04)<br>12 y: 0·00%§ (−0·01 to 0·02)<br>13−15 y: 0·00%§ (−0·02 to 0·02)<br>≥16 y: −0·00%§ (−0·01 to 0·00)		
Markowitz (2013)[26]	1. Cigarette excise tax increase (in 2008 USD)	Maternal age:<br>≤19 y: 54,132<br>20−24 y: 101,723<br>25−34 y: 183,763<br>≥35 y: 53,109	Maternal age:<br>≤19 y: 4872 (9%)<br>20−24 y: 7121 (7%)	NR	Cigarette excise tax:<br>Maternal age:<br>≤19 y: −0·8%§ (−2·4 to 0·8)<br>20−24 y: −0·2%§	NA	State-wide increase in cigarette excise tax was not associated with significant changes in LBW. State-wide increase in cigarette price was

			25−34 y: 9188 (5%)<br>≥35 y: 3718 (7%)		(−0·9 to 0·5)<br>25−34 y: −0·1%§ (−0·5 to 0·3)<br>≥35 y: 0·2%§ (−0·5 to 0·9)		associated with a 1·1 percentage point decrease in LBW among women aged ≤19.
	2. Cigarette price increase (in 2008 USD)				Cigarette price:<br>Maternal age:<br>≤19 y: −1·1%§ (−2·2 to −0·0)<br>NR for other maternal age groups		
Sen (2011)[40]	Cigarette tax increase (in Canadian dollars)	NR	5%	NR	GLS: 0·06%§ (−0·02 to 0 ·14))<br>OLS: 0·08%§ (−0·08 to 0·24)	NA	Provincial increase in cigarette tax was not associated with changes in LBW.
**Very low birth weight**							
Evans (1999)[37]	Cigarette excise tax increase in USD cents	10,571,642	107,831 (1%)	NR	OLS model: 0·00%§ (0.99)<br><br>Probit model: 0.00%§ (1.54)<br><br>Note: T-statistics in parenthesis	NA	Increase in cigarette excise tax was not associated with changes in VLBW.
Markowitz (2013)[26]	1. Cigarette excise tax increase (in 2008 USD)	Maternal age:<br>≤19 y: 54,132<br>20−24 y: 101,723<br>25−34 y: 183,763<br>≥35 y: 53,109	Maternal age:<br>≤19 y: 541 (1%)<br>20−24 y: 1,017 (1%)<br>25−34 y: 1,838 (1%)<br>≥35 y: 531 (1%)	NR	Cigarette excise tax:<br>Maternal age:<br>≤19 y: −0·2%§ (−0·6 to 0·2)<br>20−24 y: −0·0% (−0·1 to 0·1)<br>25−34 y: −0·0%§ (−0·1 to 0·1)<br>≥35 y: 0·0%§ (−0·1 to 0·2)	NA	State-wide increase in cigarette excise tax was not associated with significant changes in VLBW. State-wide increase in cigarette price was associated with a 0·2 percentage point decrease in VLBW among women aged ≤19.
	2. Cigarette price increase (in 2008 USD)				Cigarette price:<br>Maternal age:<br>≤19 y: −0·2%§ (−0·4 to −0·0)<br>NR for other maternal age groups		
**Small for gestational age**							
Hawkins (2014)[19]	Cigarette excise tax increase (in December 2010 USD)	16,198,654	1,684,660	NR	White/Years of maternal education:<br>0−11 y: −0·31%§ (−0·51 to −0·11)<br>12 y: −0·09%§	NA	Cigarette taxes were associated with a decrease in SGA amongst black mothers and among white mothers with the least amount of education,

					(−0·21 to 0·03)<br>13−15 y: −0·04%§ (−0·08 to 0·01)<br>≥16 y: −0·00 (−0·02 to 0·02)		and Hispanic and Asian/Pacific Islanders of medium education.
					Black/Years of maternal education:<br>0−11 y: −0·30%§ (−0·46 to −0·15)<br>12 y: −0·14%§ (−0·23 to −0·04)<br>13−15 y: −0·10%§ (−0·16 to −0·04)<br>≥16 y: −0·02 (−0·04 to −0·00)		
					Hispanic/Years of maternal education:<br>0−11 y: 0·02%§ (−0·01 to 0·04)<br>12 y: −0·01%§ (−0·03 to 0·01)<br>13−15 y: −0·02%§ (−0·04 to 0·00)<br>≥16 y: −0·00%§ (−0·01 to 0·00)		
					Asian/Pacific Islander/Years of maternal education:<br>0−11 y: 0·04%§ (−0·03 to 0·10)<br>12 y: −0·01%§ (−0·03 to 0·00)<br>13−15 y: −0·00%§ (−0·02 to 0·01) (<br>≥16 y: 0·00%§ (−0·00 to 0·00)		
					American Indian/Alaskan Native/Years of maternal education:<br>0−11 y: −0·07%§ (−0·27 to 0·14)<br>12 y: 0·02%§ (−0·06 to 0·10)<br>13−15 y: 0·00%§ (−0·08 to 0·09)<br>≥16 y: −0·01%§		

					(−0·04 to 0·02)		
**Stillbirth**							
Sen (2011)[40]	Cigarette tax increase (in Canadian dollars)	NR	6·9%	NR	GLS: 0·23%§ (0·03 to 0·43)	NA	A 10% increase in cigarette taxes was associated with a 0·9 to 1·6 percentage increase in fetal loss.††
					OLS: 0·43%§ (0·12 to 0·74)		
**Infant mortality**							
Patrick (2016)[39]	1. Cigarette excise tax increase (in 2010 USD)	NR	NR	NR	1. Crude: −0·041%§ (−0·049 to −0·032)	NA	Both a 1 USD mean state cigarette excise tax increase per pack, as well as a 1 USD mean state cigarette price increase per pack were associated with a 0·02 percentage point decrease in infant mortality.
					1. Adjusted: −0·019%§ (−0·033 to −0·005)		
	2. Cigarette price increase (in 2010 USD)				2. Crude: −0·034%§ (−0·040 to −0·027)		
					2. Adjusted: −0·019%§ (−0·030 to −0·009)		
Sen (2011)[40]	Cigarette tax increase (in Canadian dollars)	NR	1%	NR	GLS: −0·05%§ (−0·07 to −0·03)	NA	A 10% increase in cigarette taxes was associated with a 1·7 to 1·8% decrease in infant mortality.††
					OLS: −0·05%§ (−0·09 to −0·01)		
**Asthma**							
Bhai (2015)[36]	1. Cigarette excise tax increase (in 2007 USD)	289,210	Mean: 13·7% Standard deviation: 34·4	NR	−1·5%§ (−2·1 to −0·9)	NA	A 1USD increase in state cigarette excise tax in-utero was associated with a −1·5 percentage point decrease in child asthma prevalence. This association was particularly strong in states with low initial cigarette excise tax.
	2. Cigarette excise tax increase (in 2007 USD) in states with high cigarette excise tax				−0·4%§ (−1·4 to 0·6)		
	3. Cigarette excise tax increase (in 2007 USD) in states with low cigarette excise tax				−3·6%§ (−6·0 to −1·2)		

ARIMA=autoregressive integrated moving average; BW=birth weight; C=control group; CI=confidence interval; ELBW=extremely low birth weight; EPTB=extremely preterm birth; g=grams; GA=gestational age; GP=general practice; I=intervention group; LBW=low birth weight; LRTI=lower respiratory tract infection; NA=not applicable; NR=not reported; p=percentile; RTI=respiratory tract infection; SD=standard deviation; SGA=small for gestational age; URTI=upper respiratory tract infection; USD=US dollar; VLBW=very low birth weight; VPTB=very preterm birth; VSGA=very small for gestational age; wk=weeks

* Both smoke-free laws were accompanied by a tobacco tax increase and mass-media campaign.
† Exceptions to this smoke-free law were: hotels, bars and restaurants, sports, arts and culture venues, amusement arcades, tobacconist shops, international passenger transport, private spaces, open air, and designated areas for smoking within each facility.
‡ The smoke-free law now included hospitality venues: hotels, bars and restaurants, sports, art and culture venues, amusement arcades, tobacconist shops, and international passenger transport. Designated smoking areas within each facility were still allowed.

§ Percentage point change
¶The single smoke-free law model includes either the step or slope change of a single smoke-free law into the model.
||The final was obtained by including all three step changes and all three slope changes in one model and removing the least significant factors one at a time.
** Presumptive eligibility: low-income pregnant women are presumed to be eligible for Medicaid, so they can receive care (including smoking cessation services) while their Medicaid applications are still pending. The unborn-child option: the state can consider a foetus a “targeted low-income child”, allowing coverage of prenatal care (including smoking cessation services) and delivery to low-income pregnant women, even if they cannot provide documentation of citizenship or residency.
†† The authors report these estimates as the lagged tax elasticities calculated from the GLS and OLS models respectively.
‡‡ Authorised smoking in establishments <80m2 and designated smoking areas in larger establishments.

Figure S5: Meta-analysis of immediate changes in secondary outcomes after implementation of smoke-free legislation

A – Stillbirth

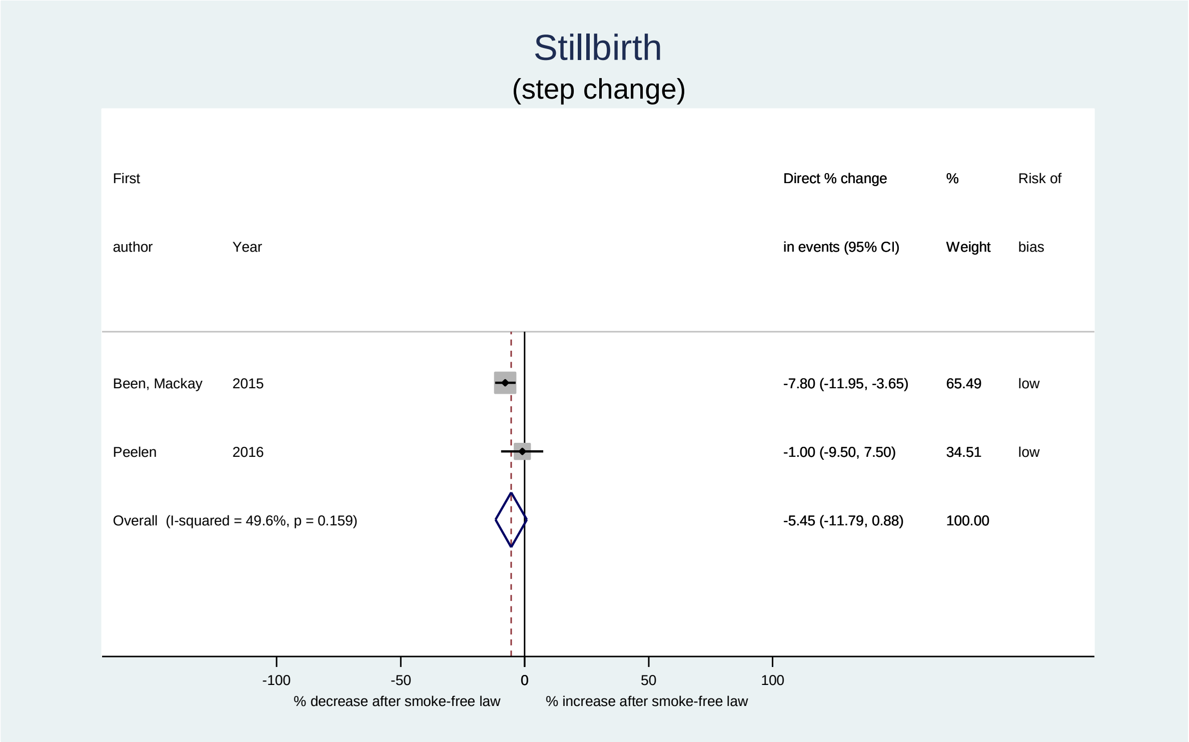

B – Gestational age

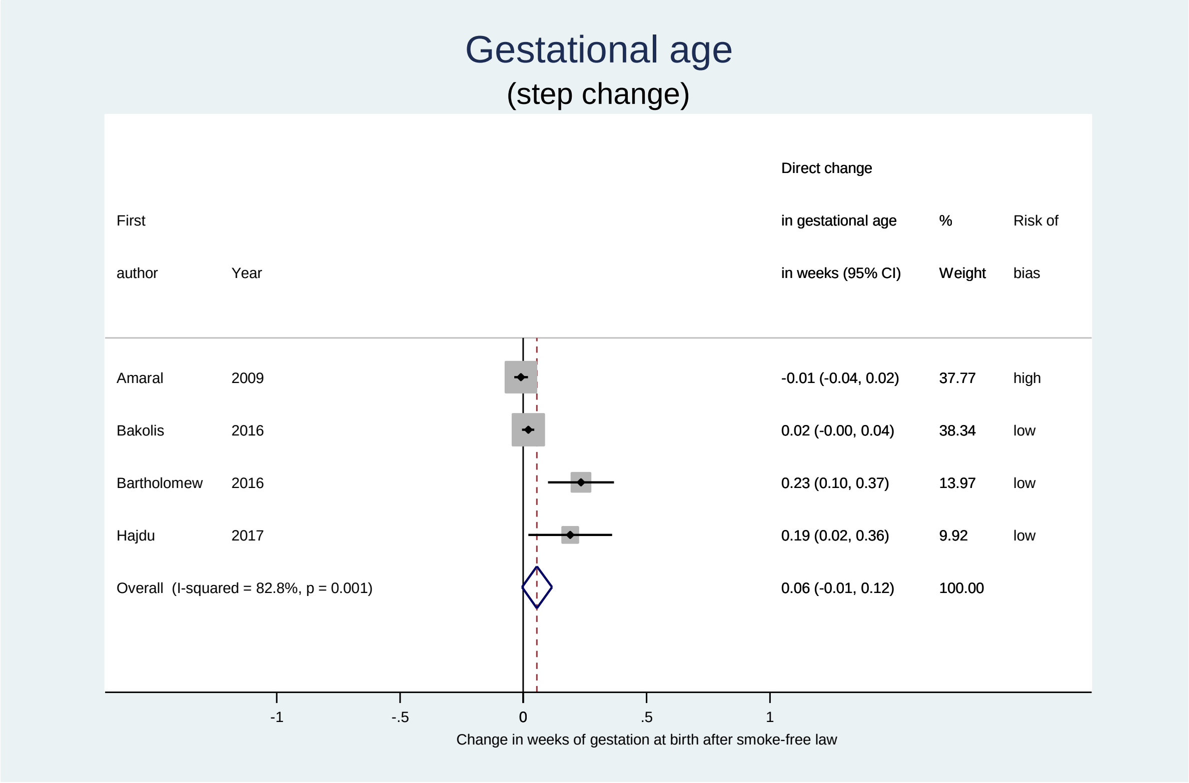

C – Very preterm birth

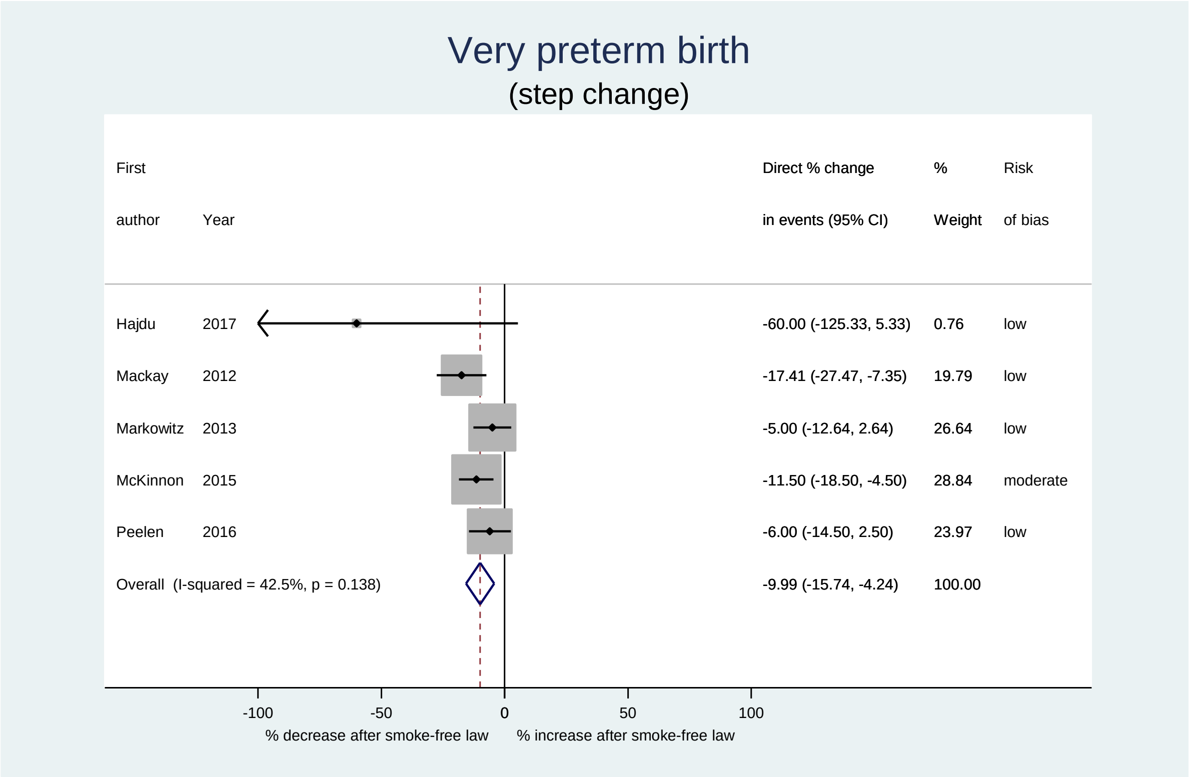

D – Birth weight

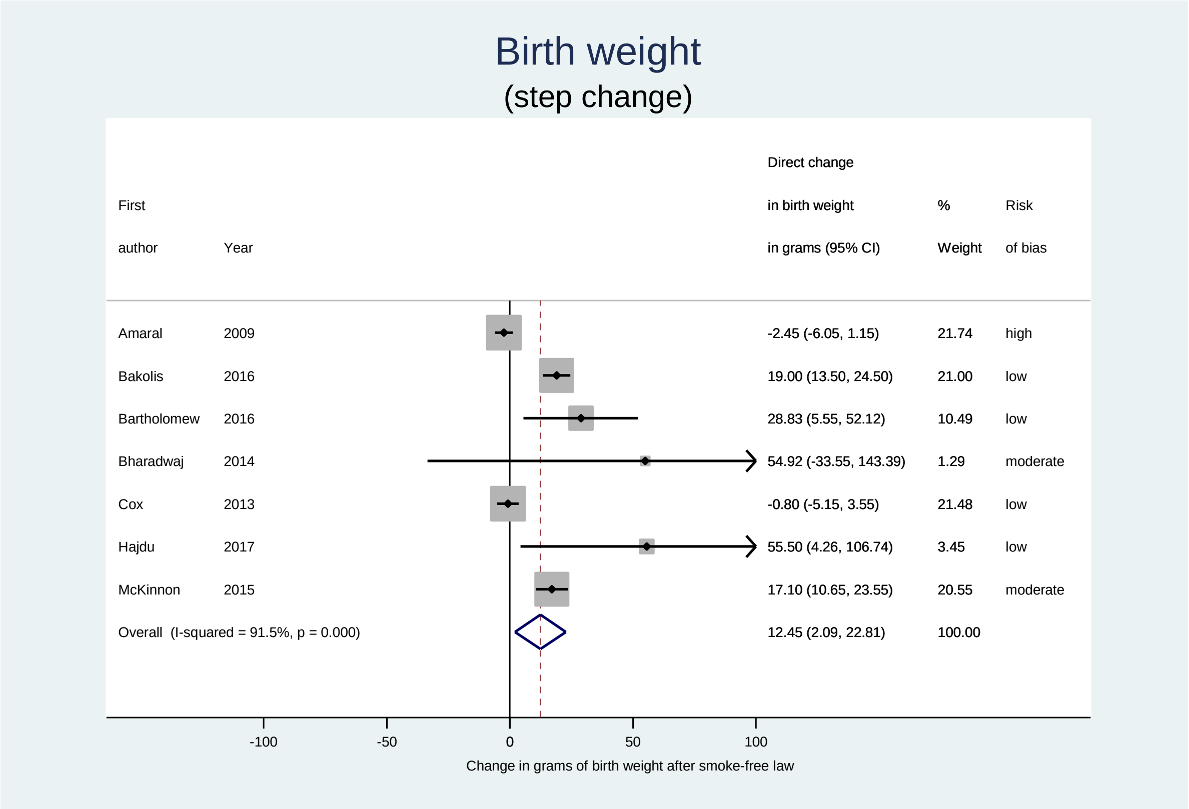

E – Low birth weight

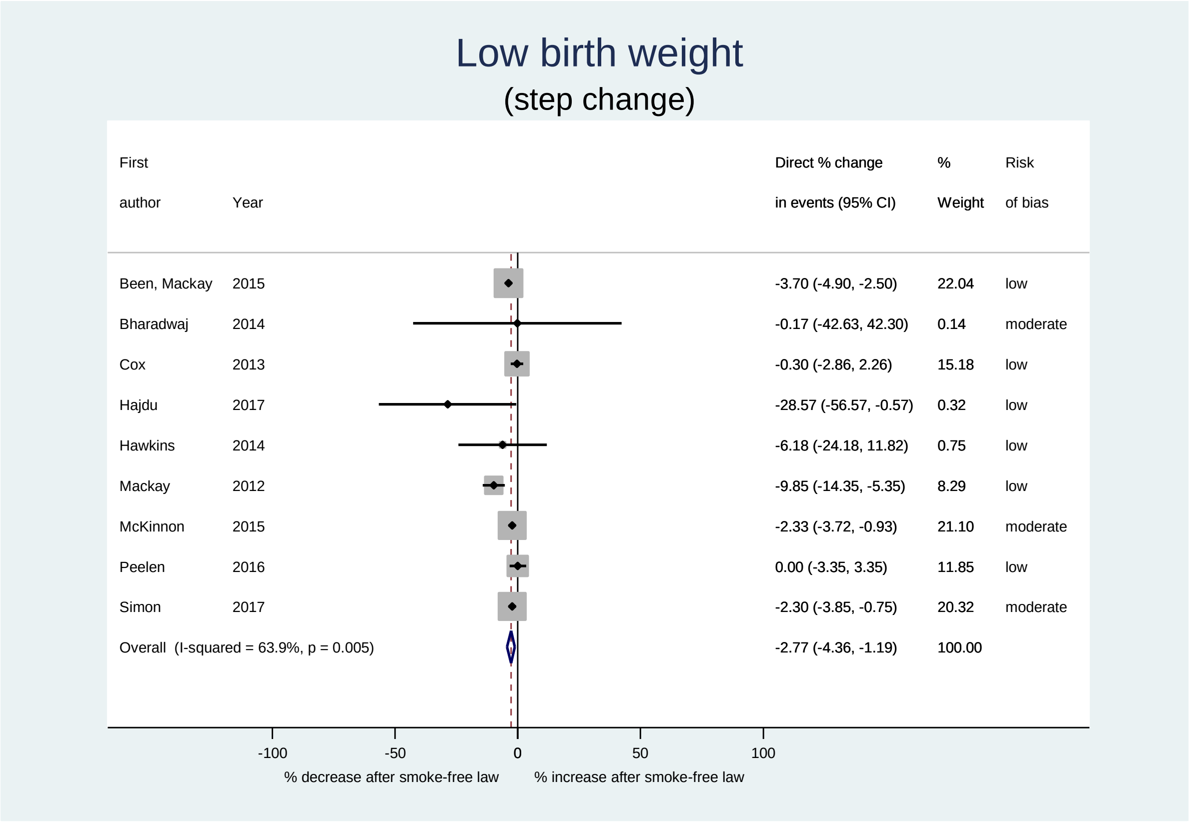

F – Very low birth weight

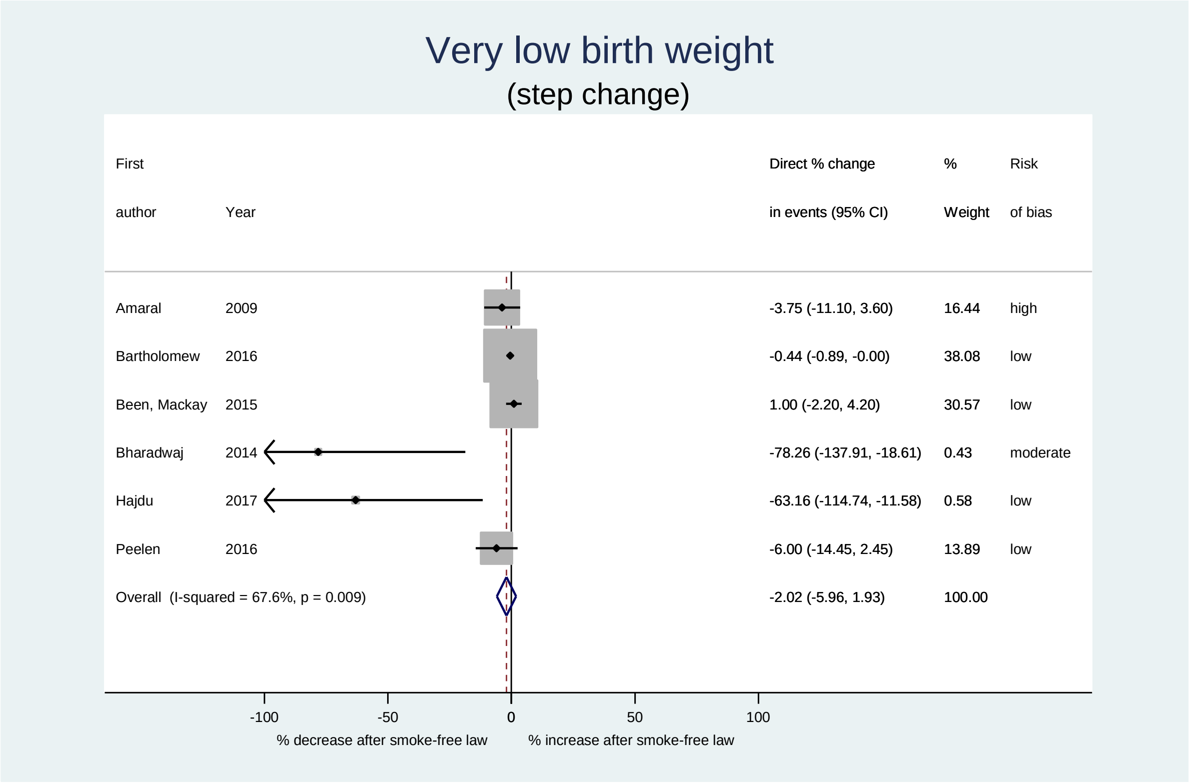

G – Small for gestational age

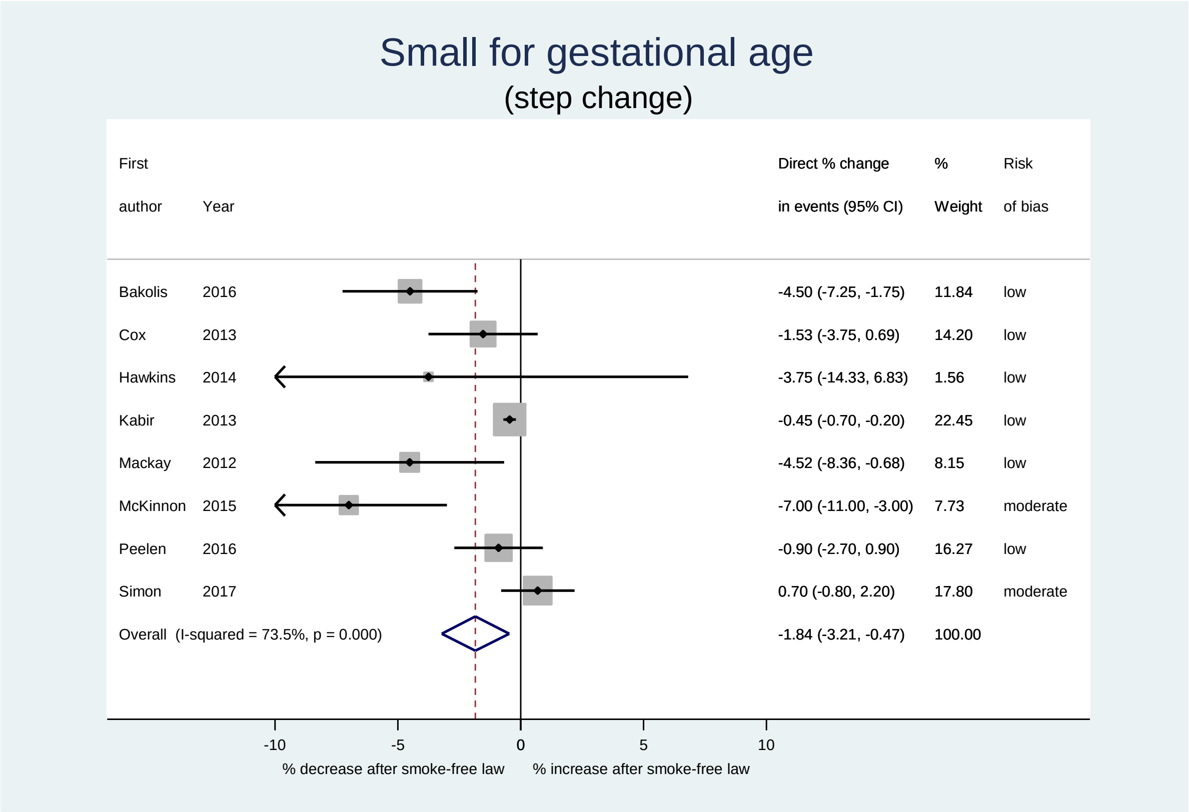

H – Very small for gestational age

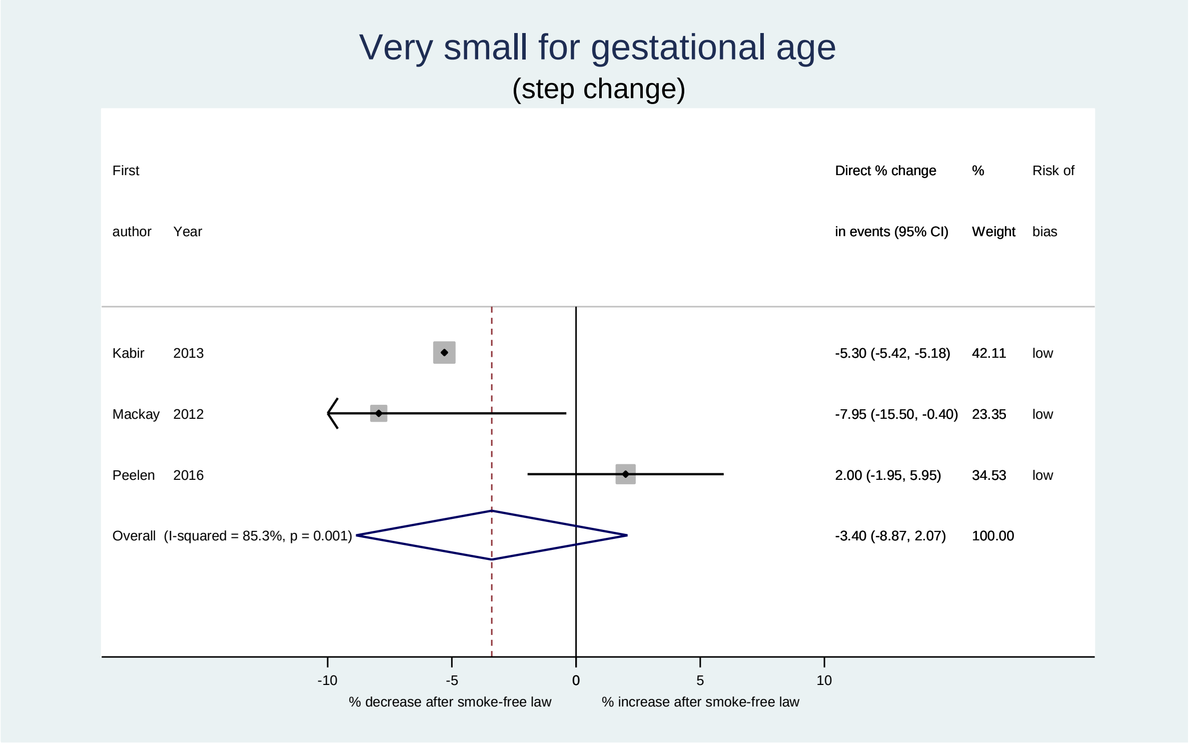

I – Congenital anomalies

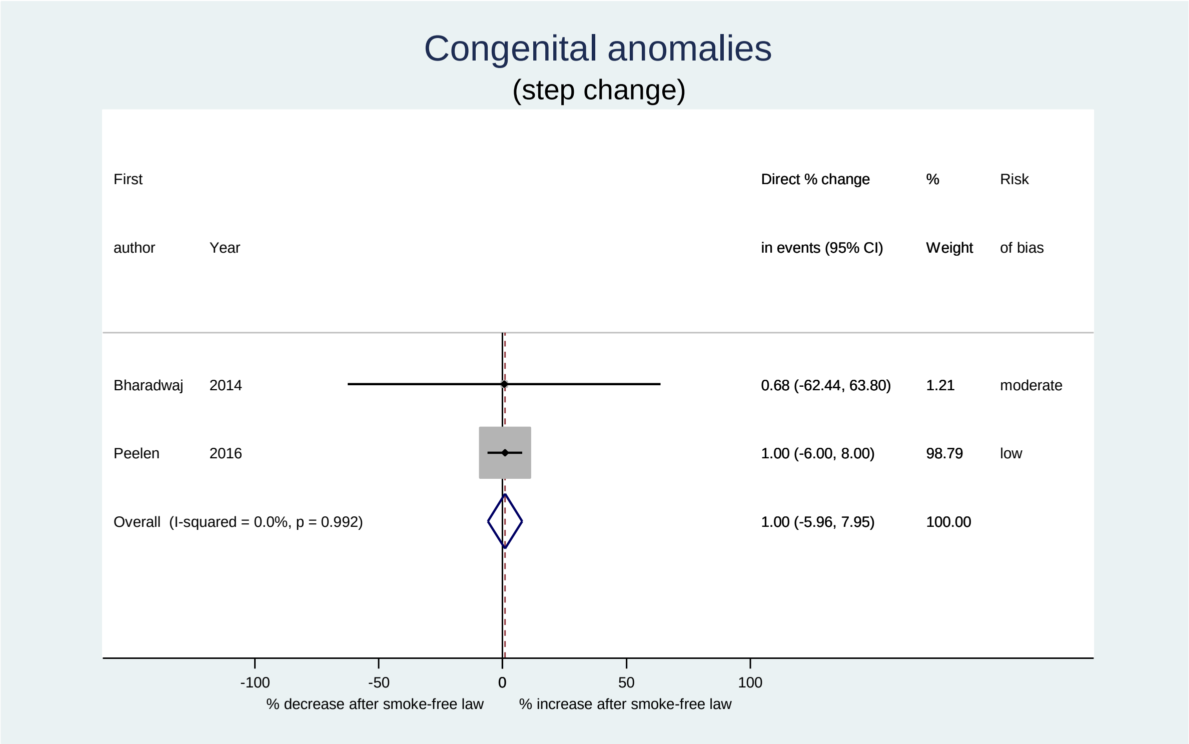

J – Early neonatal mortality

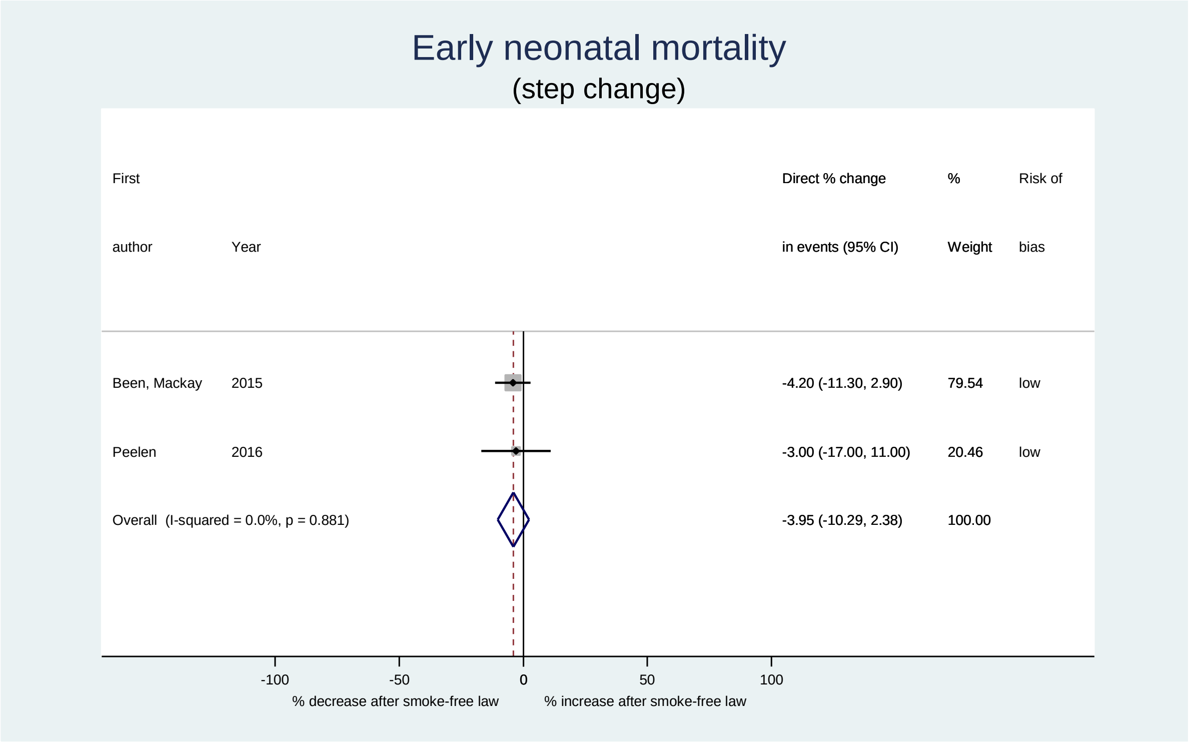

K – Infant mortality

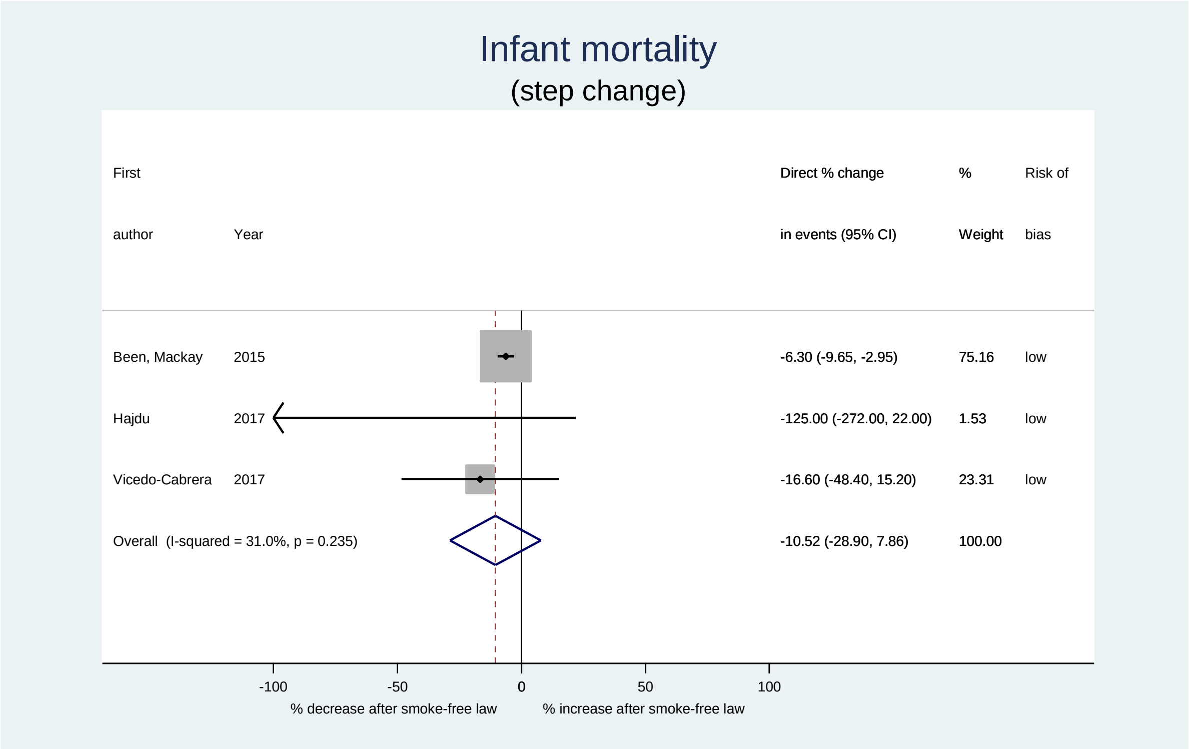

L – Wheezing/Asthma

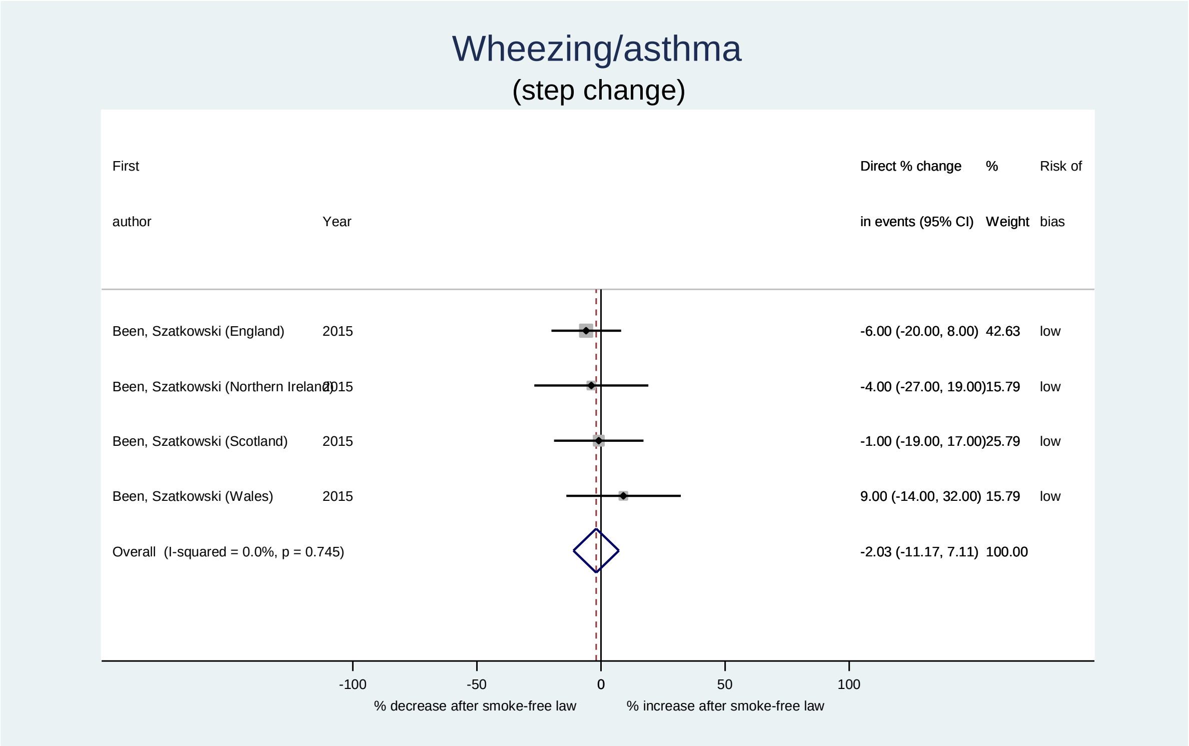

M – Respiratory infections

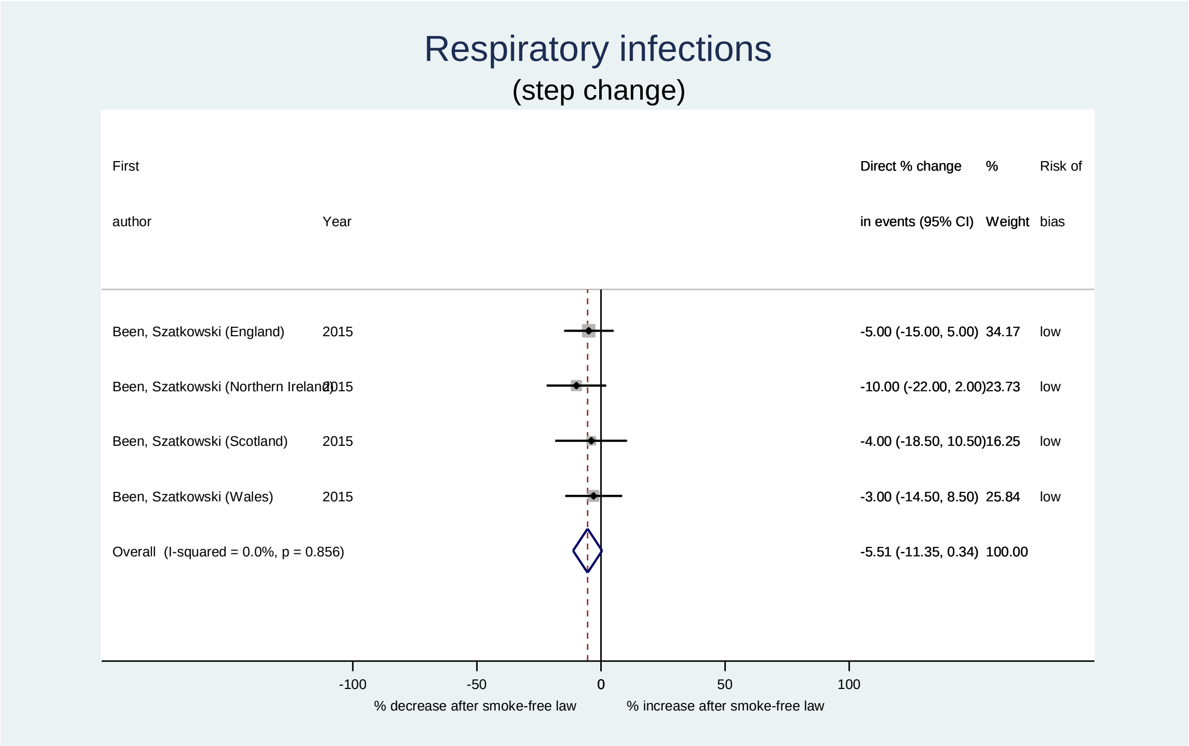

Figure S6: Meta-analysis of gradual changes in secondary outcomes after implementation of smoke-free legislation

A – Low birth weight

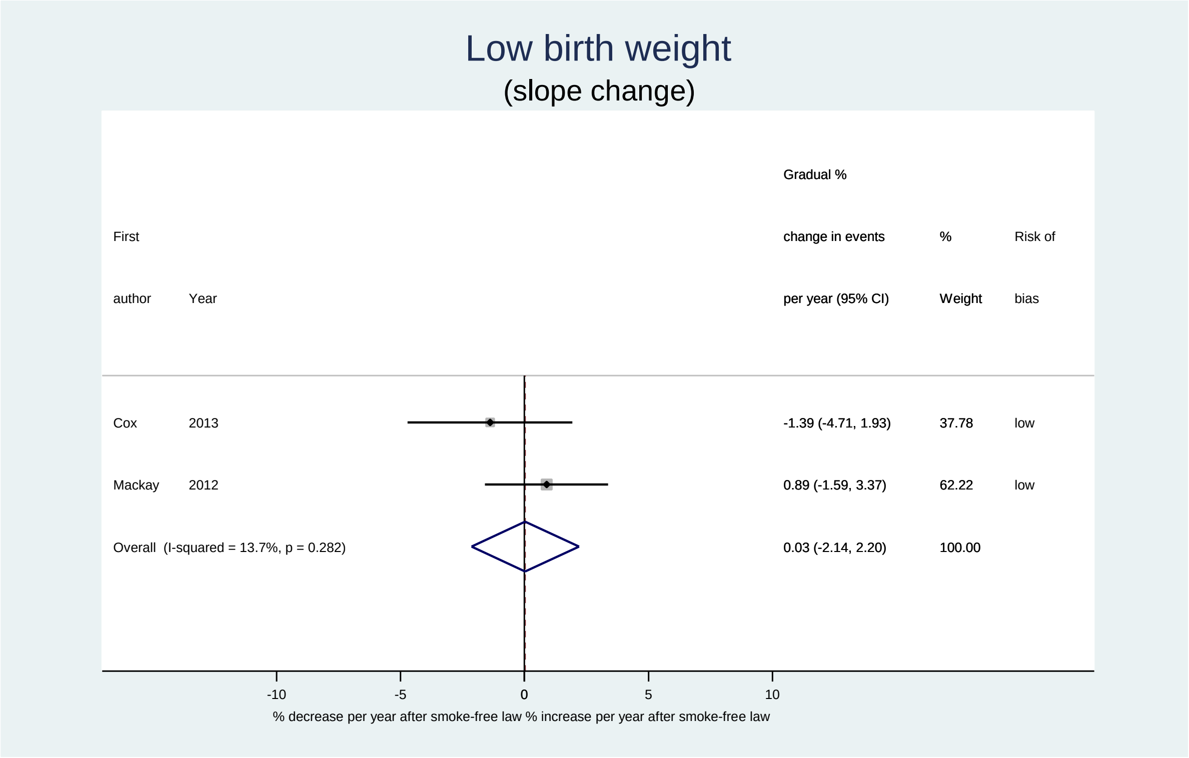

B – Small for gestational age

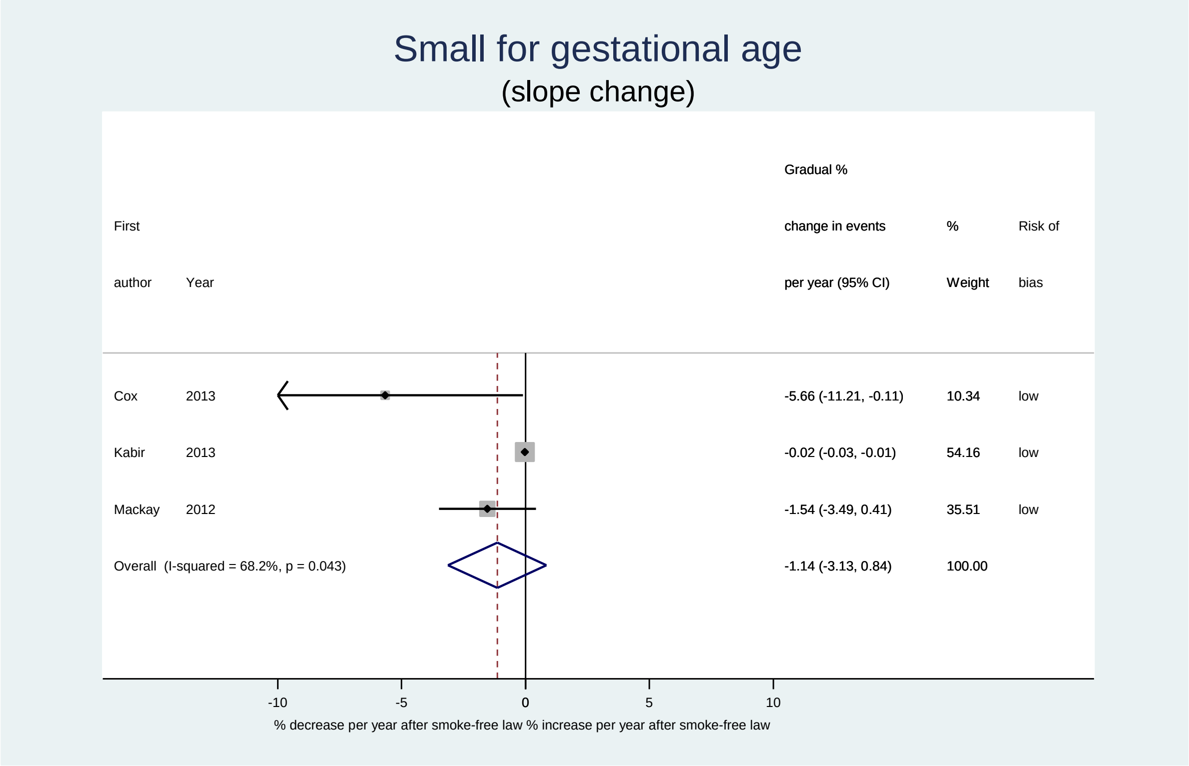

C – Very small for gestational age

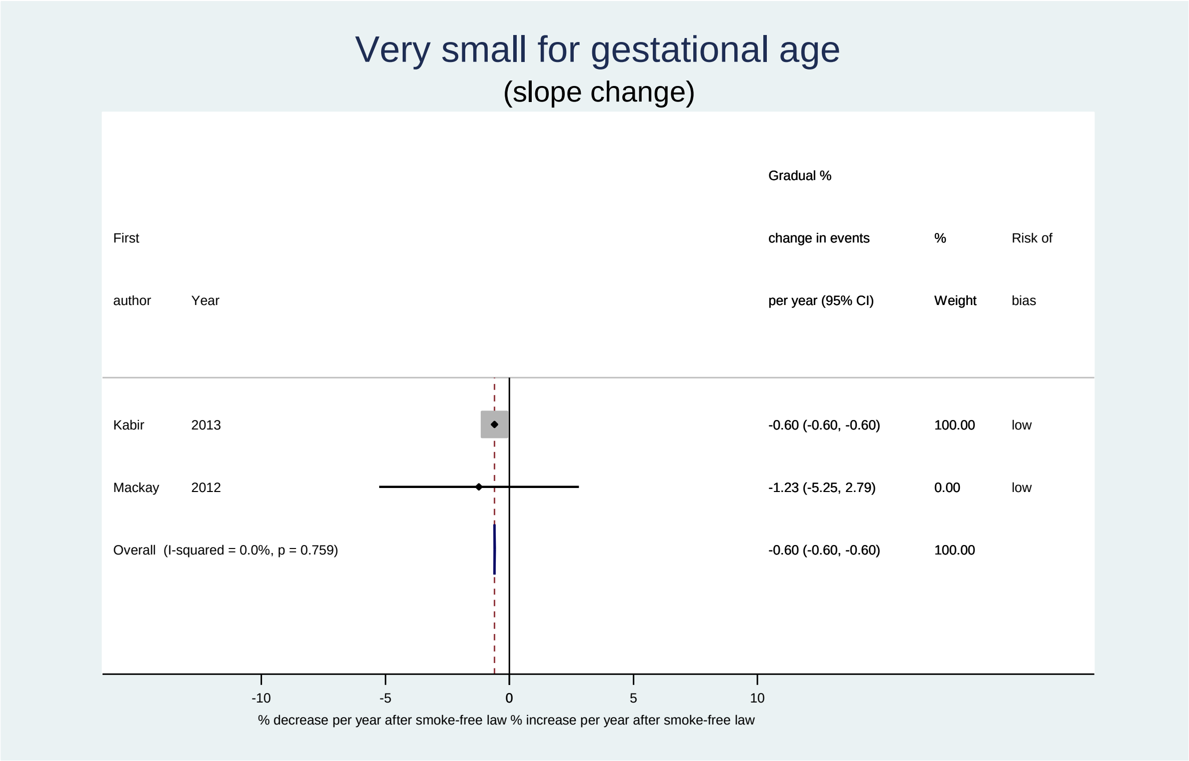

Table S8: Association between tobacco control policies and smoking behaviour in included studies

Study	Definition of smoking behaviour	Data source	Effect estimates	Summary of findings
**Maternal smoking before pregnancy**				
**Adams (2013)[1]**	Self-reported maternal smoking before pregnancy, during pregnancy, and after delivery.	PRAMS	1. Medicaid cessation coverage of: nicotine replacement therapy, medication, and counselling<br>Group 1 – enrolled in Medicaid before pregnancy: −2·5%* (−5·2 to 0·2)	Living in a state with Medicaid cessation coverage of nicotine replacement therapy and medication, but no counselling was associated with a −1·6 percentage point decrease in maternal smoking before pregnancy.<br><br>Living in a state with some Medicaid cessation coverage of nicotine replacement therapy, medications, and counselling was associated with a −3·0 percentage point decrease in maternal smoking before pregnancy.
			2. Medicaid cessation coverage of: nicotine replacement therapy and medication, but no counselling<br>Group 1 – enrolled in Medicaid before pregnancy: −1·6%* (−3·2 to −0·0)	
			3. Some Medicaid cessation coverage of nicotine replacement therapy, medications, and counselling not already represented in categories 1 or 2<br>Group 1 – enrolled in Medicaid before pregnancy: −3·0%* (−5·0 to −1·0)	
**Maternal smoking during pregnancy**				
**Adams (2013)[1]**	Self-reported maternal smoking before pregnancy, during pregnancy, and after delivery.	PRAMS	1. Medicaid cessation coverage of: nicotine replacement therapy, medication, and counselling<br>Group 2 – entered Medicaid during pregnancy: −0·4%* (−2·2 to 1·4)<br><br>Group 3 – enrolled in Medicaid either before or during pregnancy: −0·7%* (−2·7 to 1·3)	Expansion of Medicaid covered smoking cessation services were not associated with significant changes in maternal smoking during pregnancy.
			2. Medicaid cessation coverage of: nicotine replacement therapy and medication, but no counselling<br>Group 2 – entered Medicaid during pregnancy: 0·6%* (−0·8 to 2·0)<br><br>Group 3 – enrolled in Medicaid either before or during pregnancy: 0·6%* (−0·4 to 1·6)	
			3. Some Medicaid cessation coverage of nicotine replacement therapy, medications, and counselling not already represented in categories 1 or 2<br>Group 2 – entered Medicaid during pregnancy: 1·7%* (−1·4 to 4·8)<br><br>Group 3 – enrolled in Medicaid either before	

			or during pregnancy: 1·1%* (−0·9 to 3·1)	
Bartholomew (2016)[4]	Self-reported maternal smoking status at time of delivery.	West Virginia Vital Statistics	Smoke-free legislation:<br>Comprehensive: −0·015%* (−0·041 to 0·011)<br>Restrictive: 0·003%* (−0·005 to 0·011)<br>Moderate: 0 ·004%* (−0 ·002 to 0·010)<br>Limited: 0·001%* (−0·006 to 0·007)	No significant association was seen between any of the smoke-free regulations (comprehensive, restrictive, moderate or limited) and maternal smoking during pregnancy.
Bharadwaj (2014)[8]	Quit smoking during pregnancy, based on self-reported maternal smoking status at the start of pregnancy (GW 8-12) and at time of hospital delivery.	Medical Birth Registry of Norway	15·0%* (0·9 to 29·1)	After implementation of smoke-free restaurants and bars, there was a 15 percentage point increase in the proportion of mothers working in restaurants and bars who quit smoking during pregnancy as compared to mothers working in shops.
Evans (1999)[37]	Self-reported smoking and number of cigarettes smoked per day.	Natality Detail File	Maternal smoking<br>Probit model: -0.085%* (-14.68)<br>OLS model: -0.035%* (-7.36)<br><br>Note: T-statistics in parenthesis	The authors report that their results suggest that maternal smoking participation during pregnancy and daily number of cigarettes decline when cigarette excise taxes are increased, but it is unclear whether these changes are statistically significant.
			Daily number of cigarettes<br>Probit model: -0.0017 (-2.29)<br>OLS model: -0.0045 (-1.63)<br><br>Note: T-statistics in parenthesis	
Hankins (2016)[18]	Self-reported maternal smoking and number of cigarettes smoked per day.	National Center for Health Statistics; Centers for Disease Control and Prevention	Maternal smoking<br>Workplaces: −0·24%* (−0·67 to 0·19)<br>Restaurants: −0·08%* (−0·88 to 0·72)<br>Bars: −0·71%* (−1·57 to 0·15)	There was no association between smoke-free legislation and smoking behaviour of pregnant women. Smoke-free restaurants were associated with a −19 percentage points reduction in the daily number of cigarettes smoked during pregnancy.
			Daily number of cigarettes<br>Workplaces: −11·43%* (−0·74 to 23·60)<br>Restaurants: −19·01%* (−36·6 to −1·41)<br>Bars: −6·00%* (−24·66 to 12·66)	
Hawkins (2014)[19]	Self-reported maternal tobacco use during pregnancy.	US Natality Files; Public Health Statistics and Information Systems	Smoke-free workplaces/restaurants:<br>−0·91%* (−4·80, 2·98)	Smoke-free workplaces/restaurants were not associated with a significant change in maternal smoking during pregnancy.
			White mothers<br>0-11 years of maternal education: −2·35%* (–3·84 to –0·86)<br>12 years of maternal education: −0·83%* (–1·73 to 0·07)<br><br>Black mothers<br>0-11 years of maternal education: −2·09%* (–3·40 to –0·78)<br>12 years of maternal education: −0·90%* (–1·39 to –0.41)<br>13-15 years of maternal education: −0·70%* (–1·03 to –0·37)<br>16+ years of maternal education: −0·14%* (–	Increase in cigarette tax was not associated with a reduction in maternal smoking during pregnancy for all ethnicity and education subgroups. Increased cigarette taxes were associated with a reduction in maternal smoking during pregnancy when examined according to race/ethnicity and education, with significant reduction in smoking during pregnancy observed for white mothers with a high school degree or less, and amongst black mothers across all education levels.

			0·28 to –0·00)<br><br>None of the other subgroups were associated with changes in smoking during pregnancy (Hispanic, Asian/Pacific Islander, and American Indian/Alaskan Native).	
Jarlenski (2014)[41]	Self-reported maternal smoking 3 months prior to conception and during pregnancy.	PRAMS	Change in smoking cessation:<br><br>Either policy: 6·8%* (3·0 to 10·5)<br><br>Presumptive eligibility: 7·7%* (3·7 to 11·6)<br>Unborn-child option −2·1%* (−7·2 to 3·0)	State's adoption of presumptive eligibility (A policy which permits women to receive prenatal care whilst their Medicaid application is pending) was associated with a 7·7 percentage points increase in the probability of smoking cessation. Adopting the unborn-child option (a policy which permits states to provide coverage to pregnant women who cannot document their citizenship or residency) did not significantly affect cessation of maternal smoking during pregnancy. Having either enrolment policy in place was associated with a 6·8 percentage-point increase in the probability of maternal smoking during pregnancy.
Kabir (2009)[44]	Maternal smokers classified as never, former or current.	Euroking K2 maternity system (Coombe University Maternal Hospital)	Change in current smokers: Pre-ban 23·4% (95% CI: 22·5% to 24·4%), Post-ban 20·6% (95% CI: 19·7% to 21·6%)	Significant 12% relative reduction in maternal smoking rates following workplace smoking ban.
Mackay (2012)[25]	Maternal smokers self-classified as current, never and former smokers.	Scottish Morbidity Record 02	Smoking prevalence pre-ban: 25·4%,<br>Smoking prevalence post-ban: 18·8%	Following the introduction of smoke-free legislation, current smokers reduced from 25·4% to 18·8% (p<0·001) and there was an increase in never smoked from 57·3% to 58·4% (p<0·001).
Markowitz (2013)[26]	Self-reported maternal smoking during the last 3 months of pregnancy.	PRAMS	Mothers who smoked during the last 3 months of pregnancy.<br>Restaurants: complete smoke-free law<br><br>Age <20: 3·40%* (−0·93 to 7·72)<br>Age 20 to 24: −0·40%* (−2·52 to 1·72)<br>Age 25 to 34: −0·40%* (−1·24 to 0·44)<br>Age 35+: 3·30%* (−0·42 to 7·02))	There were no associations between smoke-free restaurants, smoking restrictions in restaurants, or tobacco tax increases and changes in the probability of smoking during the third trimester of pregnancy.<br>A $1 increase in cigarette price was associated with a decrease in the probabilities of teenagers smoking 21 or more cigarettes per day (1 percentage point), 11 to 20 cigarettes per day (2·6 percentage points) and six to 10 cigarettes per day (2·4 percentage points)
			Restaurants: smoking restrictions (requiring designated smoking areas)<br>Age <20: 2·30%* (−1·02 to 5·61)<br>Age 20 to 24: 0·30%* (−1·25 to 1·85)<br>Age 25 to 34: 0·60%* (−0·11 to 1·31)<br>Age 35+: 2·20%* (−0·46 to 4·86)	
			Tobacco tax increase<br>Age <20: 0·03%* (−2·91 to 2·97) per USD increase in tax<br>Age 20 to 24: 1·00%* (−18·6 to 20·6) per USD increase in tax<br>Age 25 to 34: 0·40%* (−0·51 to 1·31) per USD increase in tax<br>Age 35+: −0·30%* (−1·30 to 0·70) per USD increase in tax	
Page (2012)[29]	Self-reported maternal number of cigarettes smoked per day during lifetime, dichotomised as yes or no.	Colorado birth registry	Smoking prevalence pre-ban: 16·64% (I), 8 ·66% (C); Smoking prevalence post-ban: 15·07% (I), 11·89% (C)	There was no significant change in smoking amongst mothers following the introduction of smoke-free legislation in the intervention area. The control area had a significant increase in number of smokers (p<0·0001) during the same time period.

**Maternal smoking after delivery**				
**Adams (2013)**[1]	Self-reported maternal smoking before pregnancy, during pregnancy, and after delivery.	PRAMS	1. Medicaid cessation coverage of: nicotine replacement therapy, medication, and counselling<br>Group 2 – entered Medicaid during pregnancy: 0·8%* (−1·0 to 2·6)<br><br>Group 3 – enrolled in Medicaid either before or during pregnancy: 0·5%* (−1·1 to 2·1)	Expansion of Medicaid covered smoking cessation services was not associated with significant changes in maternal smoking after delivery.
			2. Medicaid cessation coverage of: nicotine replacement therapy and medication, but no counselling<br>Group 2 – entered Medicaid during pregnancy: 0·3%* (−2·4 to 3·0)<br><br>Group 3 – enrolled in Medicaid either before or during pregnancy: 0·2%* (−1·4 to 1·8)	
			3. Some Medicaid cessation coverage of nicotine replacement therapy, medications, and counselling not already represented in categories 1 or 2<br>Group 2 – entered Medicaid during pregnancy: 1·6%* (−0.2 to 3.4)<br><br>Group 3 – enrolled in Medicaid either before or during pregnancy: 1·4%* (−0·6 to 3·4)	
**Adult smoking behaviour**				
Ma (2013)[38]	Current smoking prevalence of adults aged 18 to 39 years old and 40 years or older	Pennsylvania Health Care Cost Containment Council (PHC4) hospital discharge database	1st tax increase initial change: −0 ·52% (p = 0 ·73), 1st tax increase initial trend: 0 ·21% (p = 0 ·54); 2nd tax increase initial change: −1 ·75% (p = 0 ·14), 2nd tax increase initial trend: −0 ·46% (p = 0 ·18)<br><br>Note: 95% confidence intervals could not be calculated for these changes.	There were no significant changes in smoking prevalence following either tax increase in all adults. When examined by age, the second tax increase in January 2004 was associated with an initial 5.2% decrease and an additional 1·4% quarterly decrease in smoking prevalence in 18 to 39 year olds.

* Percentage points; GW = Gestational week; PRAMS = Pregnancy Risk Assessment Monitoring System

References

1. Adams EK, Markowitz S, Dietz PM, Tong VT. Expansion of Medicaid covered smoking cessation services: maternal smoking and birth outcomes. *Medicare Medicaid Res Rev* 2013; **3**(3): e1–23.
2. Amaral M. The effect of local smoking ordinances on fetal development: evidence from California. University of the Pacific; 2009.
3. Bakolis I, Kelly R, Fecht D, et al. Protective effects of smoke-free legislation on birth outcomes in England - a regression discontinuity design. *Epidemiology* 2016; **27**(6): 810–8.
4. Bartholomew KS, Abouk R. The effect of local smokefree regulations on birth outcomes and prenatal smoking. *Matern Child Health J* 2016; **20**(7): 1526–38.
5. Been JV, Mackay DF, Millett C, Pell JP, van Schayck OCP, Sheikh A. Impact of smoke-free legislation on perinatal and infant mortality: a national quasi-experimental study. *Sci Rep* 2015; **5**: 13020.
6. Been JV, Millett C, Lee JT, van Schayck CP, Sheikh A. Smoke-free legislation and childhood hospitalisations for respiratory tract infections. *Eur Respir J* 2015; **46**(3): 697–706.
7. Been JV, Szatkowski L, van Staa TP, et al. Smoke-free legislation and the incidence of paediatric respiratory infections and wheezing/asthma: interrupted time series analyses in the four UK nations. *Sci Rep* 2015; **5**: 15246.
8. Bharadwaj P, Johnsen JV, Loken KV. Smoking bans, maternal smoking and birth outcomes. *J Public Econ* 2014; **115**: 72–93.
9. Briggs RJ, Green T. The impact of smoking bans on birth weight: is less more? In: Essays on the economics of indoor and outdoor environments [dissertation]. Texas, USA: The University of Texas at Austin, 2009; 64–96.
10. Ciaccio CE, Gurley-Calvez T, Shireman TI. Indoor tobacco legislation is associated with fewer emergency department visits for asthma exacerbation in children. *Ann Allergy Asthma Immunol* 2016; **117**(6): 641–645.
11. Cox B, Martens E, Nemery B, Vangronsveld J, Nawrot TS. Impact of a stepwise introduction of smoke-free legislation on the rate of preterm births: analysis of routinely collected birth data. *BMJ* 2013; **346**: f441.
12. Croghan IT, Ebbert JO, Hays JT, et al. Impact of a countywide smoke-free workplace law on emergency department visits for respiratory diseases: a retrospective cohort study. *BMC Pulm Med* 2015; **15**(6): 1–8.
13. Galán I, Simón L, Boldo E, et al. Changes in hospitalizations for chronic respiratory diseases after two successive smoking bans in Spain. *PLoS One* 2017; **12**(5): e0177979.
14. Gao J, Baughman RA. Do smoking bans improve infant health? Evidence from U.S. births: 1995–2009. *East Econ J* 2017; **43**(3): 472-495.
15. Gaudreau K, Sanford CJ, Cheverie C, McClure C. The effect of a smoking ban on hospitalization rates for cardiovascular and respiratory conditions in Prince Edward Island, Canada. *PLoS One* 2013; **8**(3): e56102.
16. Hade E. Analysis of the association between birth outcomes and the Ohio tobacco ban. In: Analyses of the impact of the Ohio smoke-free workplace act. Columbus (OH): Ohio Department of Health, 2011; 24–29. http://www.rosscountyhealth.com/brochures/ODHAnalysis.pdf (accessed 05 April 2017).
17. Hajdu T, Hajdu G. Smoking ban and health at birth [preliminary discussion paper]. Budapest, Hungary: Institute of Economics, Centre for Economic and Regional Studies, Hungarian Academy of Sciences, 2017. http://real.mtak.hu/49950/1/MTDP1706.pdf (accessed 29 June 2017).
18. Hankins S, Tarasenko Y. Do smoking bans improve neonatal health? *Health Serv Res* 2016; **51**(5): 1858–78.
19. Hawkins SS, Baum CF, Oken E, Gillman MW. Associations of tobacco control policies with birth outcomes. *JAMA Pediatr* 2014; **168**(11): 186–96.
20. Hawkins SS, Hristakeva S, Gottlieb M, Baum CF. Reduction in emergency department visits for children's asthma, ear infections, and respiratory infections after the introduction of state smoke-free legislation. *Prev Med* 2016; **89**: 278–85.
21. Kabir Z, Daly S, Clarke V, Keogan S, Clancy L. Smoking ban and small-for-gestational age births in Ireland. *PLoS One* 2013; **8**(3): e57441.
22. Landers G. The impact of smoke-free laws on asthma discharges: a multistate analysis. *Am J Public Health* 2014; **104**(2): e74–9.
23. Lee SL, Wong WH, Lau YL. Smoke-free legislation reduces hospital admissions for childhood lower respiratory tract infection. *Tob Control* 2016; **25**(e2): e90–4.
24. Mackay D, Haw S, Ayres JG, Fischbacher C, Pell JP. Smoke-free legislation and hospitalizations for childhood asthma. *New Engl J Med* 2010; **363**(12): 1139–45.
25. Mackay DF, Nelson SM, Haw SJ, Pell JP. Impact of Scotland's smoke-free legislation on pregnancy complications: retrospective cohort study. *PLoS Med* 2012; **9**(3): e1001175.

26. Markowitz S, Adams EK, Dietz PM, Kannan V, Tong VT. Tobacco control policies, birth outcomes, and maternal human capital. *J Hum Cap* 2013; **7**(2): 130–60.
27. McKinnon B, Auger N, Kaufman JS. The impact of smoke-free legislation on educational differences in birth outcomes. *J Epidemiol Community Health* 2015; **69**(10): 937–43.
28. Millett C, Lee JT, Laverty AA, Glantz SA, Majeed A. Hospital admissions for childhood asthma after smoke-free legislation in England. *Pediatrics* 2013; **131**(2): e495–501.
29. Page RL, Slejko JF, Libby AM. A citywide smoking ban reduced maternal smoking and risk for preterm births: a colorado natural experiment. *J Women's Health* 2012; **21**(6): 621–7.
30. Peelen MJ, Sheikh A, Kok M, et al. Tobacco control policies and perinatal health: a national quasi-experimental study. *Sci Rep* 2016; **6**: 23907.
31. Rayens MK, Burkhart PV, Zhang M, et al. Reduction in asthma-related emergency department visits after implementation of a smoke-free law. *J Allergy Clin Immunol* 2008; **122**(3): 537–41.
32. Shetty KD, DeLeire T, White C, Bhattacharya J. Changes in U.S. hospitalization and mortality rates following smoking bans. *J Pol Anal Manag* 2011; **30**(1): 6–28.
33. Simón L, Pastor-Barriuso R, Boldo E, et al. Smoke-free legislation in Spain and prematurity. *Pediatrics* 2017; doi: http://dx.doi.org/10.1542/peds.2016-2068.
34. Vicedo-Cabrera AM, Schindler C, Radovanovic D, et al. Benefits of smoking bans on preterm and early-term births: a natural experimental design in Switzerland. *Tob Control* 2016; **25**(e2): e135–41.
35. Vicedo-Cabrera AM, Röösli M, Radovanovic D, et al. Cardiorespiratory hospitalisation and mortality reductions after smoking bans in Switzerland. *Swiss Med Wkly* 2017; **146**: w14381.
36. Bhai M. Understanding the gradient in childhood asthma: the effect of cigarette taxes on inequality. Illinois, USA: University of Illinois at Chicago, 2015. https://static1.squarespace.com/static/55d7d665e4b022811c4df8b3/t/566e52daa12f444cd6e3f3c4/1450070746306/AsthmaJMP112215.pdf (accessed 05 April 2017).
37. Evans WN, Ringel JS. Can higher cigarette taxes improve birth outcomes? *J Public Econ* 1999; **72**: 135–54.
38. Ma ZQ, Kuller LH, Fisher MA, Ostroff SM. Use of interrupted time-series method to evaluate the impact of cigarette excise tax increases in Pennsylvania, 2000-2009. *Prev Chronic Dis* 2013; **10**: e169.
39. Patrick SW, Warner KE, Pordes E, Davis MM. Cigarette tax increase and infant mortality. *Pediatrics* 2016; **137**(1): 1–8.
40. Sen A, Pierard E. Estimating the effects of cigarette taxes on birth outcomes. *Can Public Policy* 2011; **37**(2): 257–76.
41. Jarlenski M, Bleich SN, Bennett WL, Stuart EA, Barry CL. Medicaid enrollment policy increased smoking cessation among pregnant women but had no impact on birth outcomes. *Health Aff* 2014; **33**(6): 997–1005.
42. Bianchi M, Campi R, Bonati M. Smoke-free legislation and asthma. *N Eng J Med* 2011; **364**(1): 87–8.
43. Dove MS, Dockery DW, Connolly GN. Smoke-free air laws and asthma prevalence, symptoms, and severity among nonsmoking youth. *Pediatrics* 2011; **127**(1): 102–9.
44. Kabir Z, Clarke V, Conroy R, McNamee E, Daly S, Clancy L. Low birthweight and preterm birth rates 1 year before and after the Irish workplace smoking ban. *BJOG* 2009; **116**(13): 1782–7.
